# Supplementary material for: Predictions from standard epidemiological models of consequences of segregating and isolating vulnerable people into care facilities
Source: PLoS One. 2023 Oct 30;18(10):e0293556. doi: 10.1371/journal.pone.0293556 (PMC10615287; doi:10.1371/journal.pone.0293556)
Supplement: S1 Appendix — (PDF) [file pone.0293556.s001.pdf]

S1 Appendix

Supporting Information for the article "Predictions from standard epidemiological models of consequences of segregating and isolating vulnerable people into care facilities" by Joseph Hickey and Denis G. Rancourt

Table of Contents

Table of Symbols 2

Appendix A: Additional results for P_r = 0.95 3

A.1: Attack-rate contour maps for different values of λ........ 4

A.2: Attack-rate vs. x composite plots, for different fixed values of γ_v and λ........ 8

A.3: Attack-rate vs. γ_v composite plots, for different fixed values of x and λ........ 18

A.4: Epidemic curves for different values of x and γ_v, for λ = 1 34

Appendix B: Results for P_r = 0.99, P_r = 0.8 and P_r = 0.6 50

B.1: Attack-rate contour maps for different values of λ and for P_r = 0.99........ 51

B.2: Attack-rate contour maps for different values of λ and for P_r = 0.8........ 55

B.3: Attack-rate contour maps for different values of λ and for P_r = 0.6........ 59

Appendix C: Varying seed distribution........ 63

C.1: Attack-rate contour maps for different values of λ, for P_r = 0.99, seed = 100, and ssr = 1 64

C.2: Attack-rate contour maps for different values of λ, for P_r = 0.95, seed = 100, and ssr = 1 68

C.3: Attack-rate contour maps for different values of λ, for P_r = 0.8, seed = 100, and ssr = 1 72

C.4: Attack-rate contour maps for different values of λ, for P_r = 0.6, seed = 100, and ssr = 1 76

Appendix D: Varying seed magnitude........ 80

Table of Symbols

Symbol	Description
N_r	Total number of *r* individuals
N_v	Total number of *v* individuals
P_r	Proportion of the total population that belongs to the *r* group
λ	Coefficient modulating the relationship between c_{rv} and c_{vr}, as per Eq. 2 of the main text
γ_r	Recovery rate of infected *r* individuals
γ_v	Recovery rate of infected *v* individuals
seed	Total number of infected individuals at the outset of the simulation
ssr	Share of all seed individuals who belong to the *r* group
NI_r	Natural immunity of *r* individuals (set to 0 in all results shown in the main text and the appendices)
NI_v	Natural immunity of *v* individuals (set to 0 in all results shown in the main text and the appendices)
c_r	Frequency of contacts involving an *r* individual *
c_{rr}	Frequency of contacts between two *r* individuals *
c_{rv}	Frequency of contacts between an *r* and a *v* individual *
c_v	Frequency of contacts involving a *v* individual *
c_{vv}	Frequency of contacts between two *v* individuals *
c_{vr}	Frequency of contacts between a *v* and an *r* individual *
A_r	Attack rate among the *r* population (Eq. 3, main text)
A_v	Attack rate among the *v* population (Eq. 3, main text)
x	Degree of segregation versus intermingling of the *v* and *r* groups (Eq. 4, main text)

* Note that contact frequencies c_{ij} (i.e., c_{rr}, c_{rv}, c_{vv}, and c_{vr}) are defined such that the contact is guaranteed to result in infection when the contact is between a susceptible *i* person and an infectious *j* person, and that $c_r = c_{rr} + c_{rv}$ and $c_v = c_{vv} + c_{vr}$, as explained in the main text (Model section).

Appendix A: Additional results for P_r = 0.95

A.1: Attack-rate contour maps for different values of λ

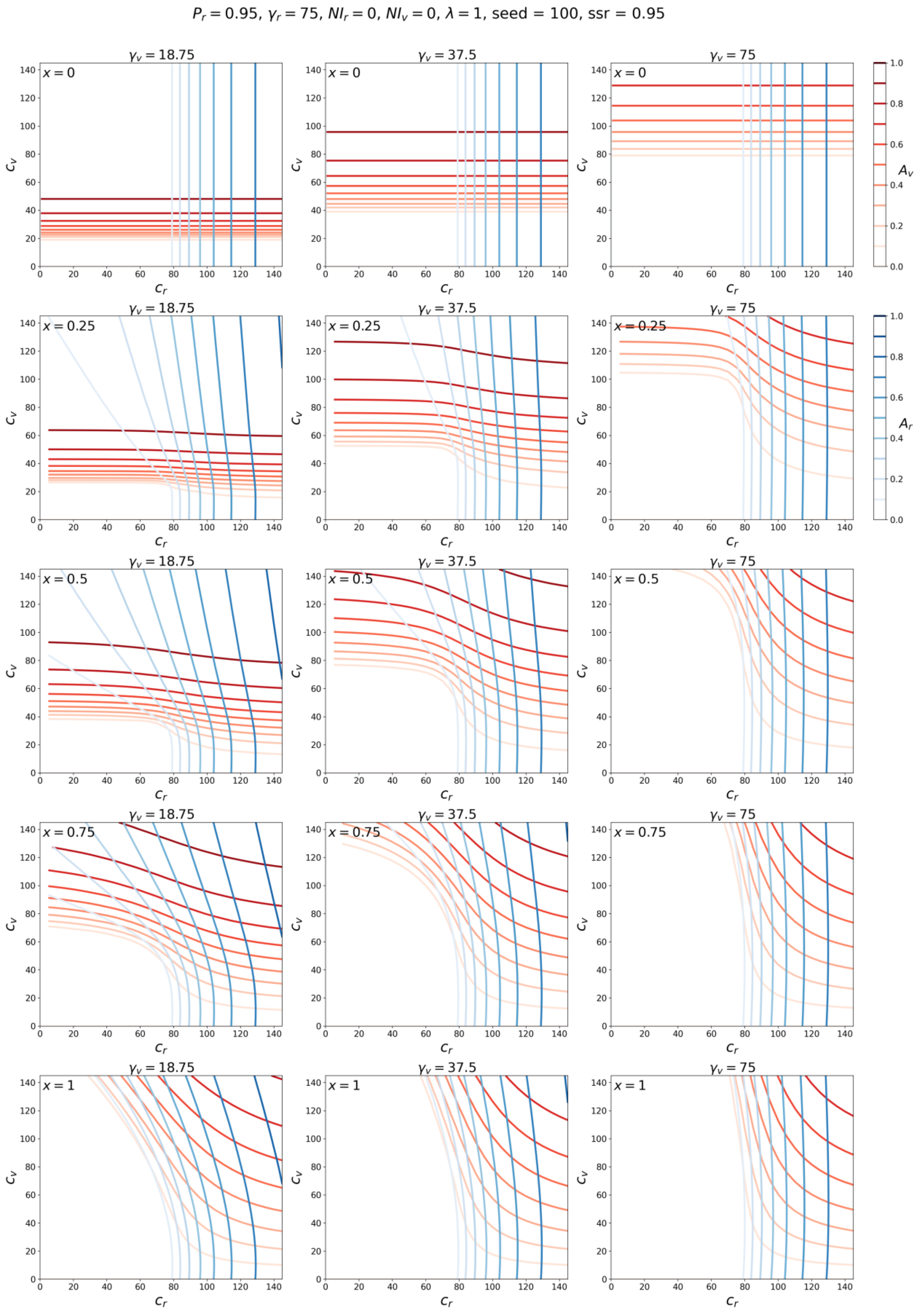
$P_r = 0.95$, $\gamma_r = 75$, $NI_r = 0$, $NI_v = 0$, $\lambda = 1$, seed = 100, ssr = 0.95
$\gamma_v = 18.75$
$\gamma_v = 37.5$
$\gamma_v = 75$
$x = 0$
$x = 0.25$
$x = 0.5$
$x = 0.75$
$x = 1$
c_v
c_r
A_v
A_r

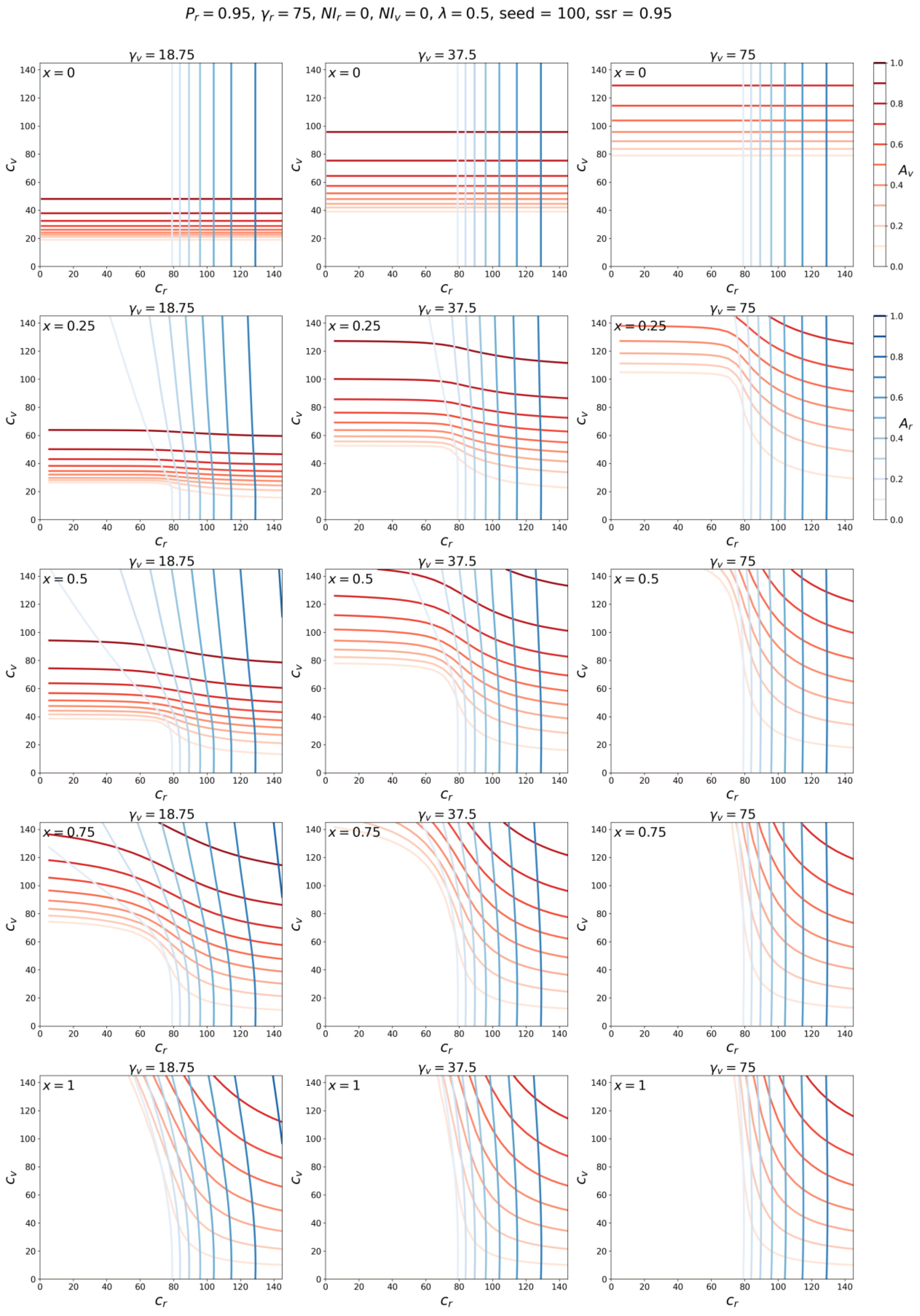

$P_r = 0.95$, $\gamma_r = 75$, $NI_r = 0$, $NI_v = 0$, $\lambda = 0.5$, seed = 100, ssr = 0.95
$\gamma_v = 18.75$
$\gamma_v = 37.5$
$\gamma_v = 75$
$x = 0$
$x = 0.25$
$x = 0.5$
$x = 0.75$
$x = 1$
c_v
c_r
A_v
A_r

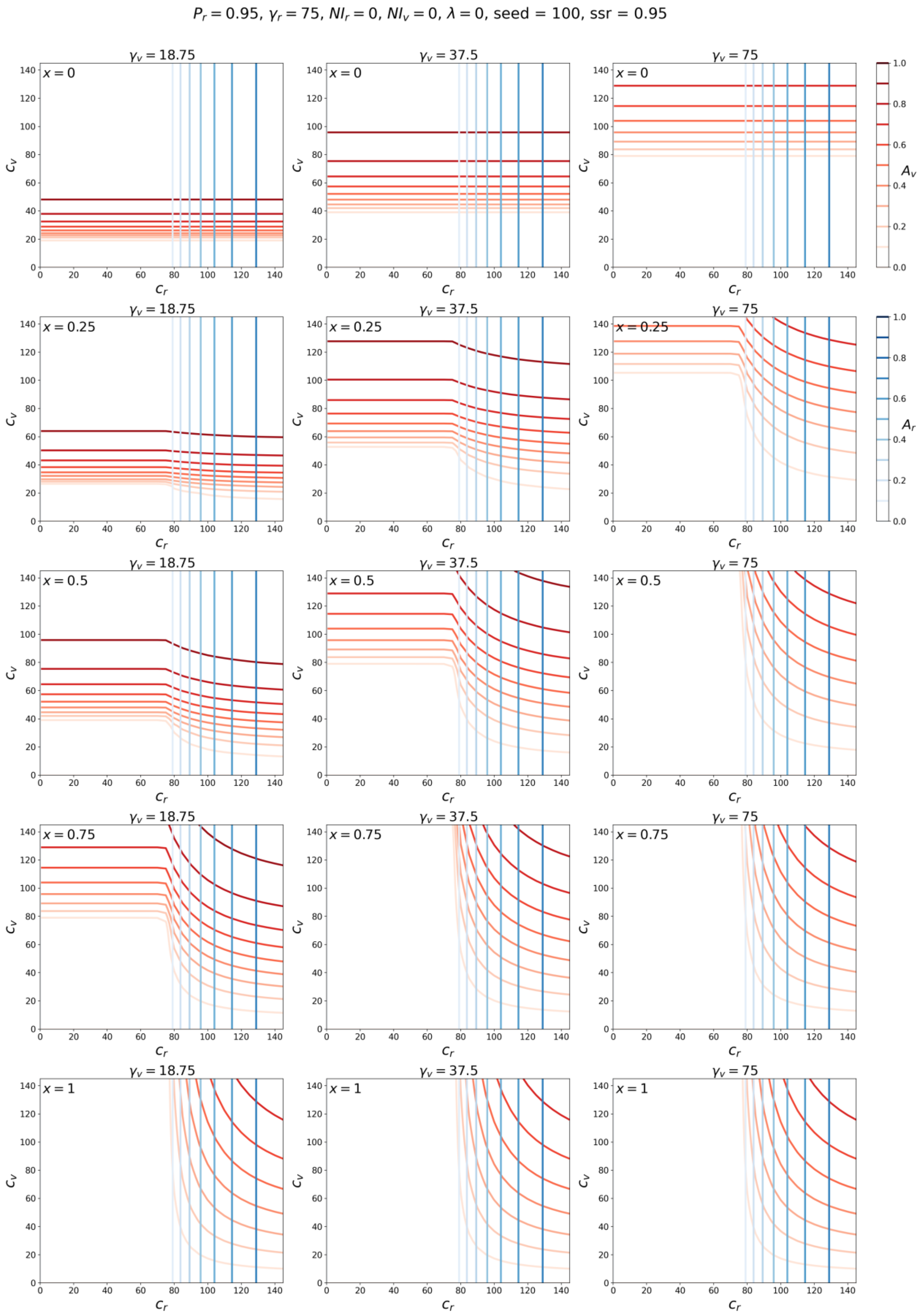
$P_r = 0.95$, $\gamma_r = 75$, $NI_r = 0$, $NI_v = 0$, $\lambda = 0$, seed = 100, ssr = 0.95
$\gamma_v = 18.75$
$\gamma_v = 37.5$
$\gamma_v = 75$
$x = 0$
$x = 0.25$
$x = 0.5$
$x = 0.75$
$x = 1$
c_v
c_r
A_v
A_r

A.2: Attack-rate vs. x composite plots, for different fixed values of γ_v and λ

$P_r = 0.95$, $NI_r = 0$, $NI_v = 0$, $\gamma_r = 75$, $\gamma_v = 18.75$, $\lambda = 1$, seed = 100, ssr = 0.95

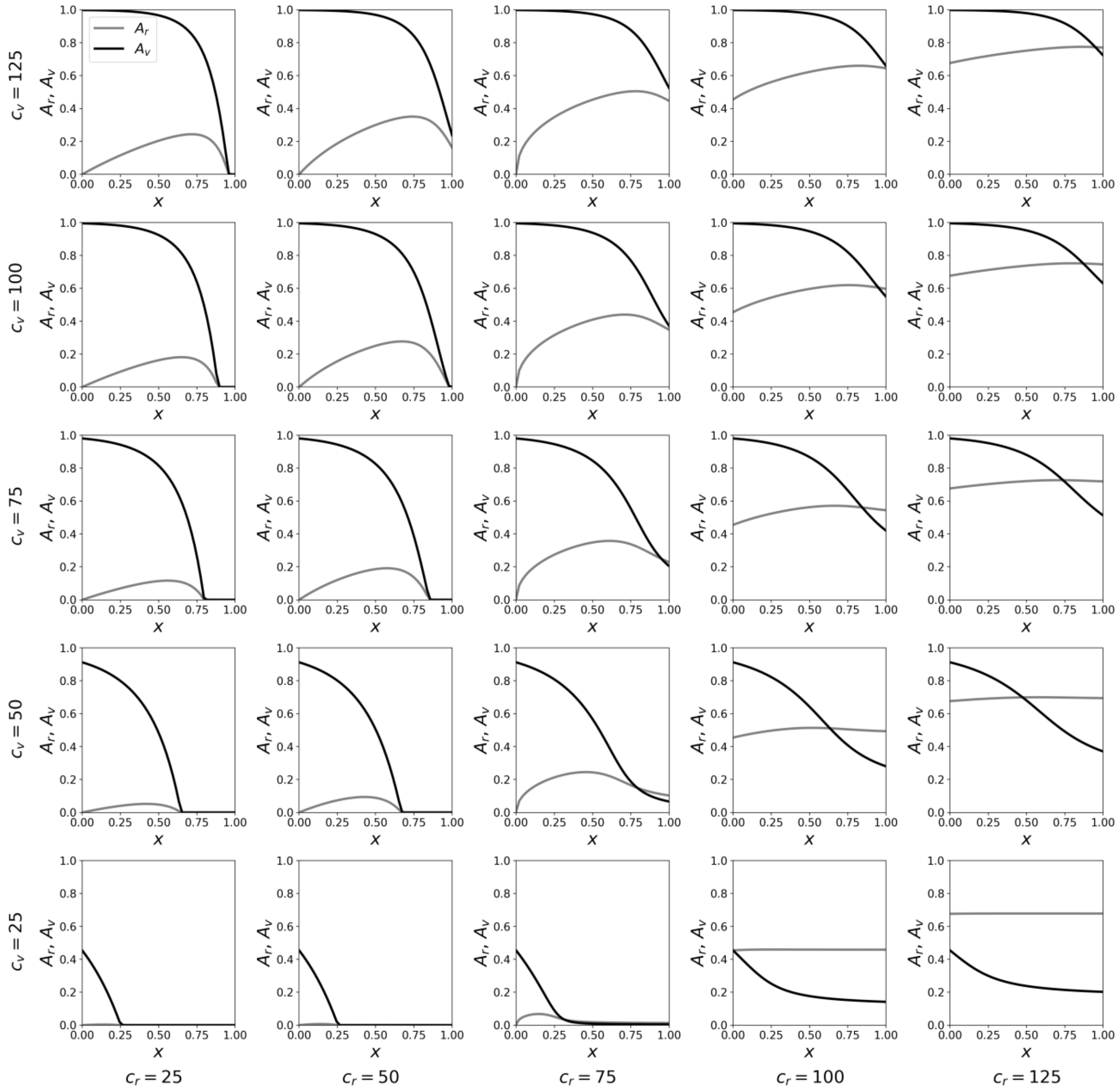

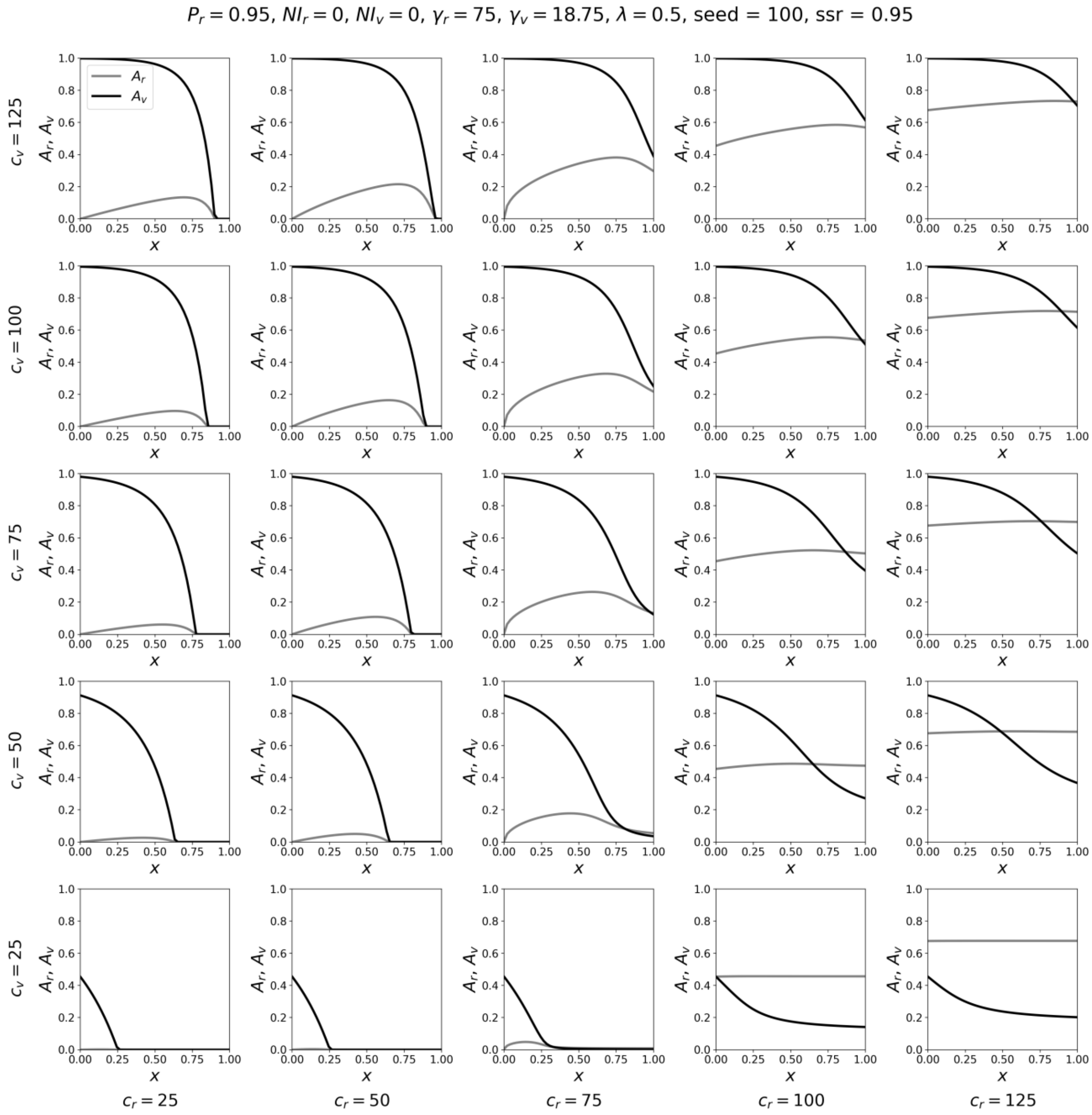
$P_r = 0.95$, $NI_r = 0$, $NI_v = 0$, $\gamma_r = 75$, $\gamma_v = 18.75$, $\lambda = 0.5$, seed = 100, ssr = 0.95
A_r
A_v
$c_v = 125$
$c_v = 100$
$c_v = 75$
$c_v = 50$
$c_v = 25$
A_r, A_v
x
$c_r = 25$
$c_r = 50$
$c_r = 75$
$c_r = 100$
$c_r = 125$

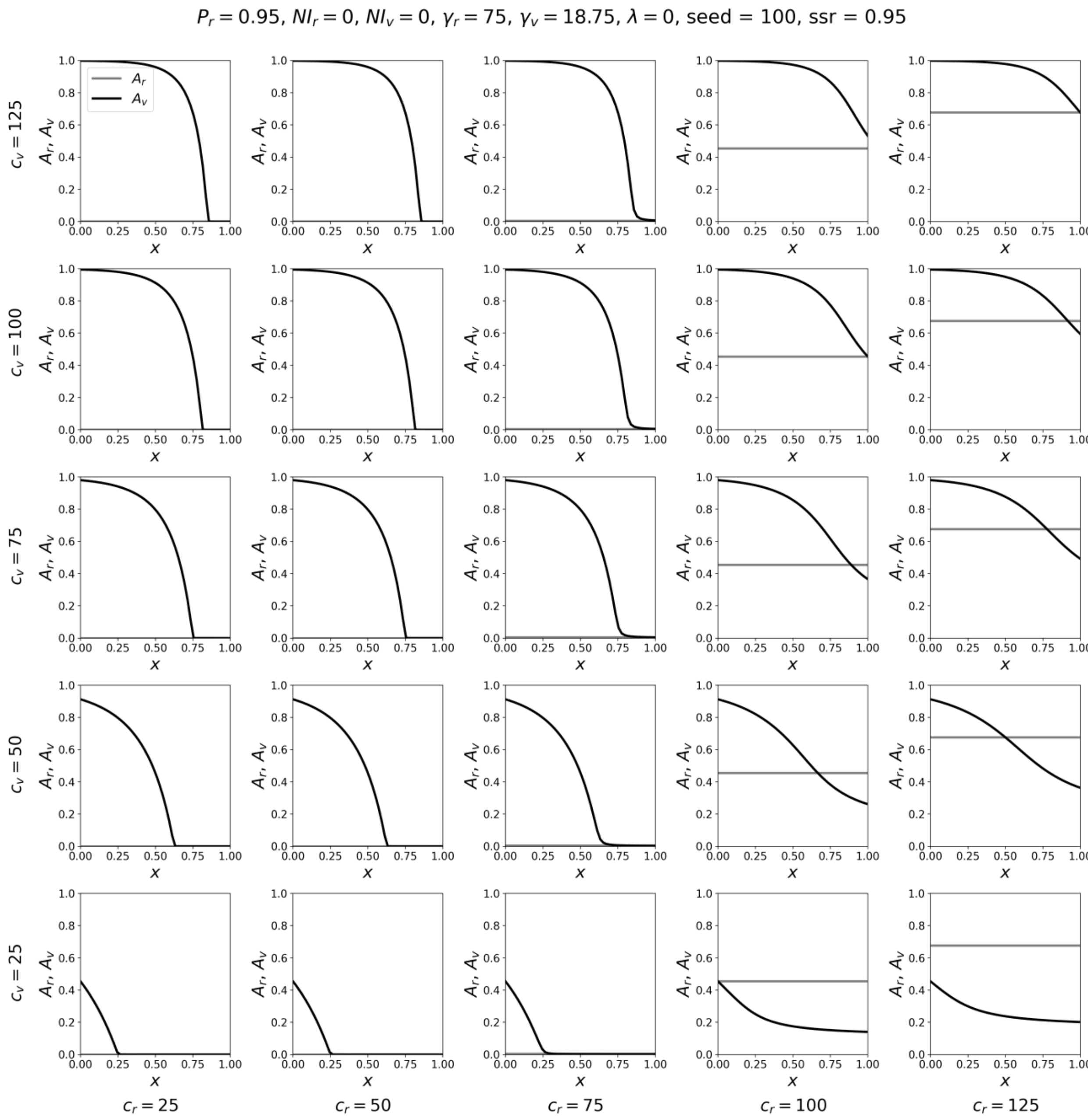
$P_r = 0.95$, $NI_r = 0$, $NI_v = 0$, $\gamma_r = 75$, $\gamma_v = 18.75$, $\lambda = 0$, seed = 100, ssr = 0.95
A_r
A_v
$c_v = 125$
$c_v = 100$
$c_v = 75$
$c_v = 50$
$c_v = 25$
A_r, A_v
x
$c_r = 25$
$c_r = 50$
$c_r = 75$
$c_r = 100$
$c_r = 125$

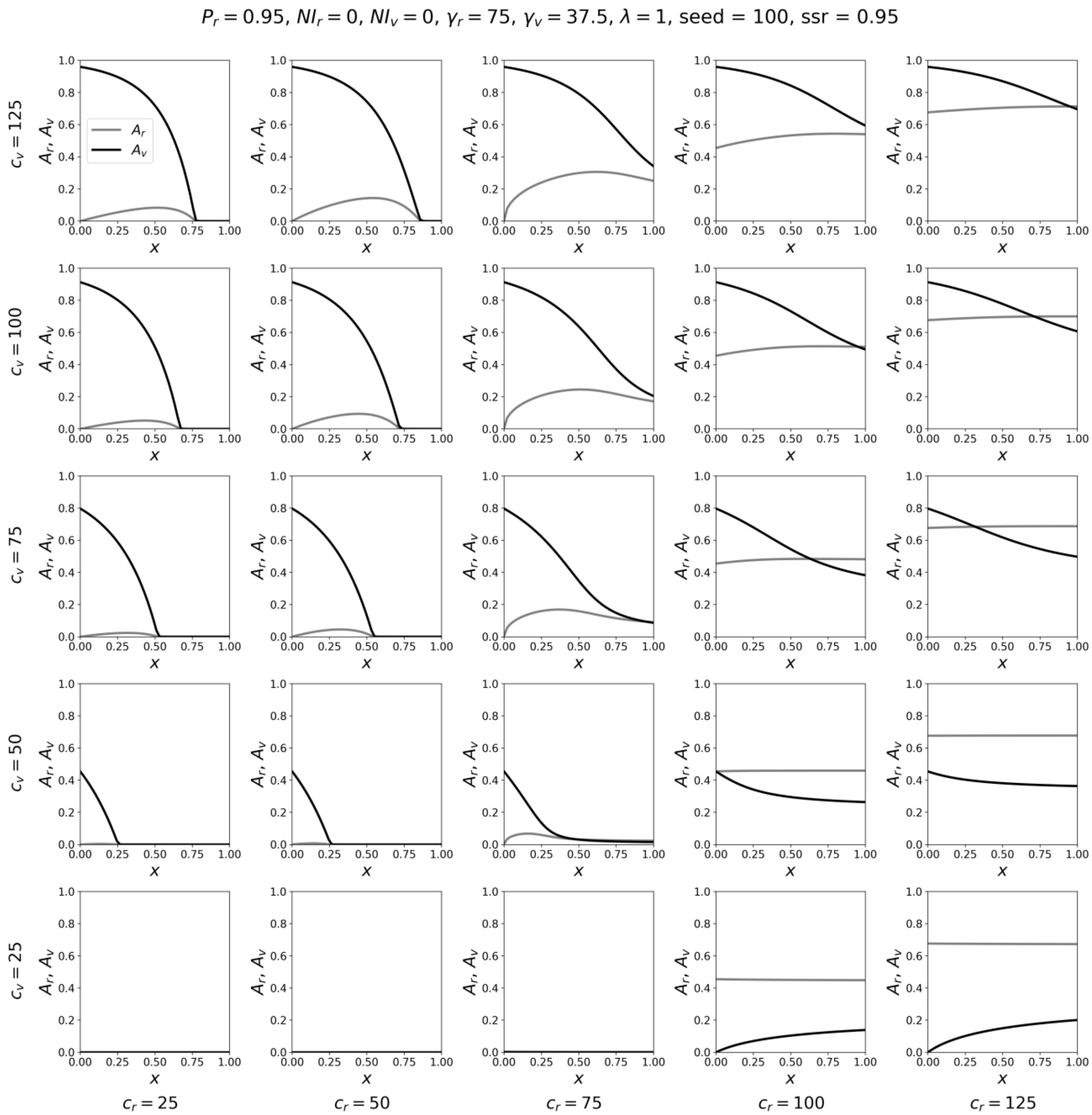
$P_r = 0.95$, $NI_r = 0$, $NI_v = 0$, $\gamma_r = 75$, $\gamma_v = 37.5$, $\lambda = 1$, seed = 100, ssr = 0.95
A_r
A_v
$c_v = 125$
$c_v = 100$
$c_v = 75$
$c_v = 50$
$c_v = 25$
A_r, A_v
x
$c_r = 25$
$c_r = 50$
$c_r = 75$
$c_r = 100$
$c_r = 125$

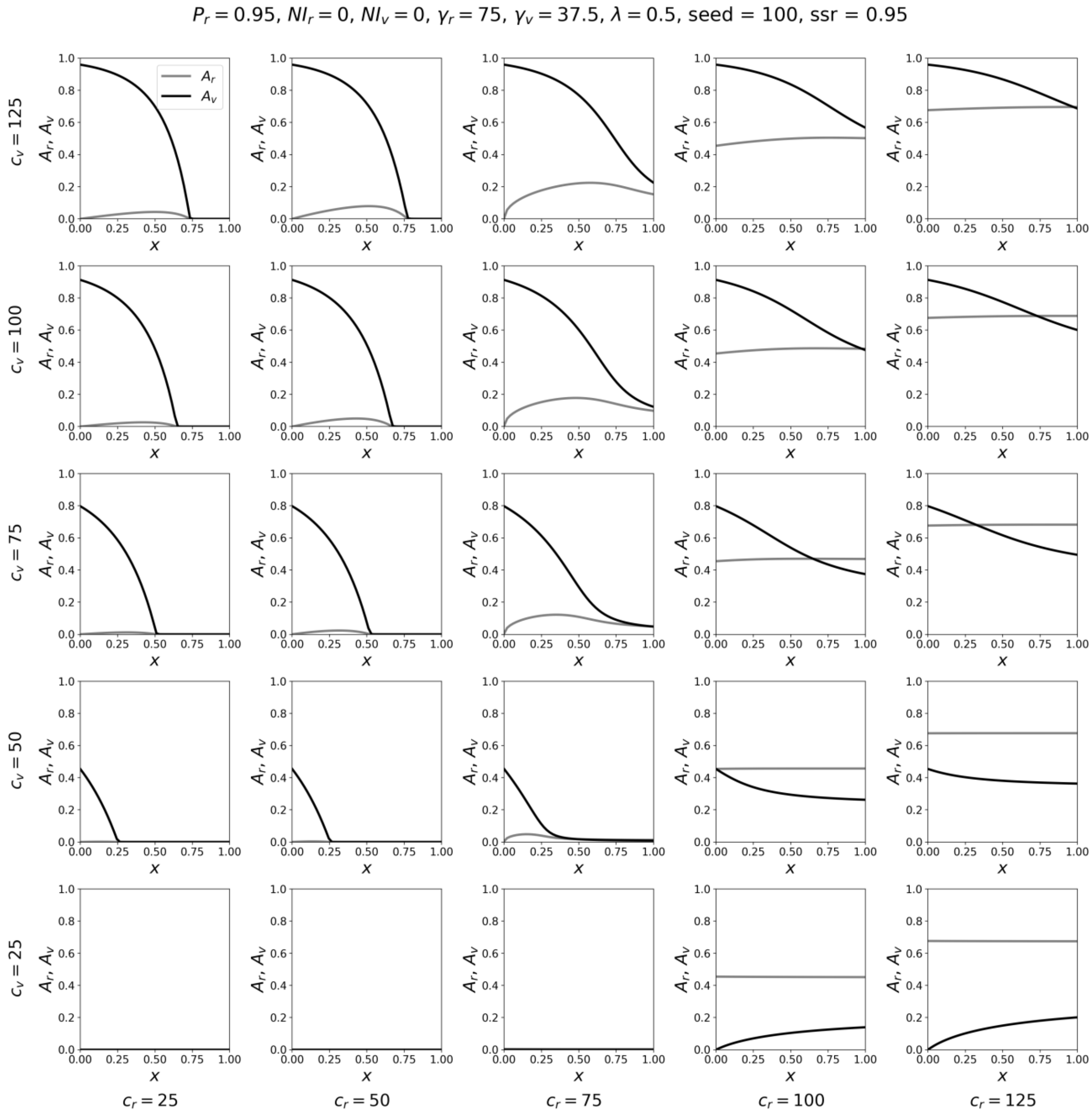
$P_r = 0.95$, $NI_r = 0$, $NI_v = 0$, $\gamma_r = 75$, $\gamma_v = 37.5$, $\lambda = 0.5$, seed = 100, ssr = 0.95
A_r
A_v
$c_v = 125$
$c_v = 100$
$c_v = 75$
$c_v = 50$
$c_v = 25$
A_r, A_v
x
$c_r = 25$
$c_r = 50$
$c_r = 75$
$c_r = 100$
$c_r = 125$

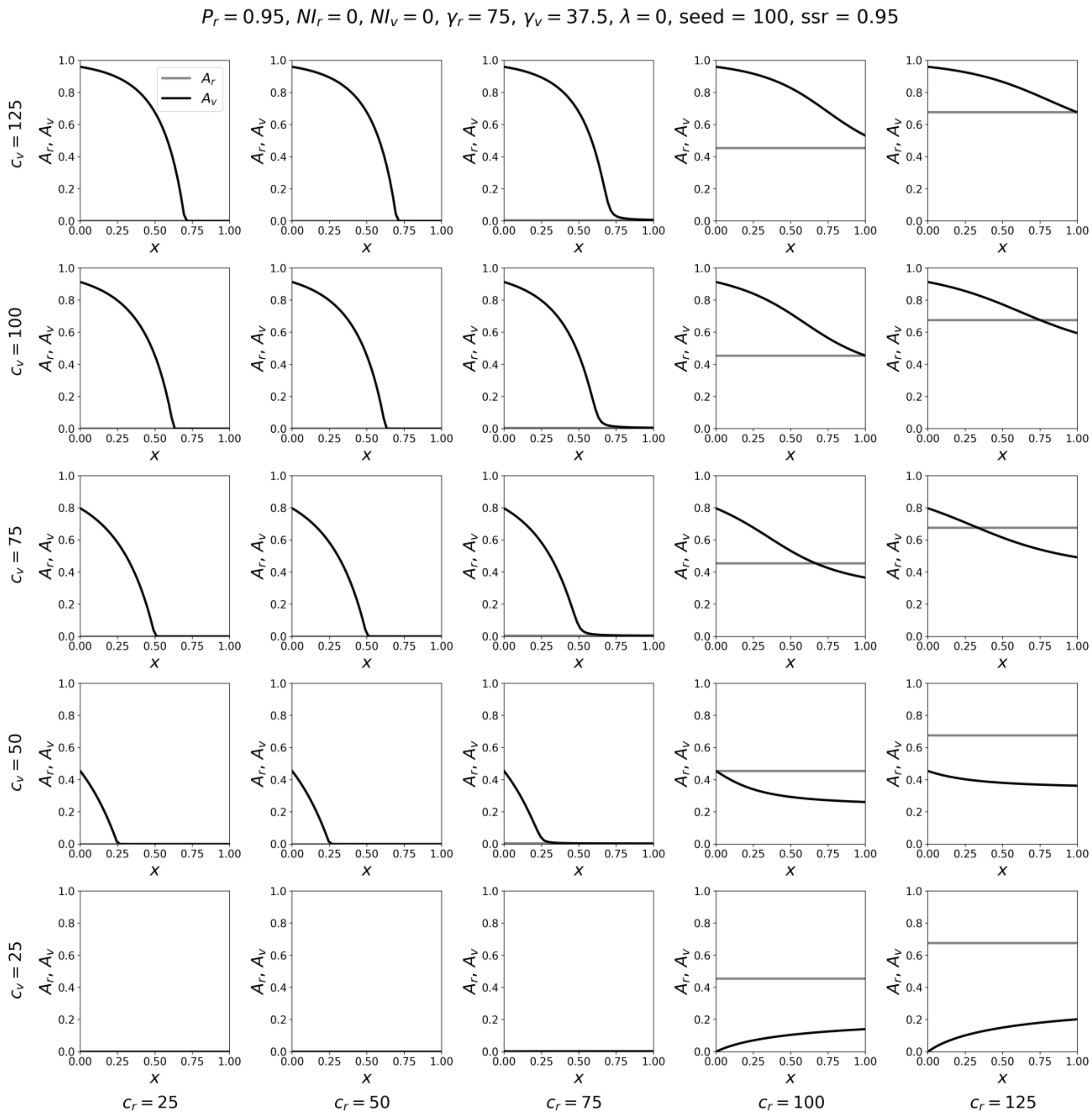
$P_r = 0.95$, $NI_r = 0$, $NI_v = 0$, $\gamma_r = 75$, $\gamma_v = 37.5$, $\lambda = 0$, seed = 100, ssr = 0.95
A_r
A_v
$c_v = 125$
$c_v = 100$
$c_v = 75$
$c_v = 50$
$c_v = 25$
A_r, A_v
x
$c_r = 25$
$c_r = 50$
$c_r = 75$
$c_r = 100$
$c_r = 125$

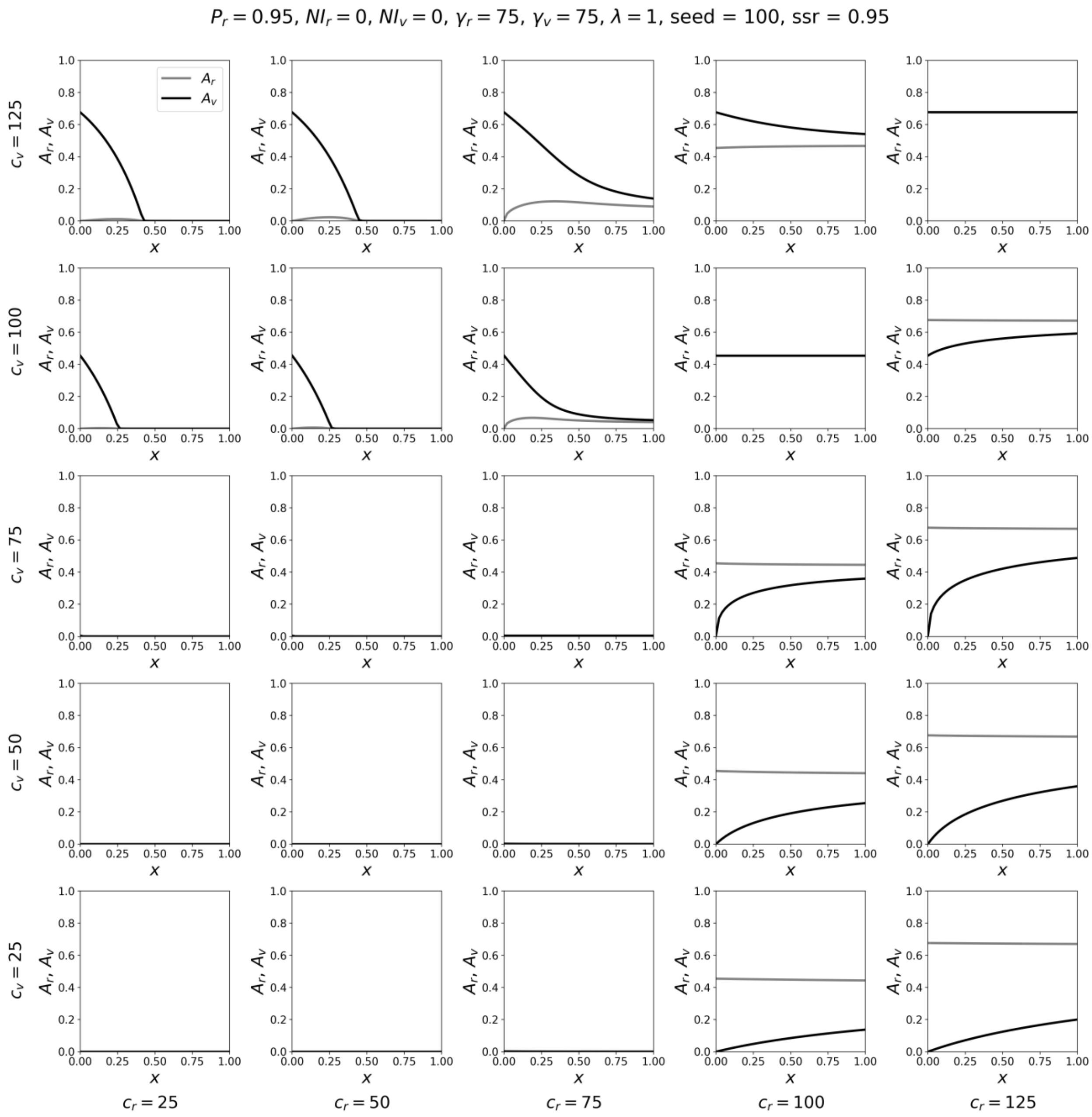

$P_r = 0.95$, $NI_r = 0$, $NI_v = 0$, $\gamma_r = 75$, $\gamma_v = 75$, $\lambda = 1$, seed = 100, ssr = 0.95
A_r
A_v
$c_v = 125$
$c_v = 100$
$c_v = 75$
$c_v = 50$
$c_v = 25$
A_r, A_v
x
$c_r = 25$
$c_r = 50$
$c_r = 75$
$c_r = 100$
$c_r = 125$

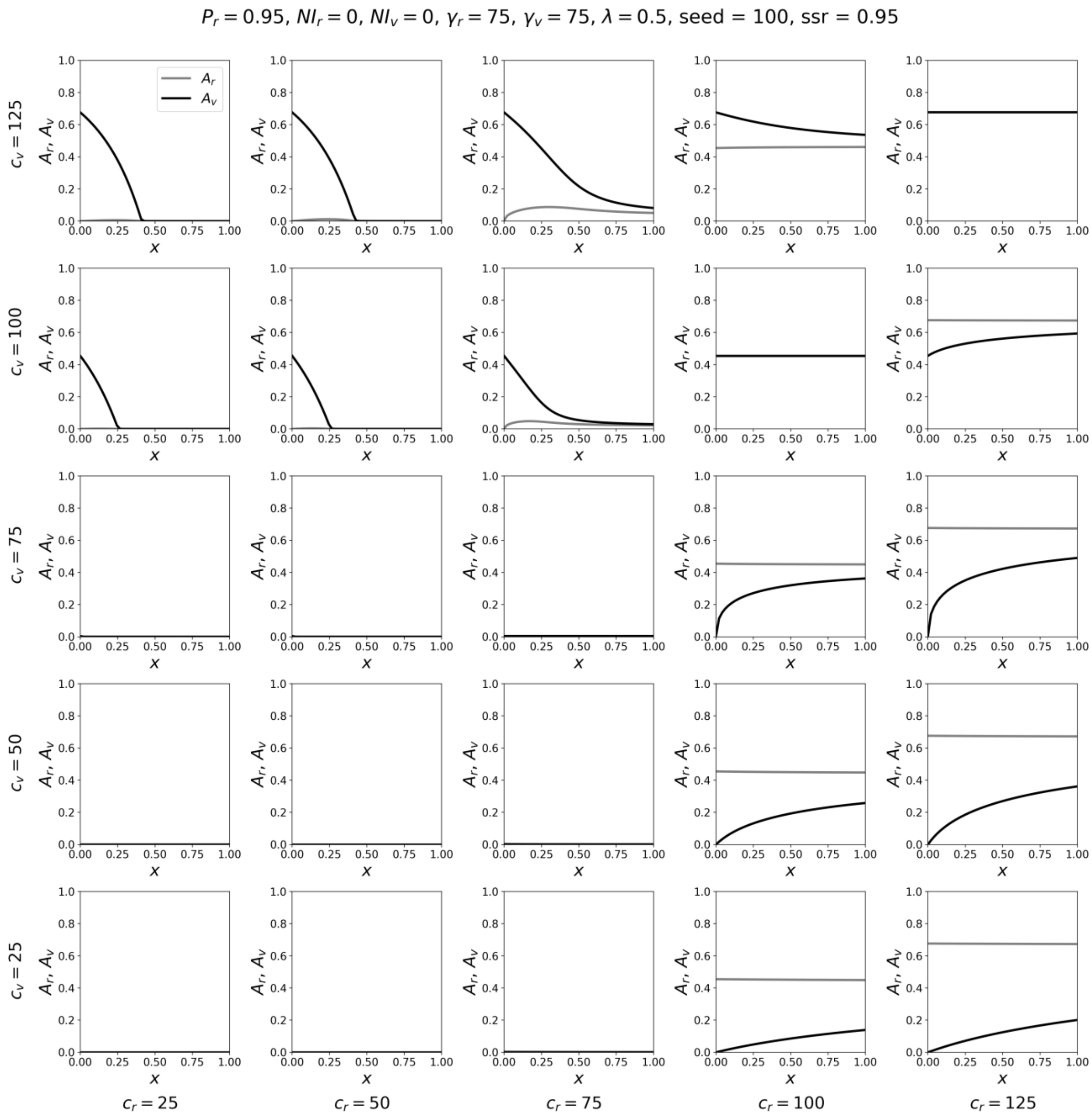

$P_r = 0.95$, $NI_r = 0$, $NI_v = 0$, $\gamma_r = 75$, $\gamma_v = 75$, $\lambda = 0.5$, seed = 100, ssr = 0.95
A_r
A_v
$c_v = 125$
$c_v = 100$
$c_v = 75$
$c_v = 50$
$c_v = 25$
A_r, A_v
x
$c_r = 25$
$c_r = 50$
$c_r = 75$
$c_r = 100$
$c_r = 125$

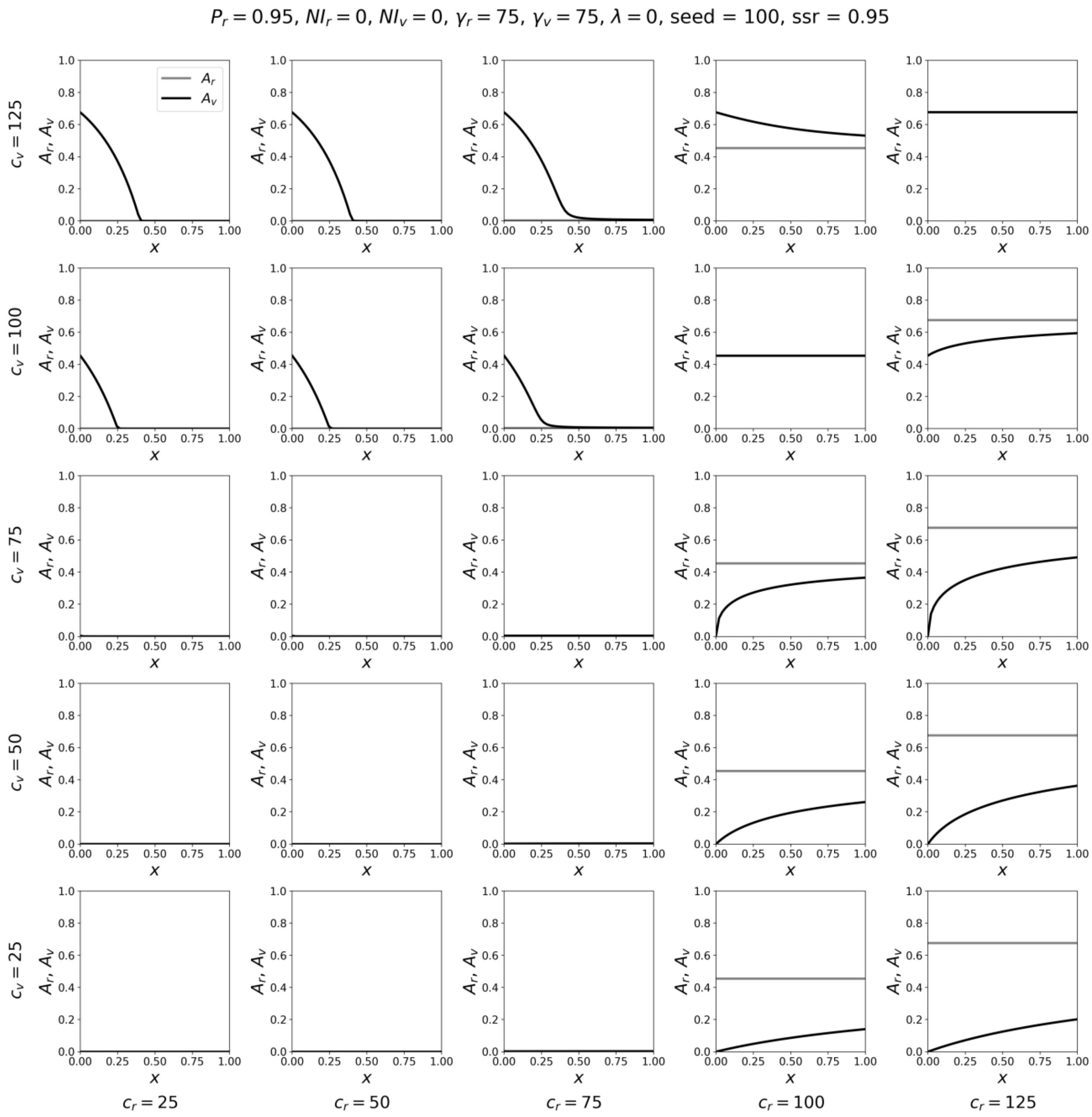

$P_r = 0.95$, $NI_r = 0$, $NI_v = 0$, $\gamma_r = 75$, $\gamma_v = 75$, $\lambda = 0$, seed = 100, ssr = 0.95
A_r
A_v
$c_v = 125$
$c_v = 100$
$c_v = 75$
$c_v = 50$
$c_v = 25$
A_r, A_v
x
$c_r = 25$
$c_r = 50$
$c_r = 75$
$c_r = 100$
$c_r = 125$

A.3: Attack-rate vs. γ_v composite plots, for different fixed values of x *and λ*

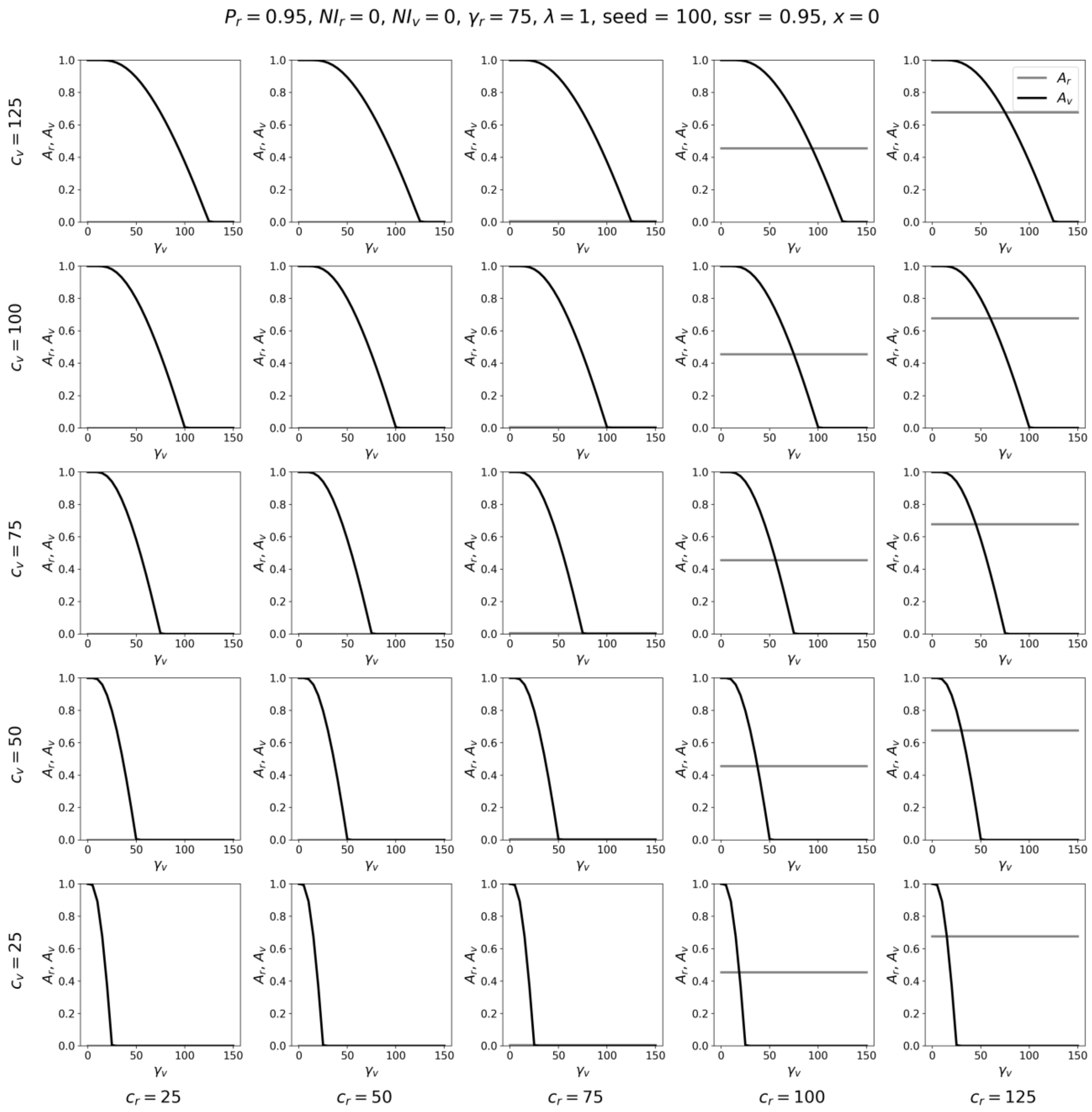
$P_r = 0.95$, $NI_r = 0$, $NI_v = 0$, $\gamma_r = 75$, $\lambda = 1$, seed = 100, ssr = 0.95, $x = 0$
A_r
A_v
$c_v = 125$
$c_v = 100$
$c_v = 75$
$c_v = 50$
$c_v = 25$
A_r, A_v
γ_v
$c_r = 25$
$c_r = 50$
$c_r = 75$
$c_r = 100$
$c_r = 125$

$P_r = 0.95$, $NI_r = 0$, $NI_v = 0$, $\gamma_r = 75$, $\lambda = 1$, seed = 100, ssr = 0.95, $x = 0.25$

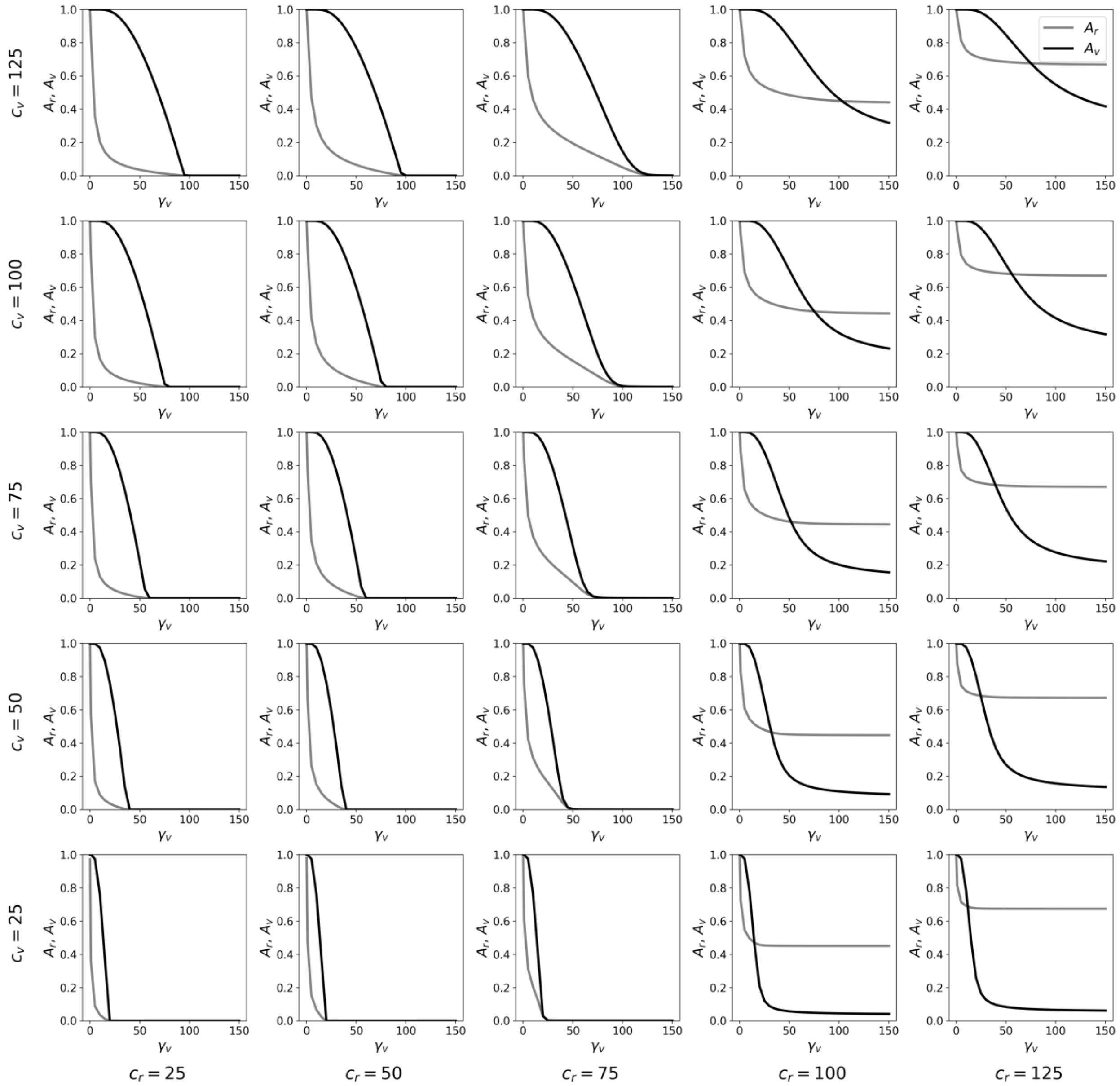

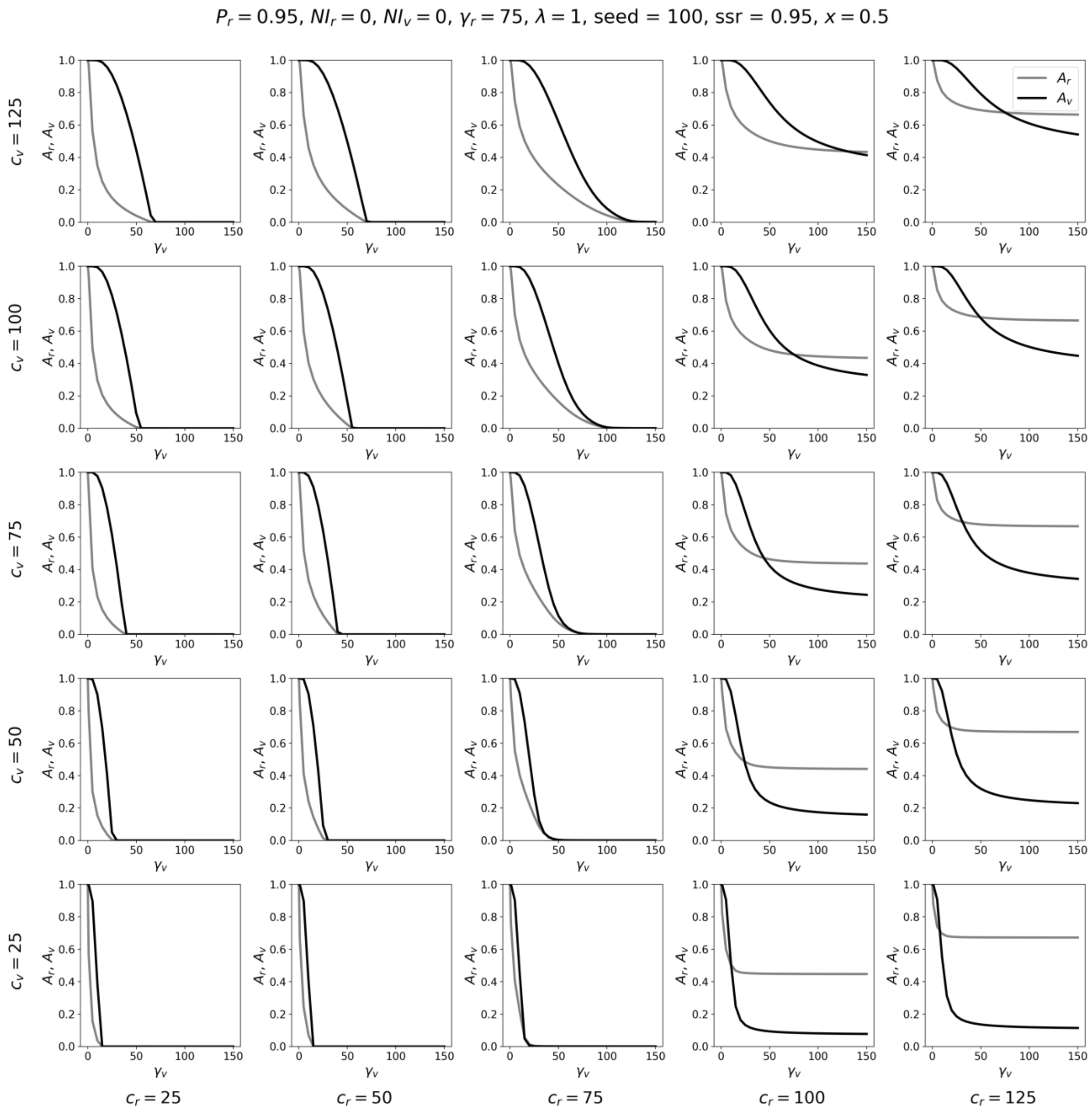

$P_r = 0.95$, $NI_r = 0$, $NI_v = 0$, $\gamma_r = 75$, $\lambda = 1$, seed = 100, ssr = 0.95, $x = 0.5$
$c_v = 125$
$c_v = 100$
$c_v = 75$
$c_v = 50$
$c_v = 25$
$c_r = 25$
$c_r = 50$
$c_r = 75$
$c_r = 100$
$c_r = 125$
A_r, A_v
γ_v
A_r
A_v

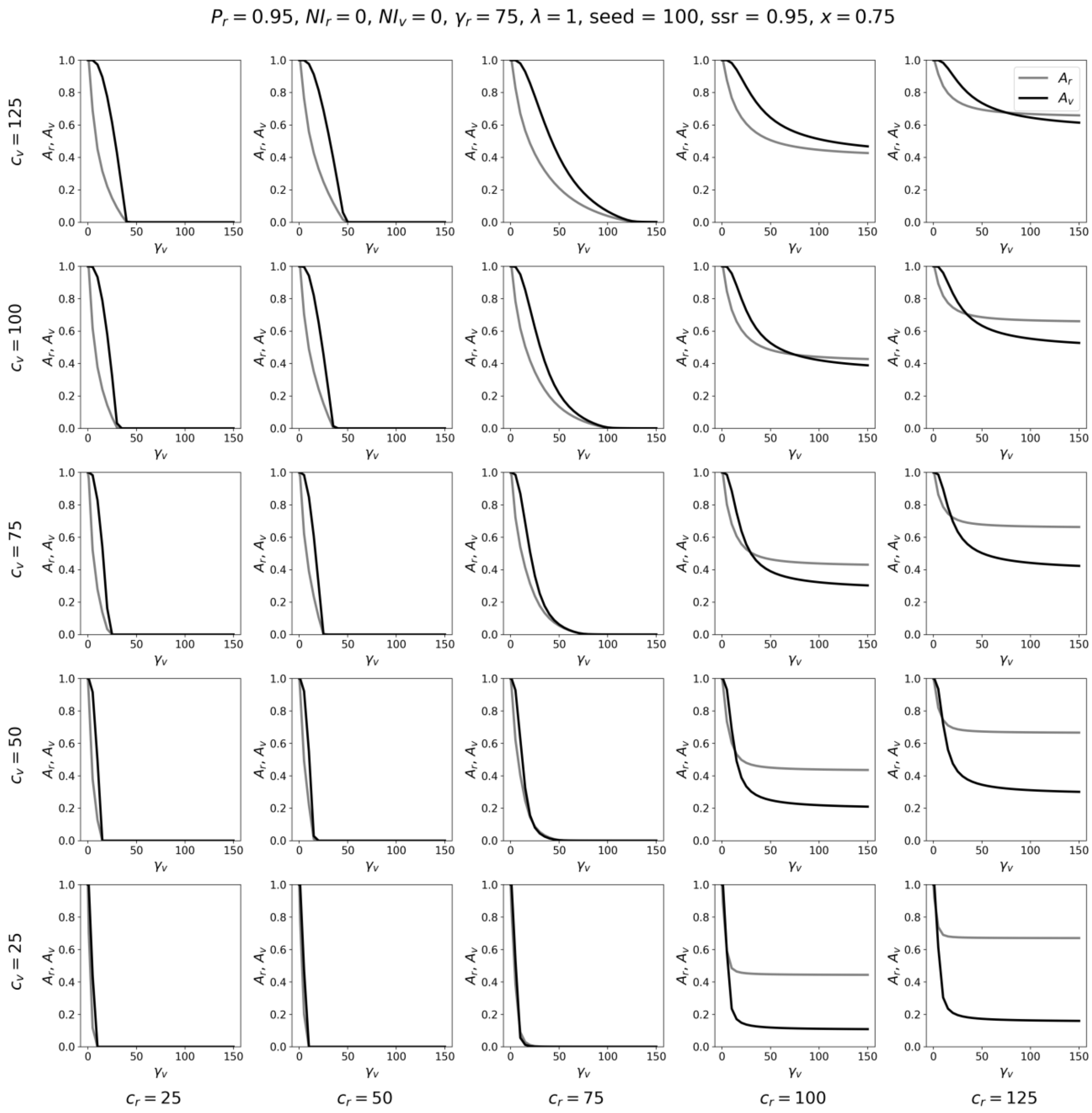
$P_r = 0.95$, $NI_r = 0$, $NI_v = 0$, $\gamma_r = 75$, $\lambda = 1$, seed = 100, ssr = 0.95, $x = 0.75$
$c_v = 125$
$c_v = 100$
$c_v = 75$
$c_v = 50$
$c_v = 25$
$c_r = 25$
$c_r = 50$
$c_r = 75$
$c_r = 100$
$c_r = 125$
A_r, A_v
γ_v
A_r
A_v

$P_r = 0.95$, $NI_r = 0$, $NI_v = 0$, $\gamma_r = 75$, $\lambda = 1$, seed = 100, ssr = 0.95, $x = 1$

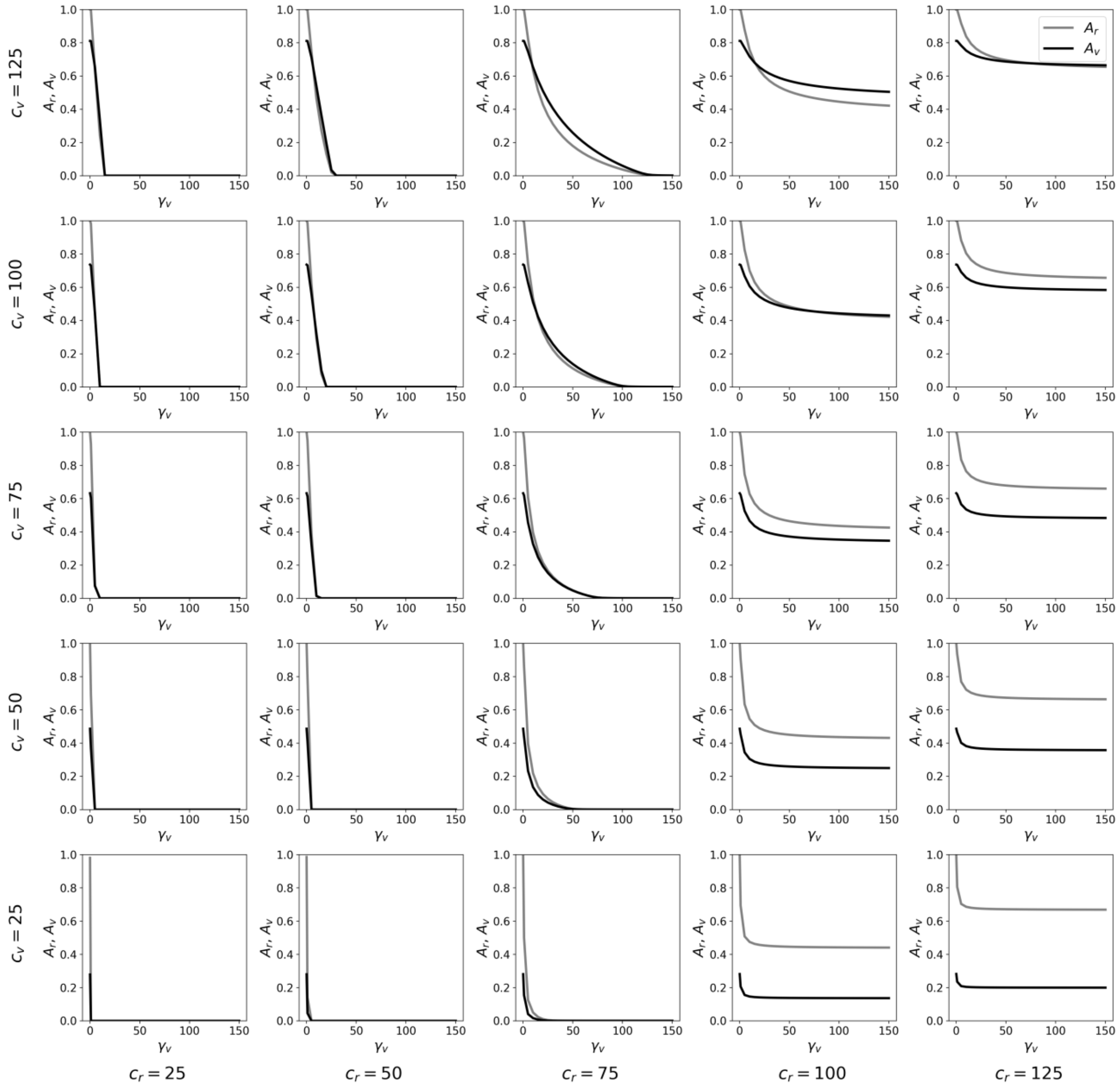

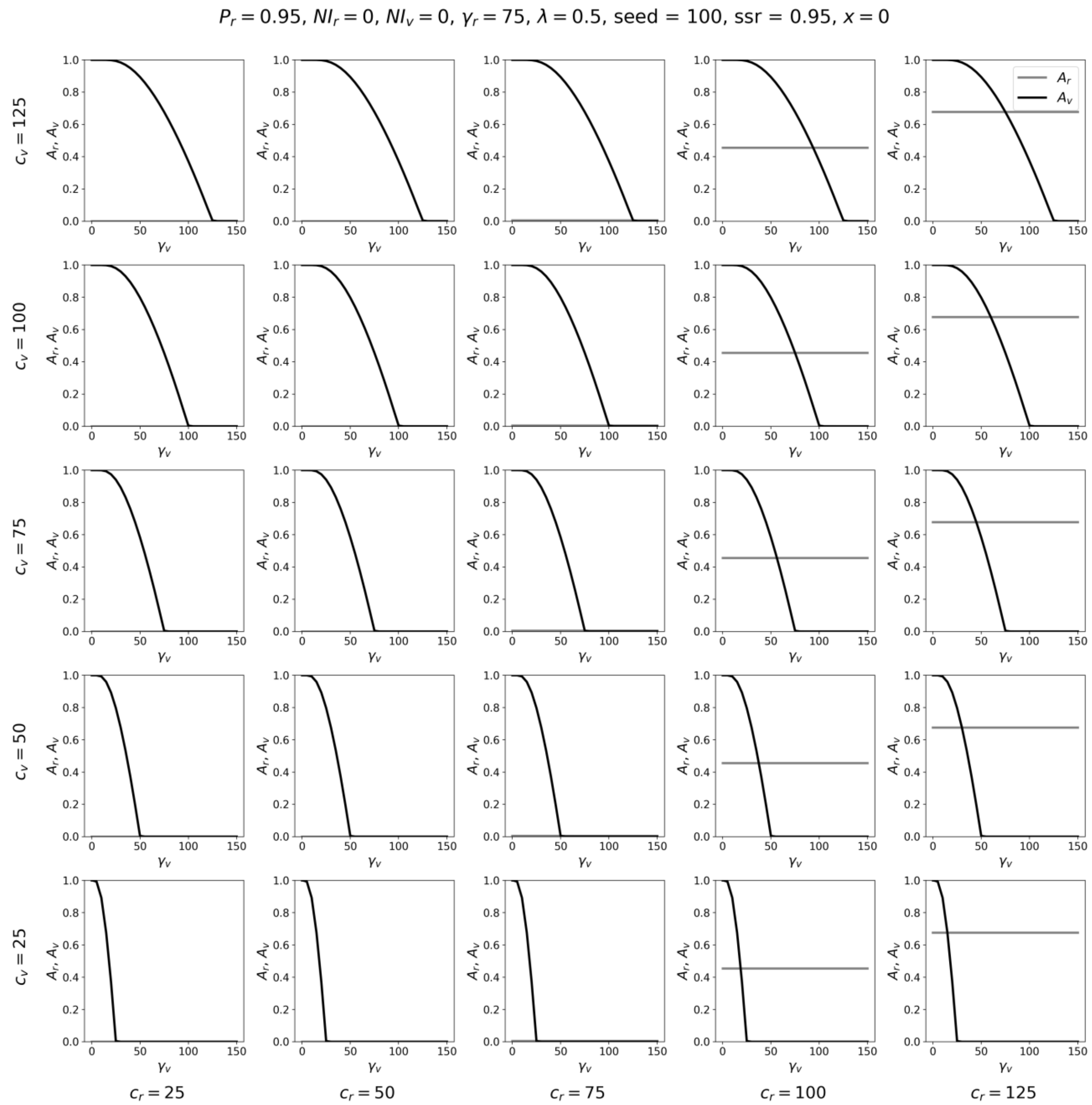
$P_r = 0.95$, $NI_r = 0$, $NI_v = 0$, $\gamma_r = 75$, $\lambda = 0.5$, seed = 100, ssr = 0.95, $x = 0$
$c_v = 125$
$c_v = 100$
$c_v = 75$
$c_v = 50$
$c_v = 25$
$c_r = 25$
$c_r = 50$
$c_r = 75$
$c_r = 100$
$c_r = 125$
A_r
A_v
A_r, A_v
γ_v

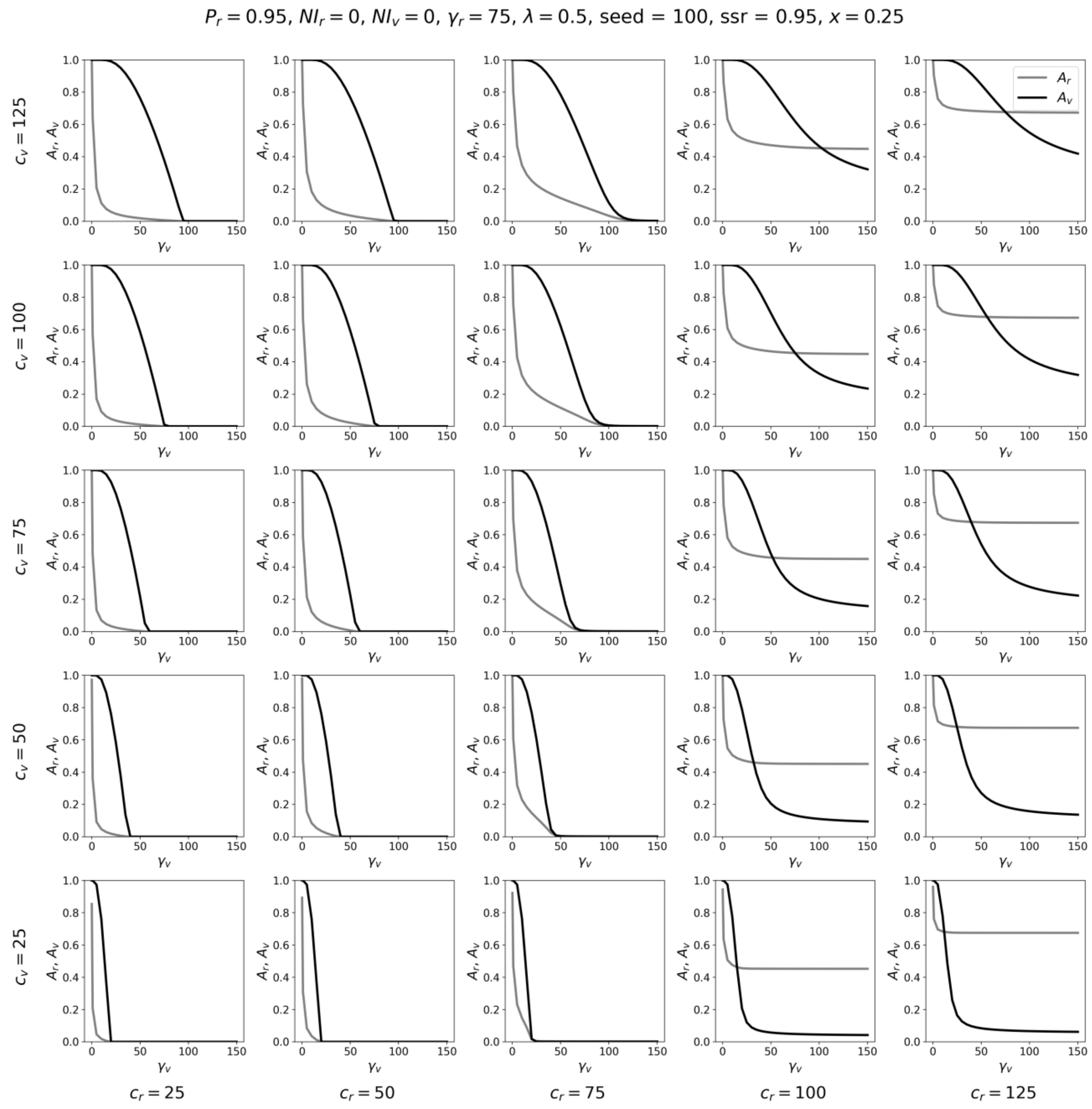
$P_r = 0.95$, $NI_r = 0$, $NI_v = 0$, $\gamma_r = 75$, $\lambda = 0.5$, seed = 100, ssr = 0.95, $x = 0.25$
$c_v = 125$
$c_v = 100$
$c_v = 75$
$c_v = 50$
$c_v = 25$
$c_r = 25$
$c_r = 50$
$c_r = 75$
$c_r = 100$
$c_r = 125$
A_r, A_v
γ_v
A_r
A_v

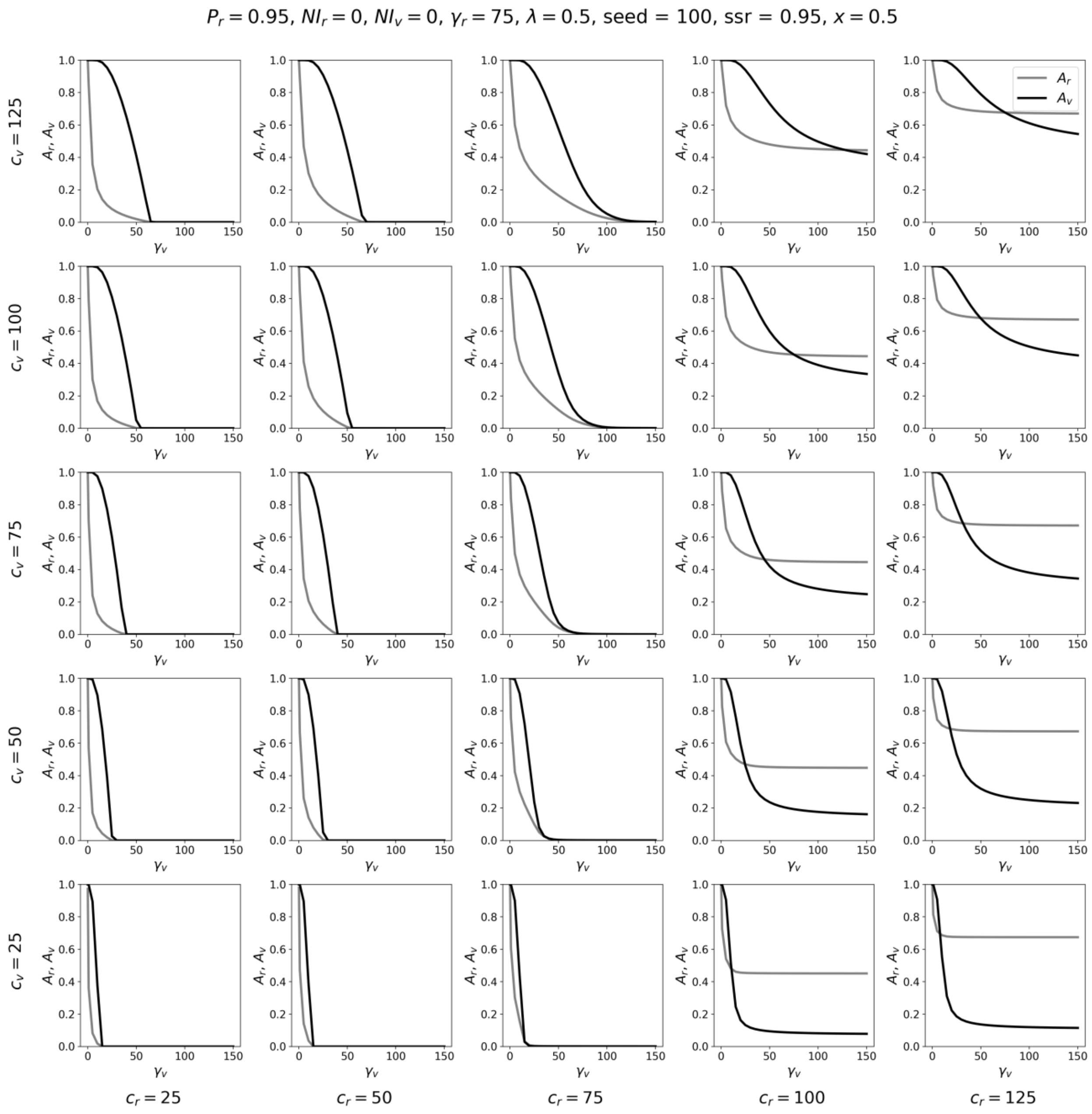

$P_r = 0.95$, $NI_r = 0$, $NI_v = 0$, $\gamma_r = 75$, $\lambda = 0.5$, seed = 100, ssr = 0.95, $x = 0.5$
$c_v = 125$
$c_v = 100$
$c_v = 75$
$c_v = 50$
$c_v = 25$
$c_r = 25$
$c_r = 50$
$c_r = 75$
$c_r = 100$
$c_r = 125$
A_r, A_v
γ_v
A_r
A_v

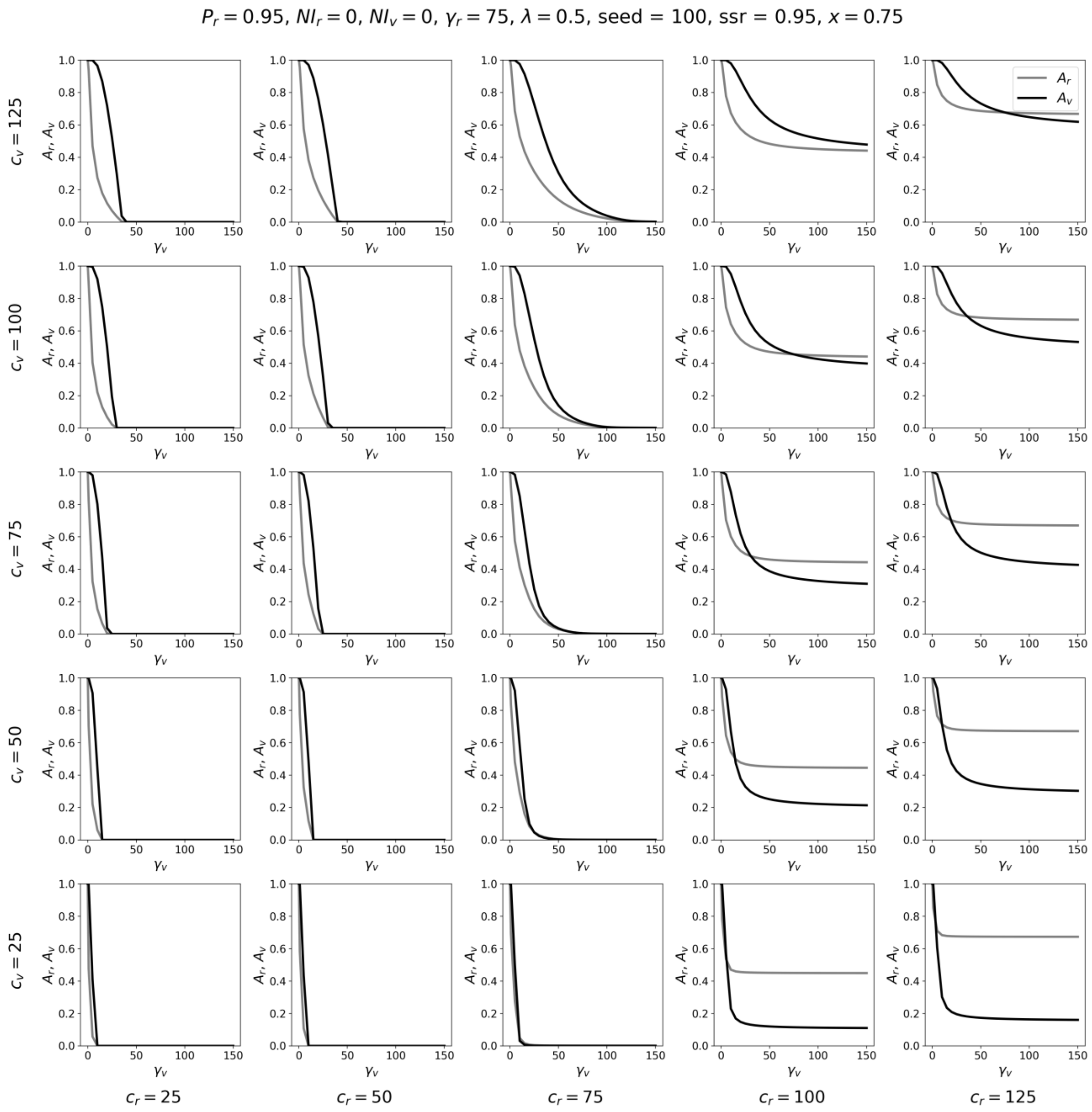

$P_r = 0.95$, $NI_r = 0$, $NI_v = 0$, $\gamma_r = 75$, $\lambda = 0.5$, seed = 100, ssr = 0.95, $x = 0.75$
A_r
A_v
$c_v = 125$
$c_v = 100$
$c_v = 75$
$c_v = 50$
$c_v = 25$
A_r, A_v
γ_v
$c_r = 25$
$c_r = 50$
$c_r = 75$
$c_r = 100$
$c_r = 125$

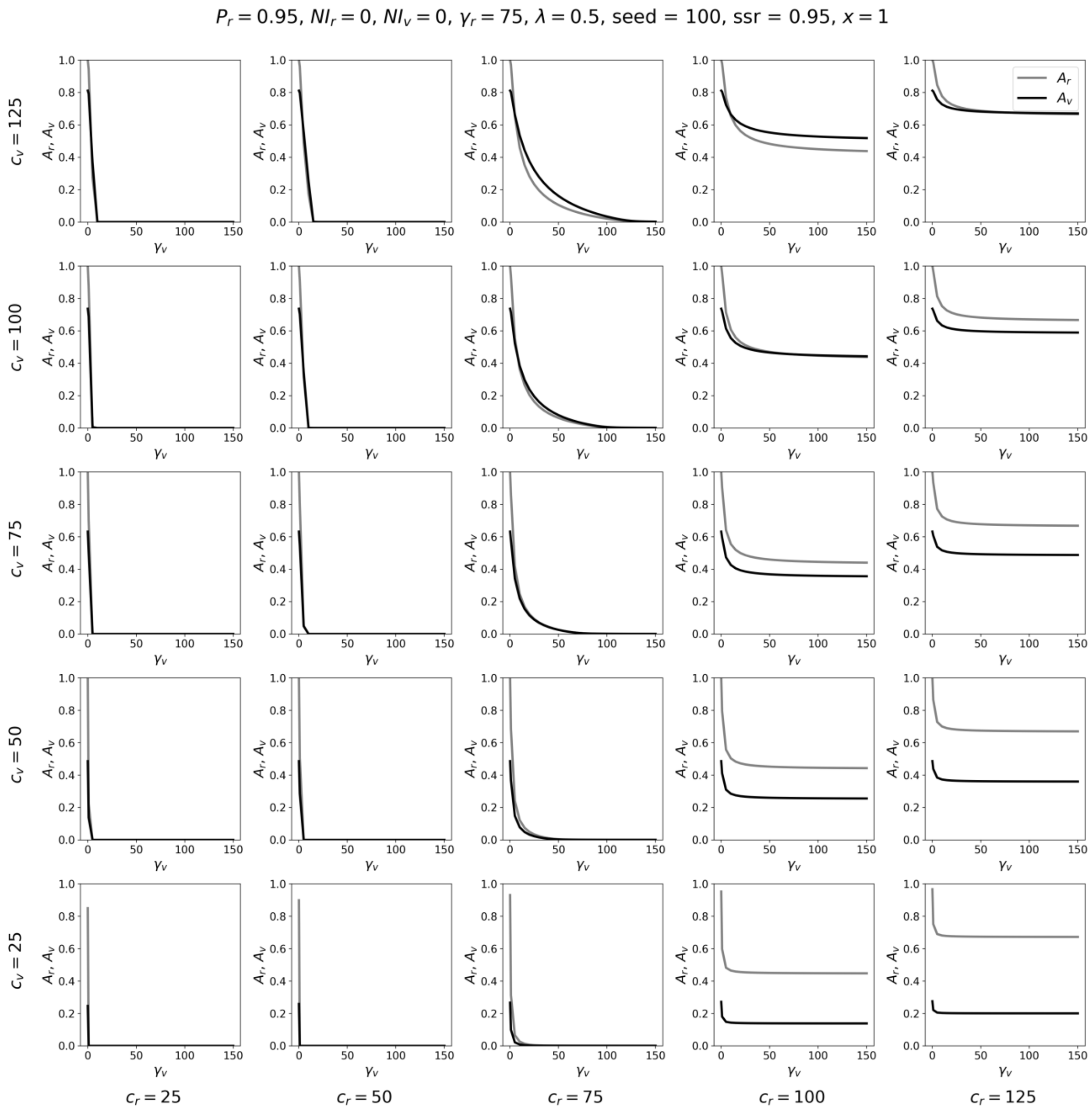

$P_r = 0.95$, $NI_r = 0$, $NI_v = 0$, $\gamma_r = 75$, $\lambda = 0.5$, seed = 100, ssr = 0.95, $x = 1$
A_r
A_v
$c_v = 125$
$c_v = 100$
$c_v = 75$
$c_v = 50$
$c_v = 25$
A_r, A_v
γ_v
$c_r = 25$
$c_r = 50$
$c_r = 75$
$c_r = 100$
$c_r = 125$

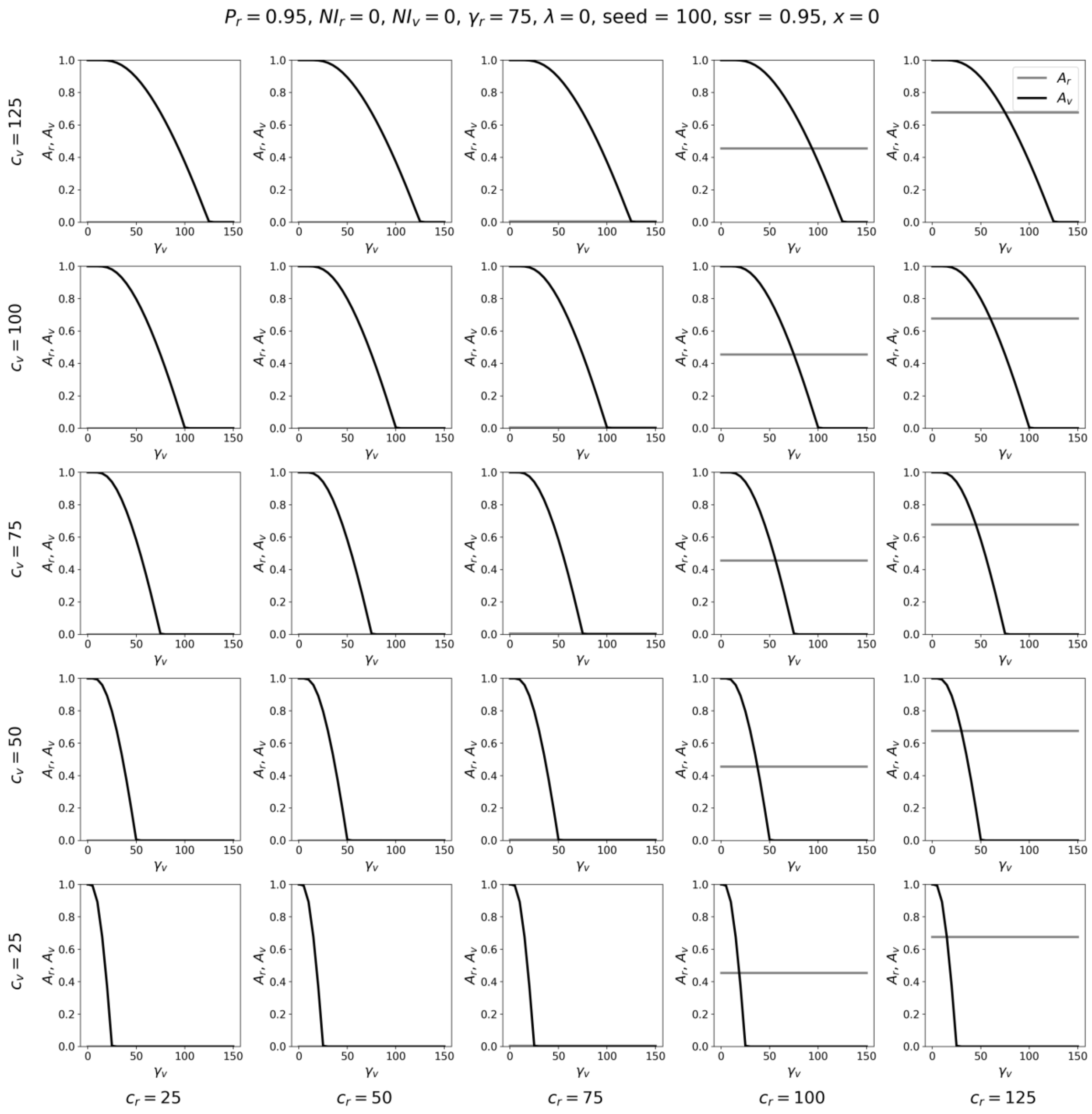

$P_r = 0.95$, $NI_r = 0$, $NI_v = 0$, $\gamma_r = 75$, $\lambda = 0$, seed = 100, ssr = 0.95, $x = 0$
$c_v = 125$
$c_v = 100$
$c_v = 75$
$c_v = 50$
$c_v = 25$
$c_r = 25$
$c_r = 50$
$c_r = 75$
$c_r = 100$
$c_r = 125$
A_r, A_v
γ_v
A_r
A_v

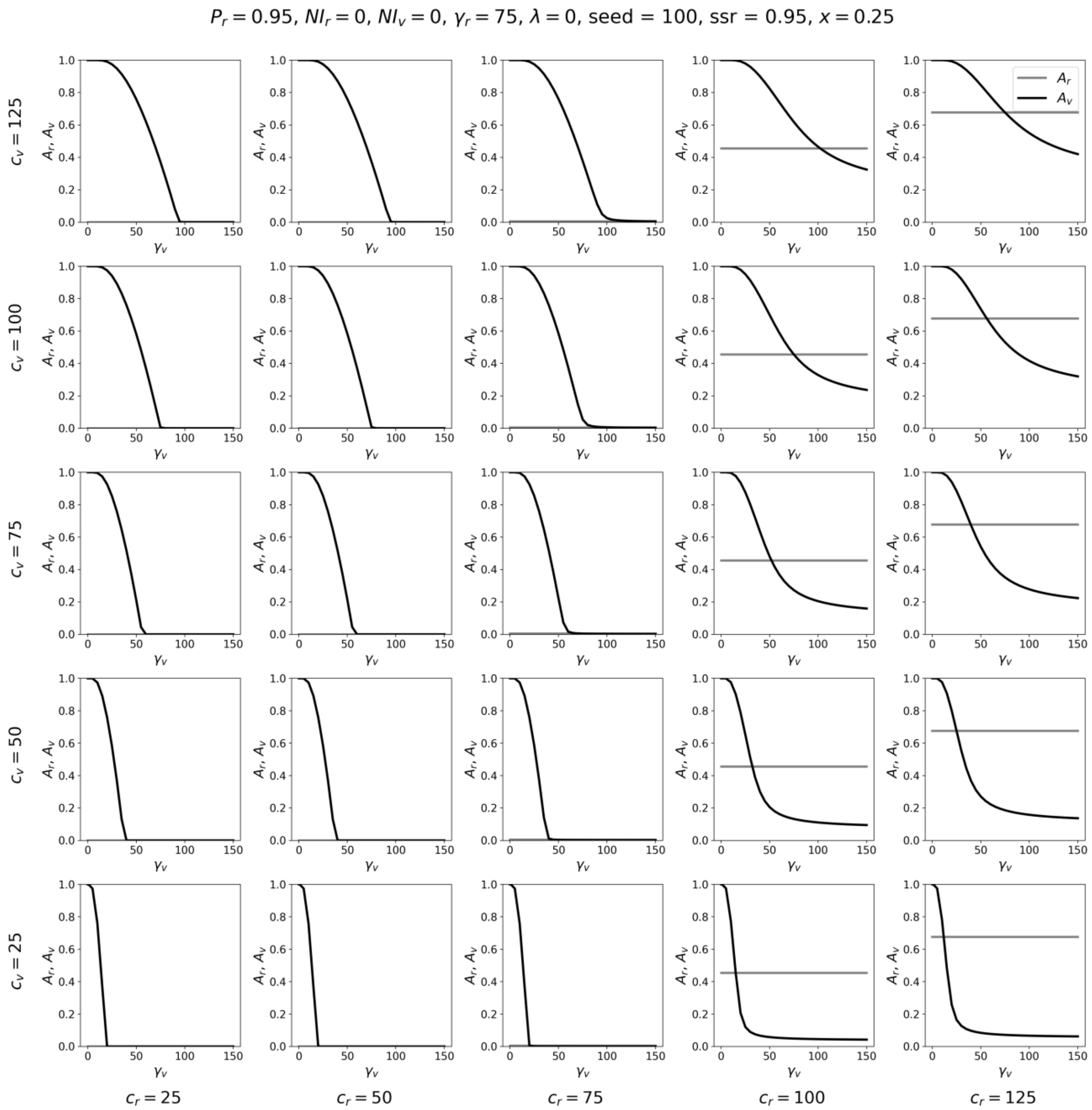

$P_r = 0.95$, $NI_r = 0$, $NI_v = 0$, $\gamma_r = 75$, $\lambda = 0$, seed = 100, ssr = 0.95, $x = 0.25$
$c_v = 125$
$c_v = 100$
$c_v = 75$
$c_v = 50$
$c_v = 25$
$c_r = 25$
$c_r = 50$
$c_r = 75$
$c_r = 100$
$c_r = 125$
A_r
A_v
A_r, A_v
γ_v

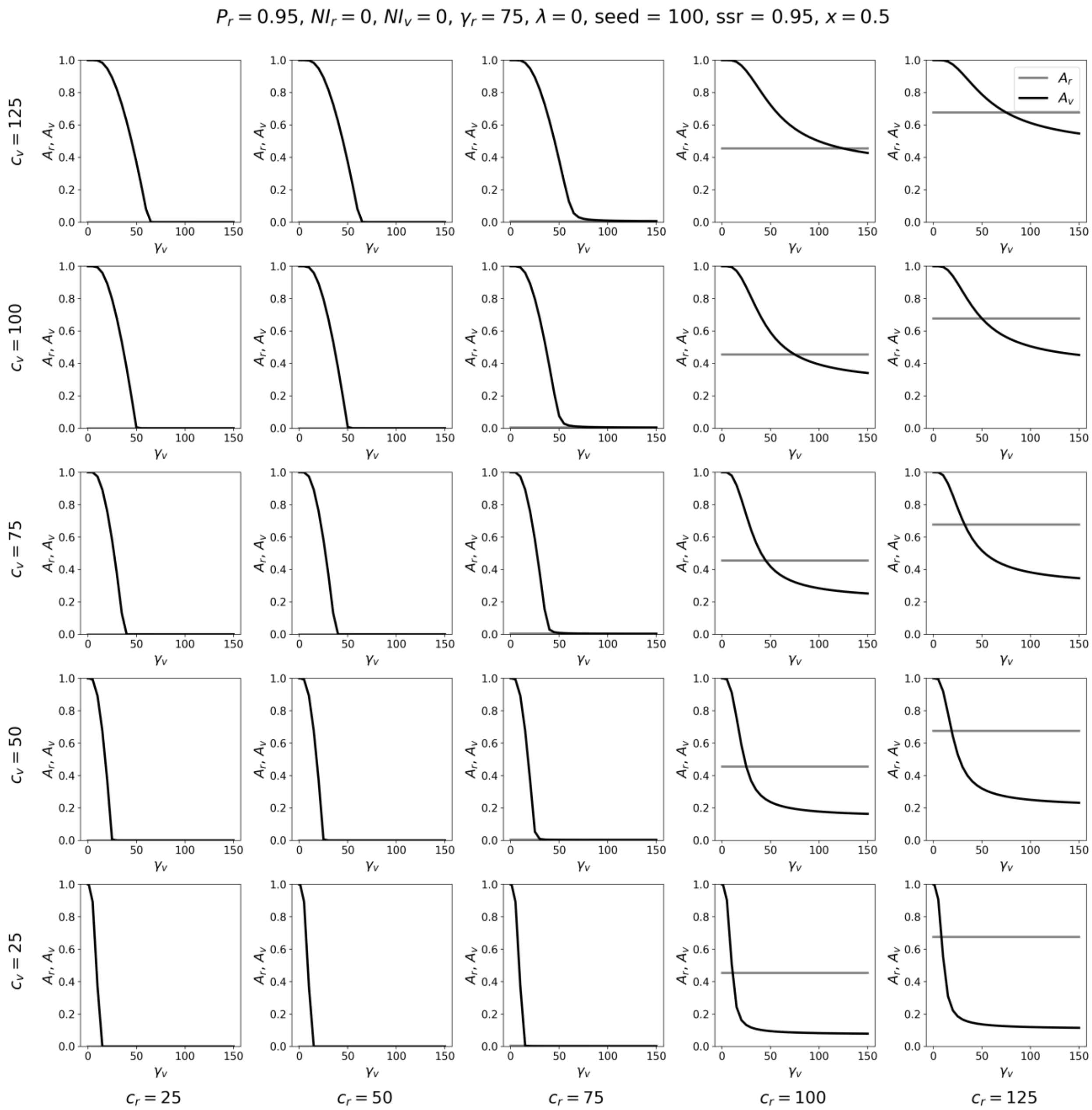

$P_r = 0.95$, $NI_r = 0$, $NI_v = 0$, $\gamma_r = 75$, $\lambda = 0$, seed = 100, ssr = 0.95, $x = 0.5$
$c_v = 125$
$c_v = 100$
$c_v = 75$
$c_v = 50$
$c_v = 25$
A_r, A_v
γ_v
A_r
A_v
$c_r = 25$
$c_r = 50$
$c_r = 75$
$c_r = 100$
$c_r = 125$

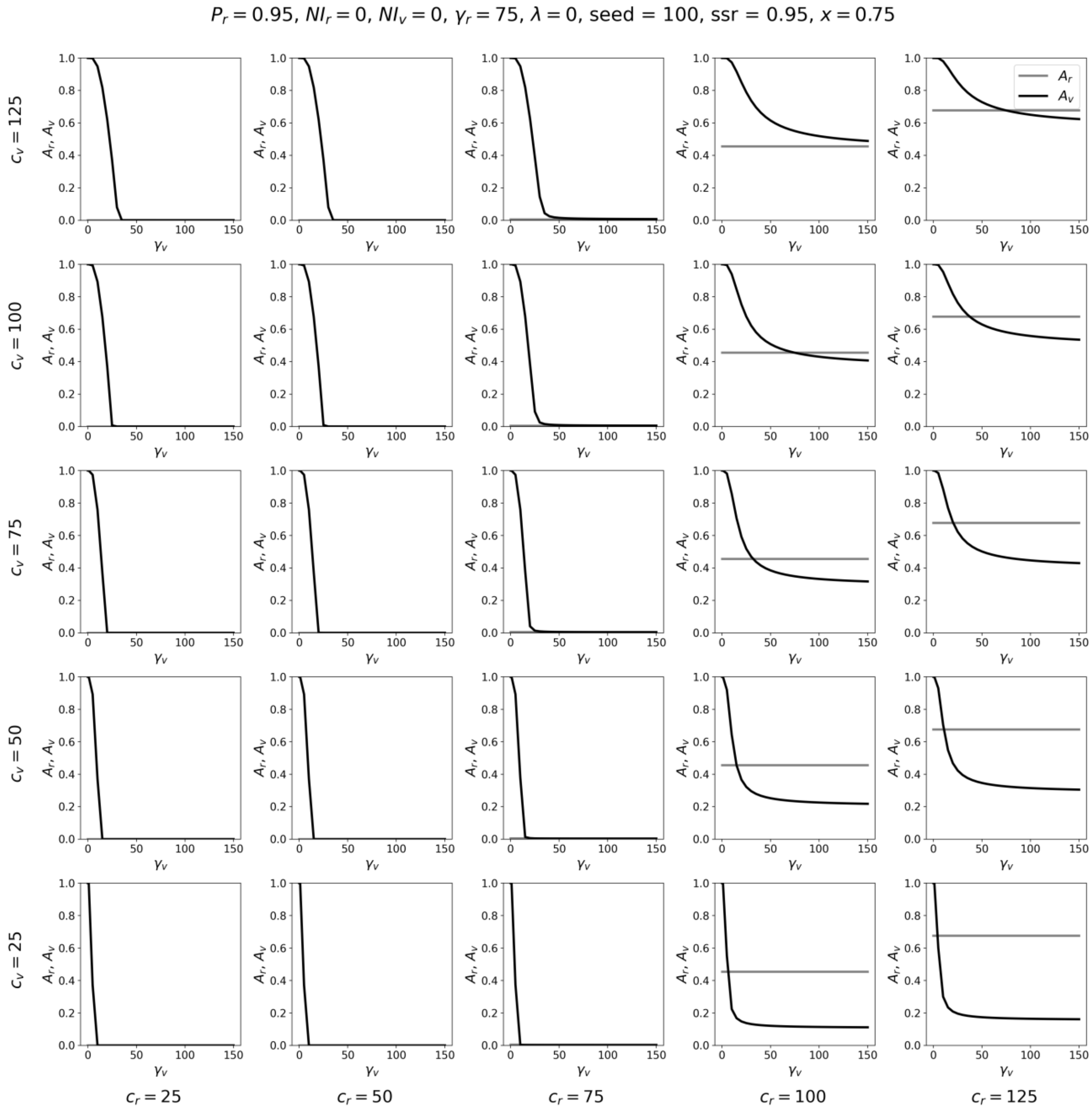
$P_r = 0.95$, $NI_r = 0$, $NI_v = 0$, $\gamma_r = 75$, $\lambda = 0$, seed = 100, ssr = 0.95, $x = 0.75$
$c_v = 125$
$c_v = 100$
$c_v = 75$
$c_v = 50$
$c_v = 25$
$c_r = 25$
$c_r = 50$
$c_r = 75$
$c_r = 100$
$c_r = 125$
A_r, A_v
γ_v
A_r
A_v

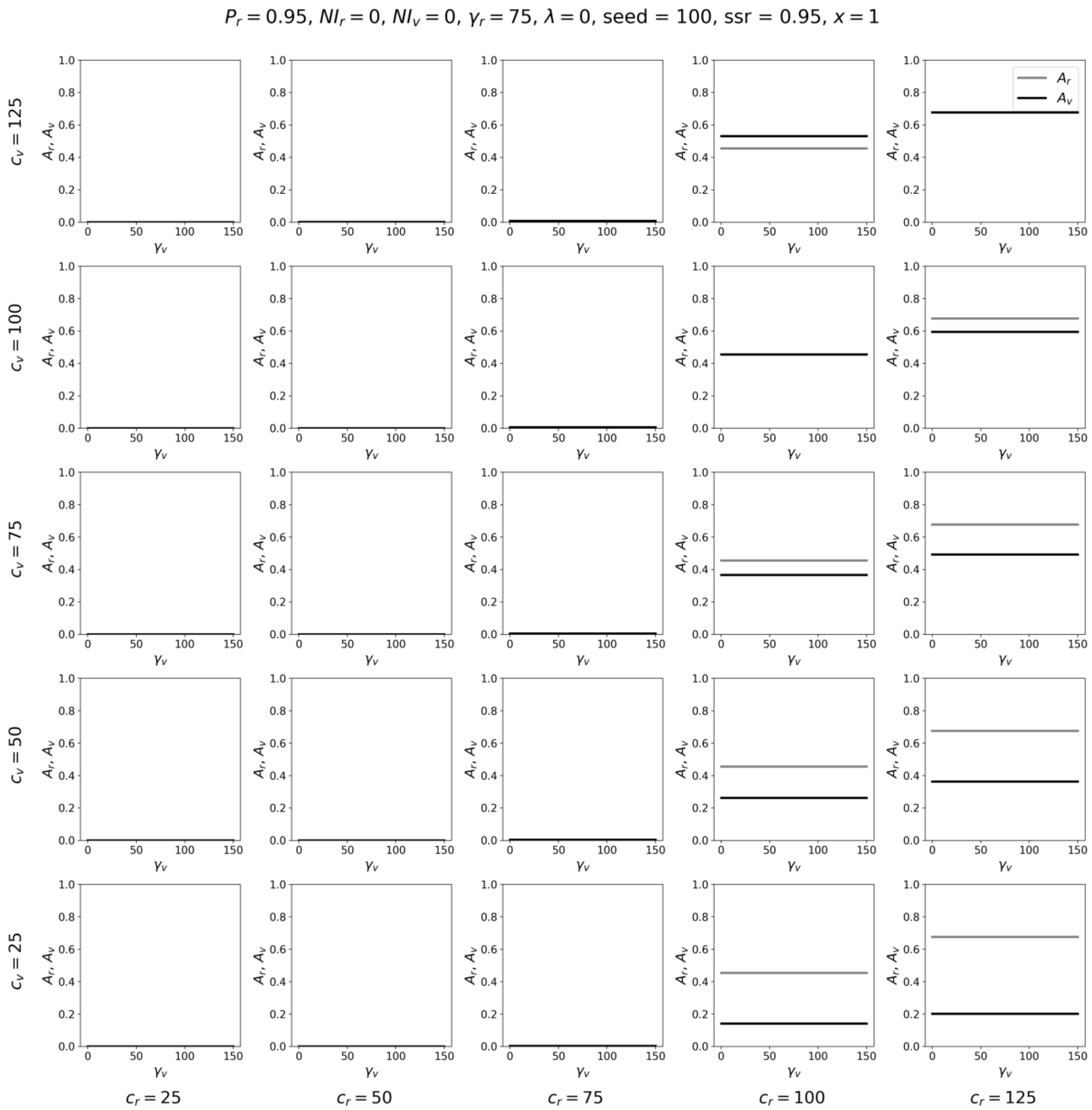
$P_r = 0.95$, $NI_r = 0$, $NI_v = 0$, $\gamma_r = 75$, $\lambda = 0$, seed = 100, ssr = 0.95, $x = 1$
A_r
A_v
$c_v = 125$
$c_v = 100$
$c_v = 75$
$c_v = 50$
$c_v = 25$
A_r, A_v
γ_v
$c_r = 25$
$c_r = 50$
$c_r = 75$
$c_r = 100$
$c_r = 125$

A.4: Epidemic curves for different values of x *and* γ_v*, for* $\lambda = 1$

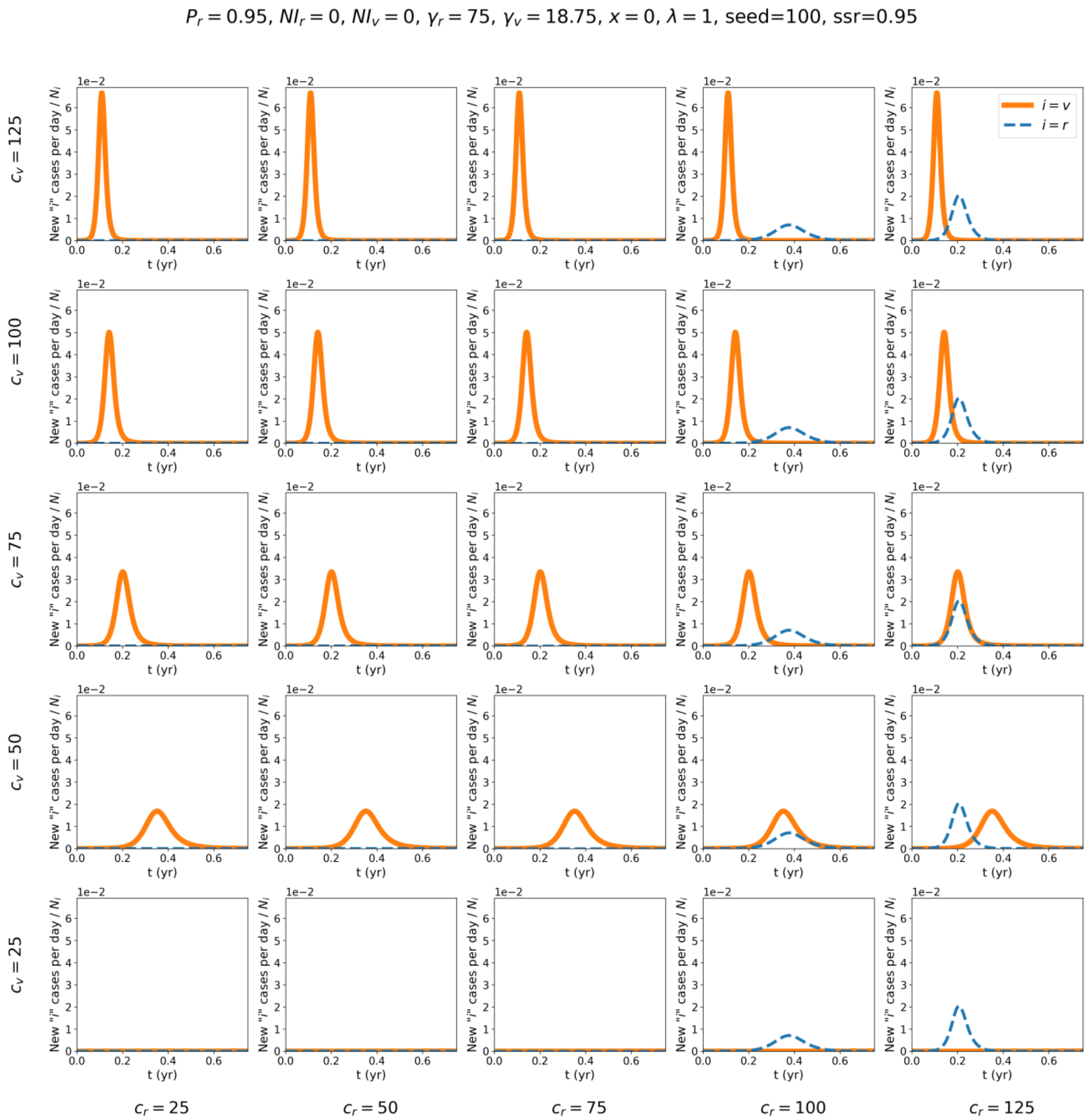
$P_r = 0.95$, $NI_r = 0$, $NI_v = 0$, $\gamma_r = 75$, $\gamma_v = 18.75$, $x = 0$, $\lambda = 1$, seed=100, ssr=0.95
$c_v = 125$
$c_v = 100$
$c_v = 75$
$c_v = 50$
$c_v = 25$
$c_r = 25$
$c_r = 50$
$c_r = 75$
$c_r = 100$
$c_r = 125$
New "i" cases per day / N_i
t (yr)
1e−2
$i = v$
$i = r$

$P_r = 0.95$, $NI_r = 0$, $NI_v = 0$, $\gamma_r = 75$, $\gamma_v = 18.75$, $x = 0.25$, $\lambda = 1$, seed=100, ssr=0.95

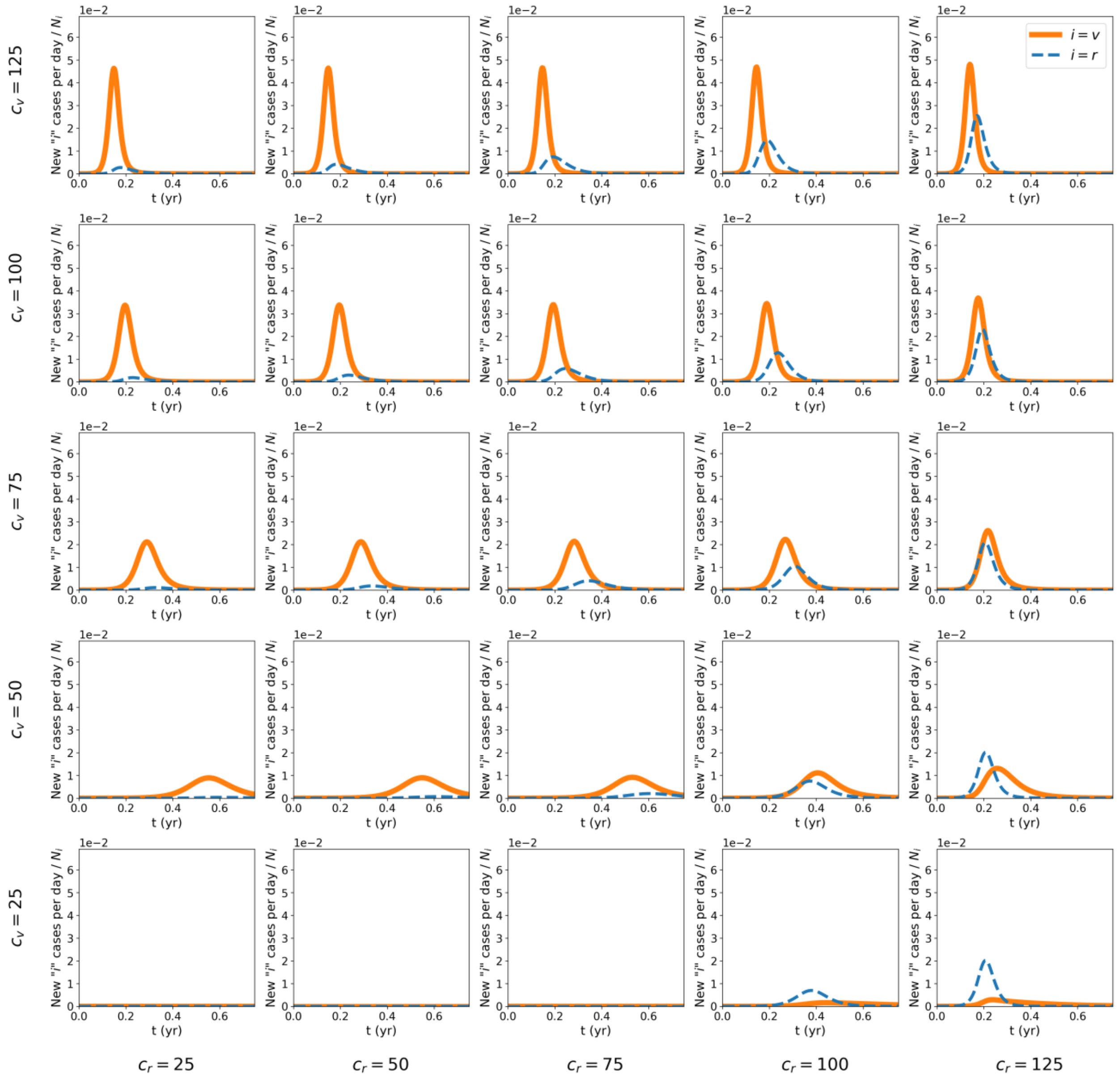

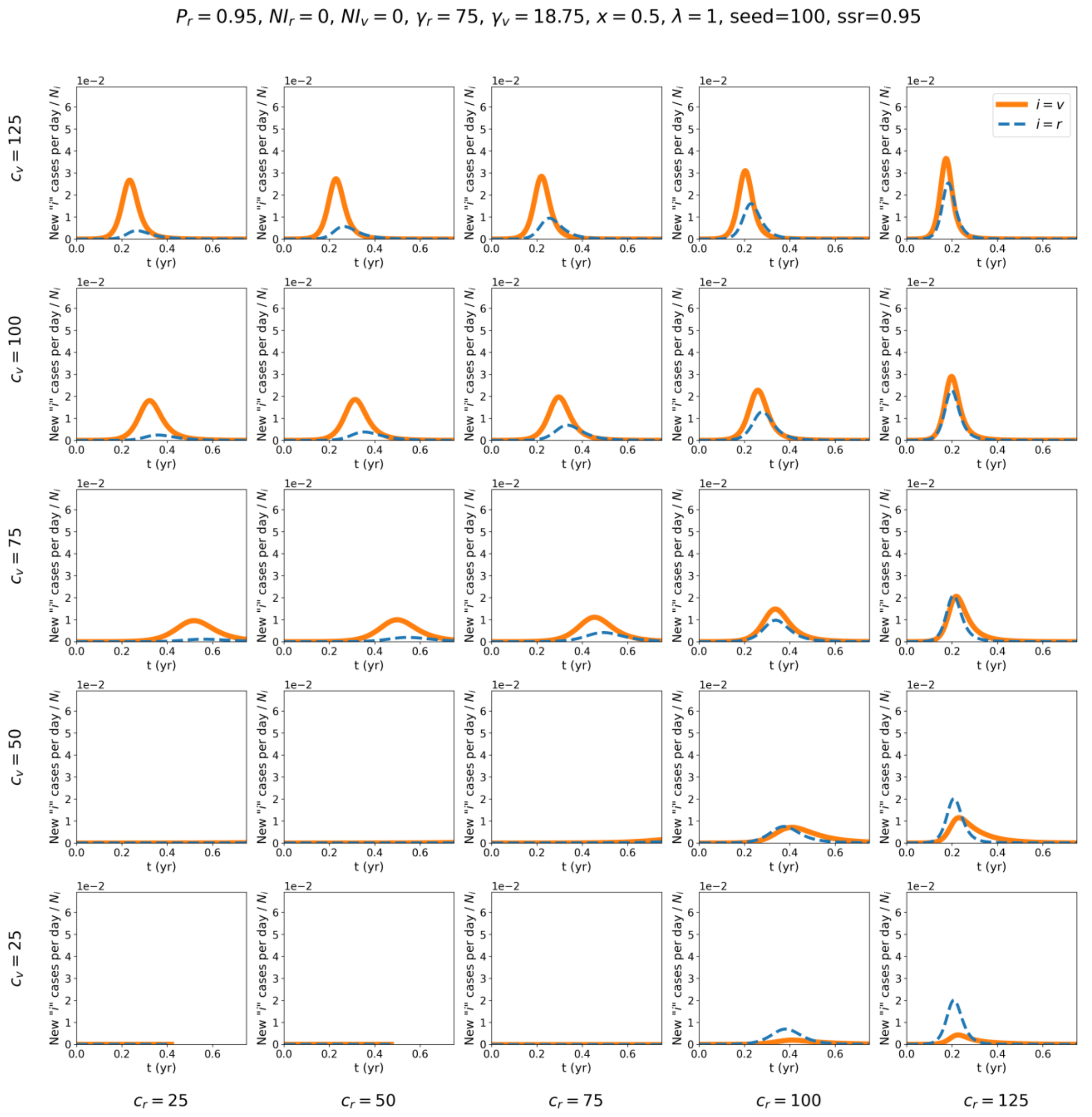
$P_r = 0.95$, $NI_r = 0$, $NI_v = 0$, $\gamma_r = 75$, $\gamma_v = 18.75$, $x = 0.5$, $\lambda = 1$, seed=100, ssr=0.95
$c_v = 125$
$c_v = 100$
$c_v = 75$
$c_v = 50$
$c_v = 25$
$c_r = 25$
$c_r = 50$
$c_r = 75$
$c_r = 100$
$c_r = 125$
$i = v$
$i = r$
New "i" cases per day / N_i
t (yr)
1e−2

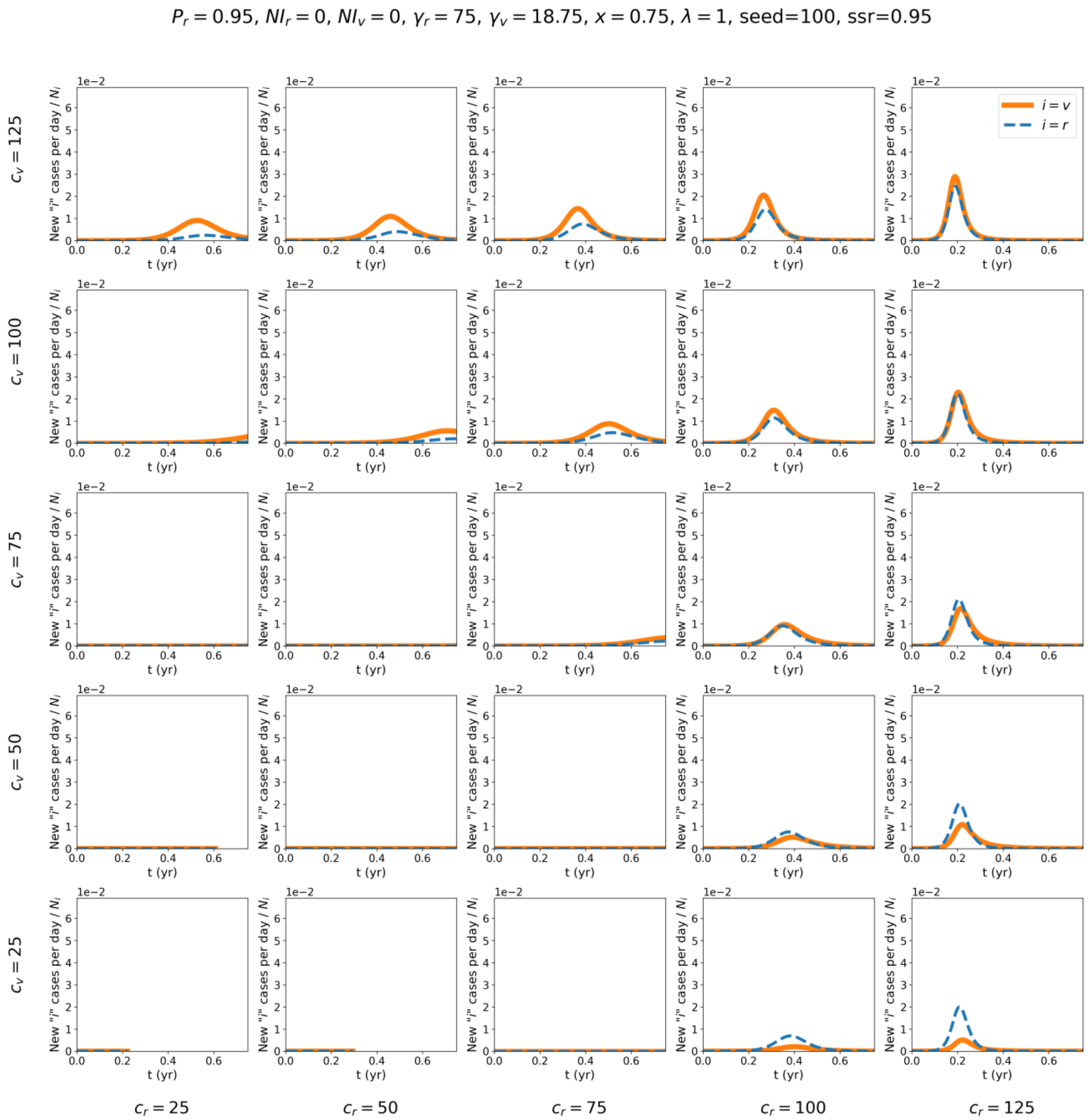
$P_r = 0.95$, $NI_r = 0$, $NI_v = 0$, $\gamma_r = 75$, $\gamma_v = 18.75$, $x = 0.75$, $\lambda = 1$, seed=100, ssr=0.95
$c_v = 125$
$c_v = 100$
$c_v = 75$
$c_v = 50$
$c_v = 25$
$c_r = 25$
$c_r = 50$
$c_r = 75$
$c_r = 100$
$c_r = 125$
New "i" cases per day / N_i
t (yr)
1e−2
$i = v$
$i = r$

$P_r = 0.95$, $NI_r = 0$, $NI_v = 0$, $\gamma_r = 75$, $\gamma_v = 18.75$, $x = 1$, $\lambda = 1$, seed=100, ssr=0.95

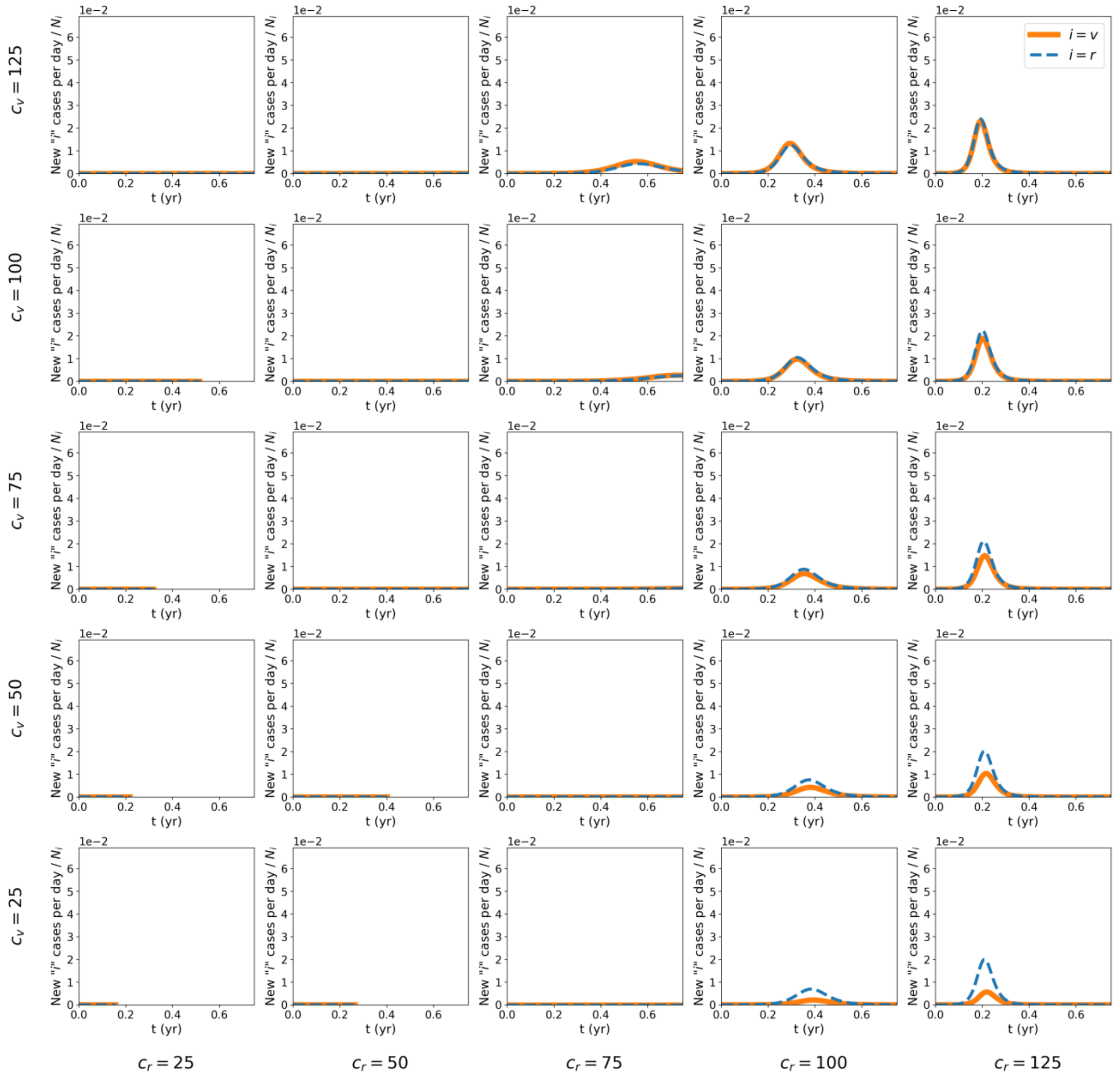

$P_r = 0.95$, $NI_r = 0$, $NI_v = 0$, $\gamma_r = 75$, $\gamma_v = 37.5$, $x = 0$, $\lambda = 1$, seed=100, ssr=0.95

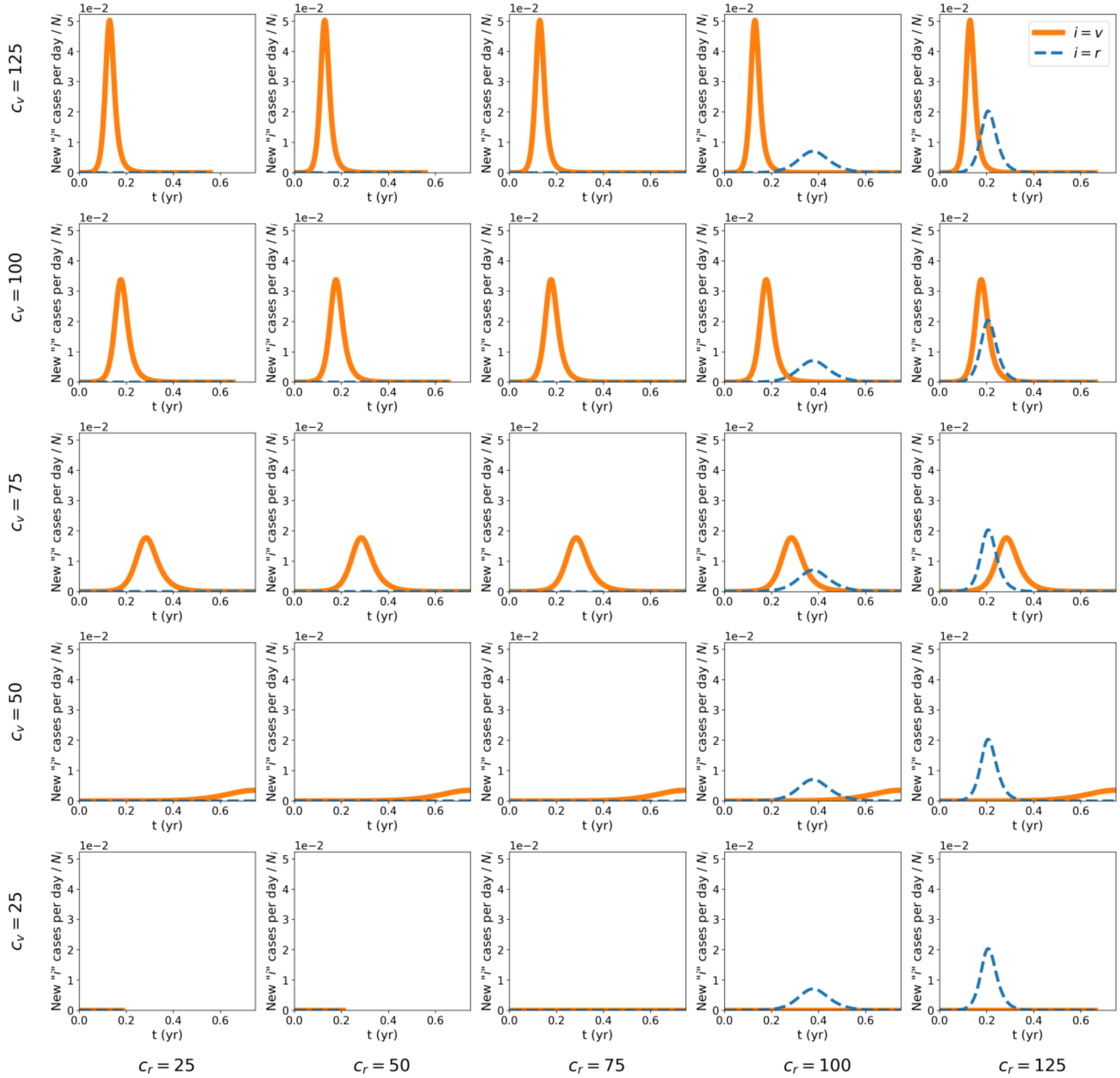

$P_r = 0.95$, $NI_r = 0$, $NI_v = 0$, $\gamma_r = 75$, $\gamma_v = 37.5$, $x = 0.25$, $\lambda = 1$, seed=100, ssr=0.95

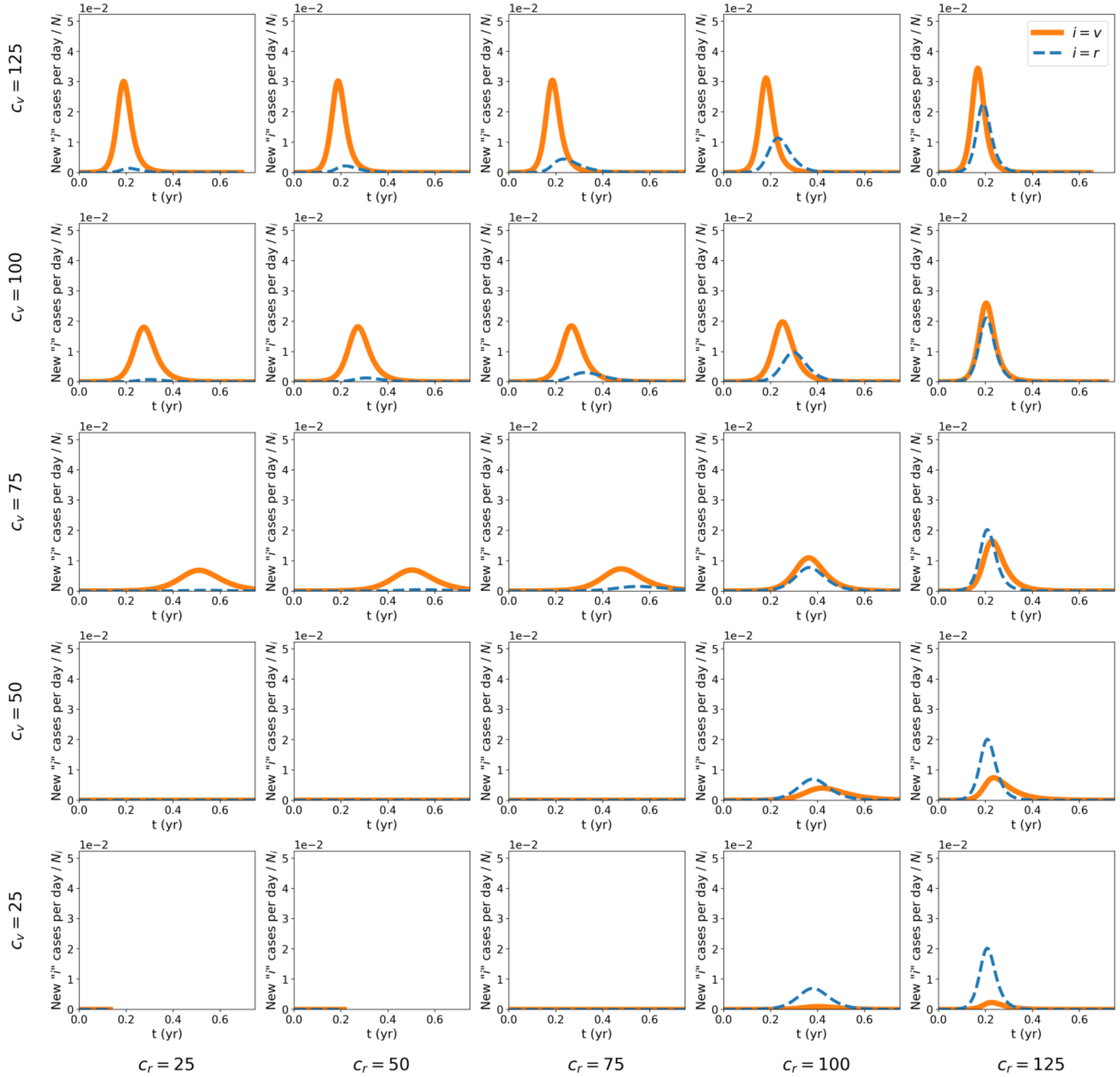

$P_r = 0.95$, $NI_r = 0$, $NI_v = 0$, $\gamma_r = 75$, $\gamma_v = 37.5$, $x = 0.5$, $\lambda = 1$, seed=100, ssr=0.95

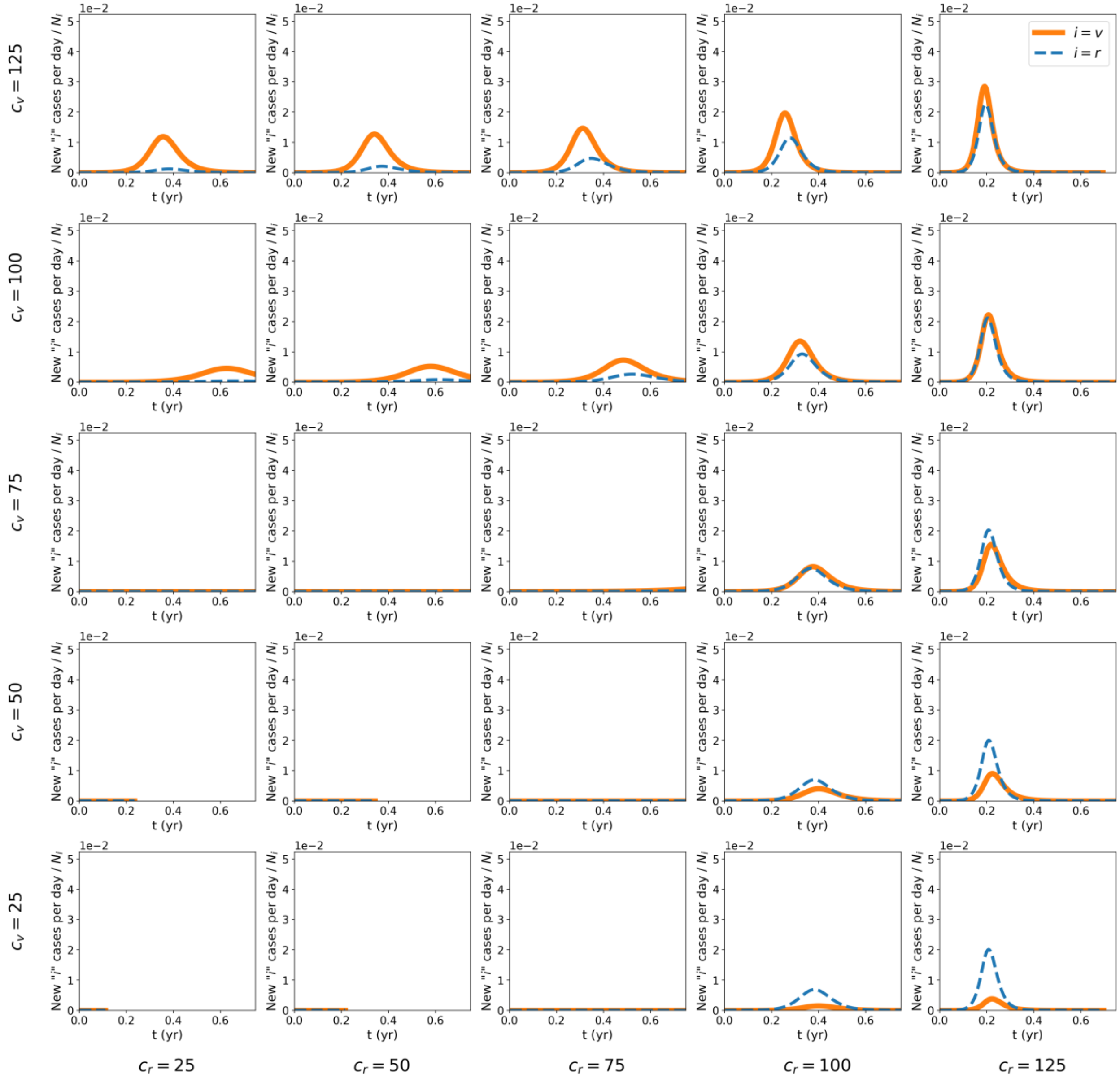

$P_r = 0.95$, $NI_r = 0$, $NI_v = 0$, $\gamma_r = 75$, $\gamma_v = 37.5$, $x = 0.75$, $\lambda = 1$, seed=100, ssr=0.95

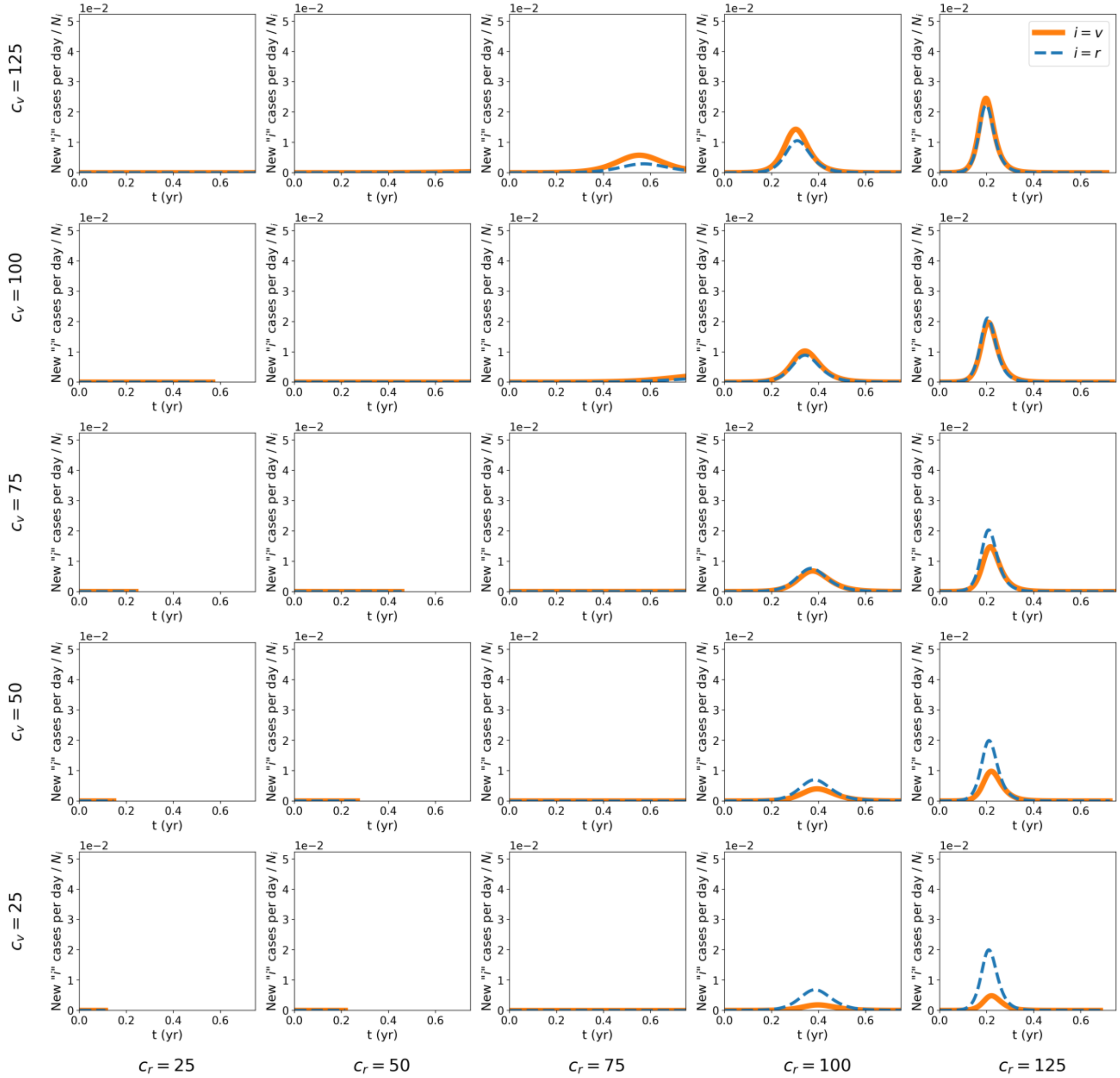

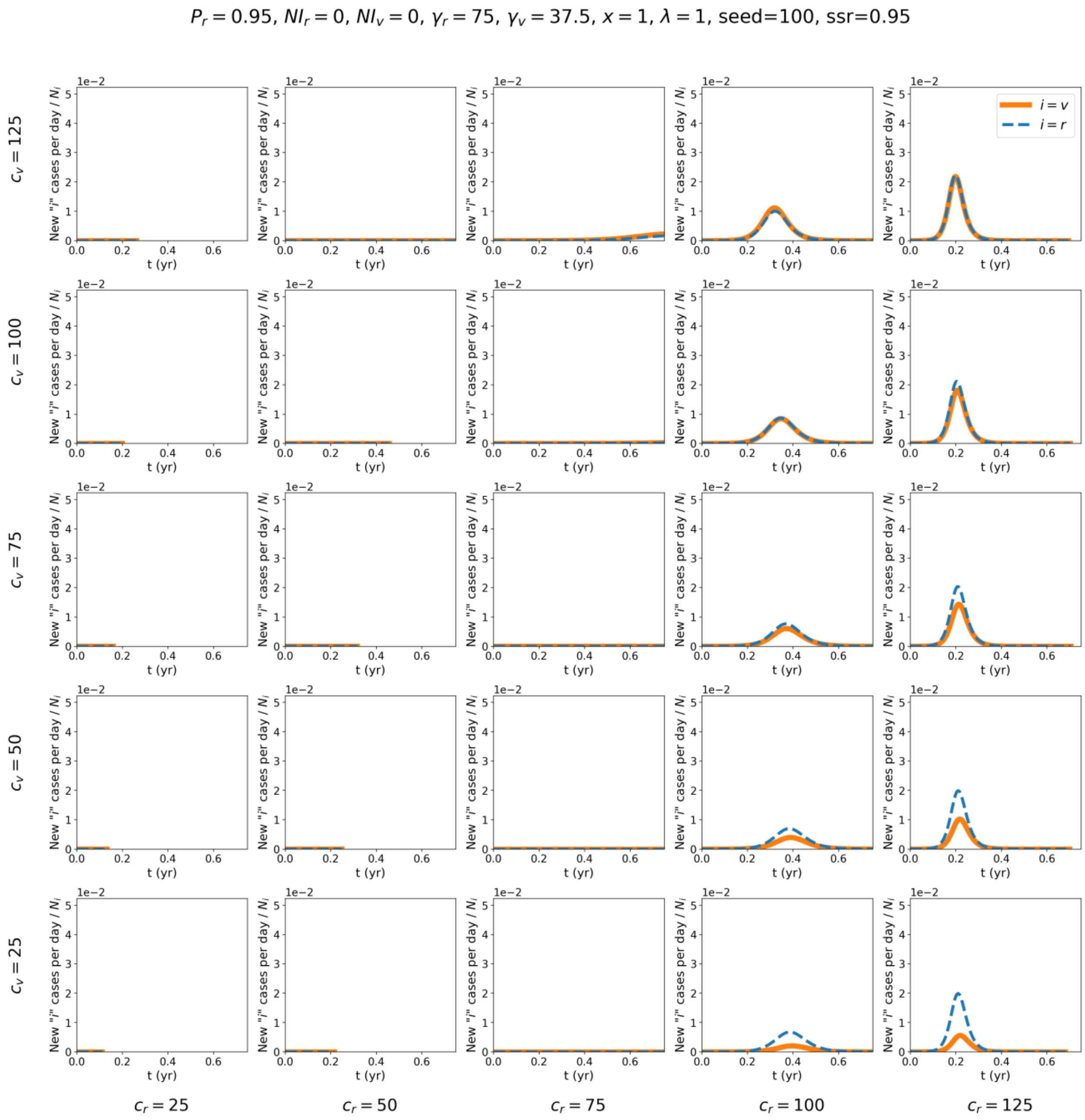
$P_r = 0.95$, $NI_r = 0$, $NI_v = 0$, $\gamma_r = 75$, $\gamma_v = 37.5$, $x = 1$, $\lambda = 1$, seed=100, ssr=0.95
$c_v = 125$
$c_v = 100$
$c_v = 75$
$c_v = 50$
$c_v = 25$
$c_r = 25$
$c_r = 50$
$c_r = 75$
$c_r = 100$
$c_r = 125$
New "i" cases per day / N_i
t (yr)
1e−2
$i = v$
$i = r$

$P_r = 0.95$, $NI_r = 0$, $NI_v = 0$, $\gamma_r = 75$, $\gamma_v = 75$, $x = 0$, $\lambda = 1$, seed=100, ssr=0.95

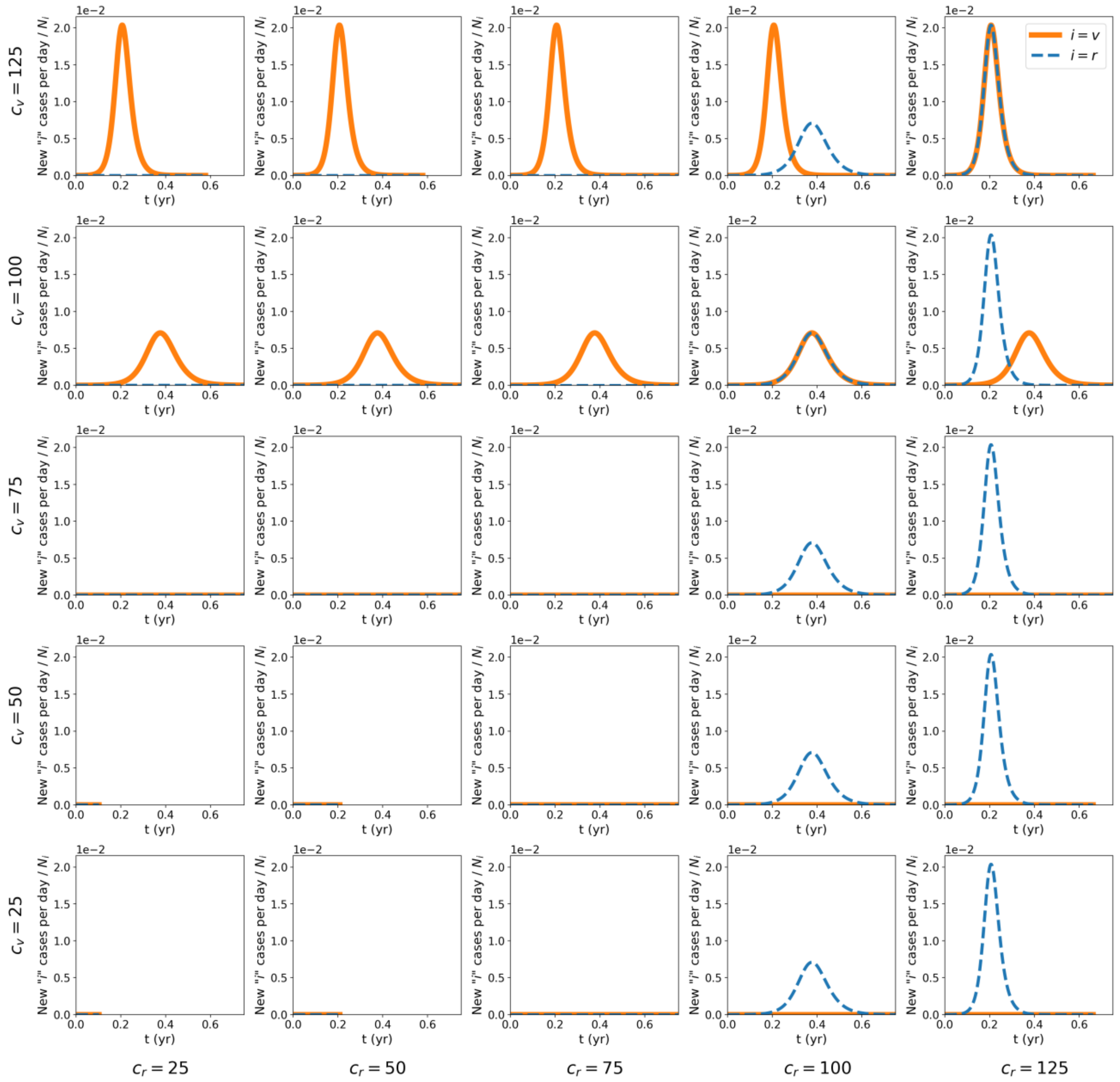

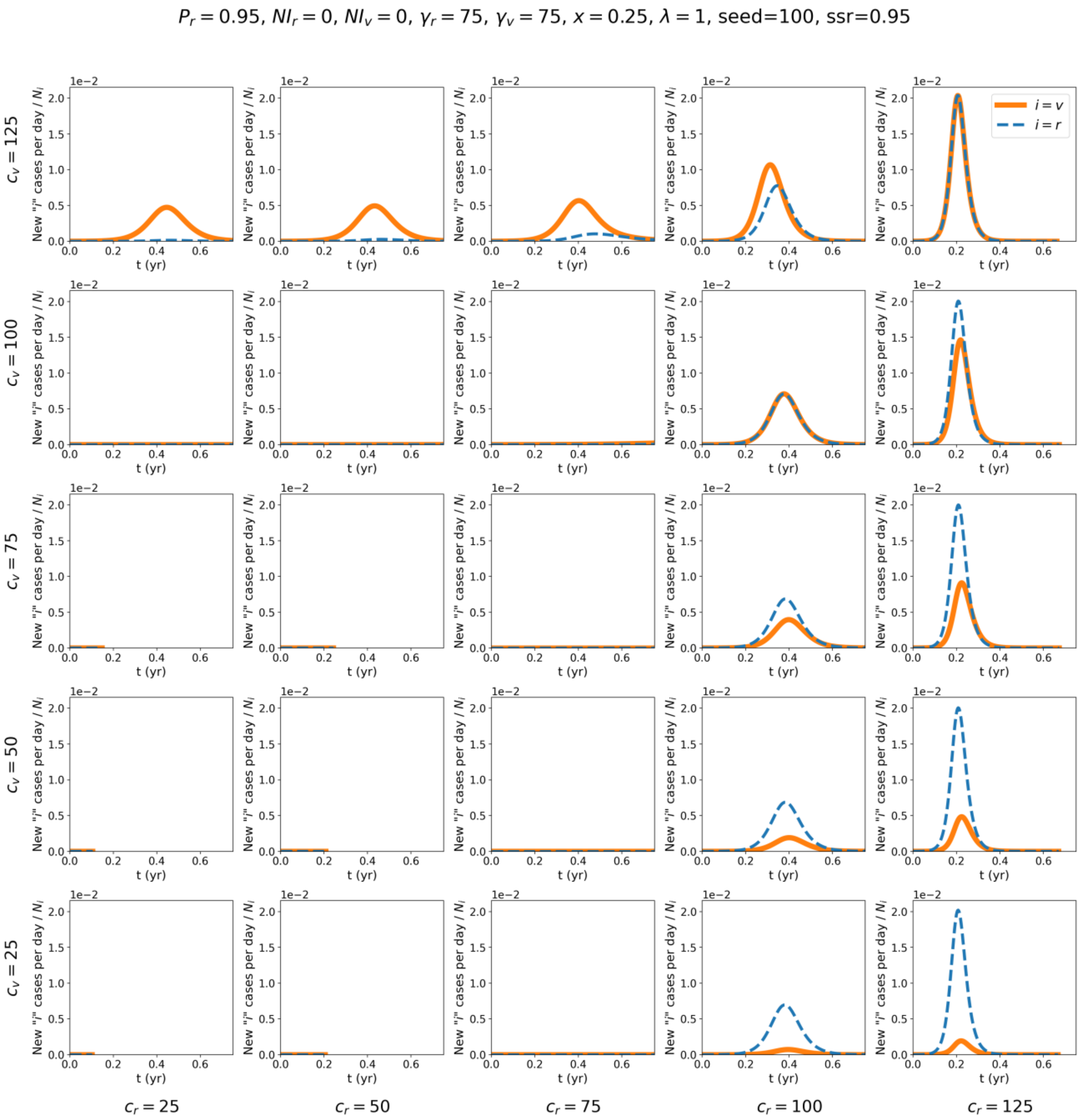

$P_r = 0.95$, $NI_r = 0$, $NI_v = 0$, $\gamma_r = 75$, $\gamma_v = 75$, $x = 0.25$, $\lambda = 1$, seed=100, ssr=0.95
$c_v = 125$
$c_v = 100$
$c_v = 75$
$c_v = 50$
$c_v = 25$
New "i" cases per day / N_i
1e−2
t (yr)
$i = v$
$i = r$
$c_r = 25$
$c_r = 50$
$c_r = 75$
$c_r = 100$
$c_r = 125$

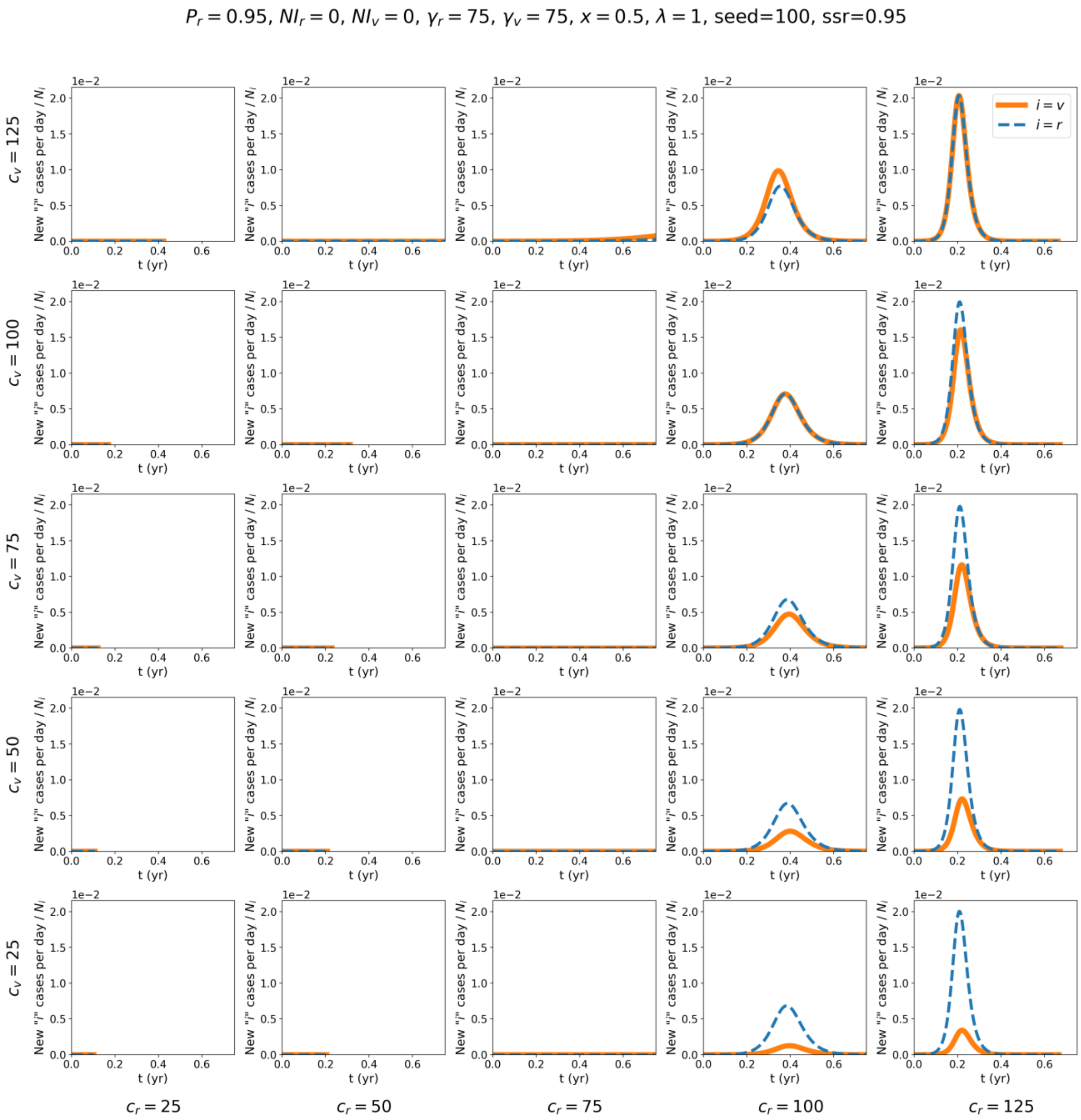
$P_r = 0.95$, $NI_r = 0$, $NI_v = 0$, $\gamma_r = 75$, $\gamma_v = 75$, $x = 0.5$, $\lambda = 1$, seed=100, ssr=0.95
$c_v = 125$
$c_v = 100$
$c_v = 75$
$c_v = 50$
$c_v = 25$
$c_r = 25$
$c_r = 50$
$c_r = 75$
$c_r = 100$
$c_r = 125$
New "i" cases per day / N_i
t (yr)
1e−2
$i = v$
$i = r$

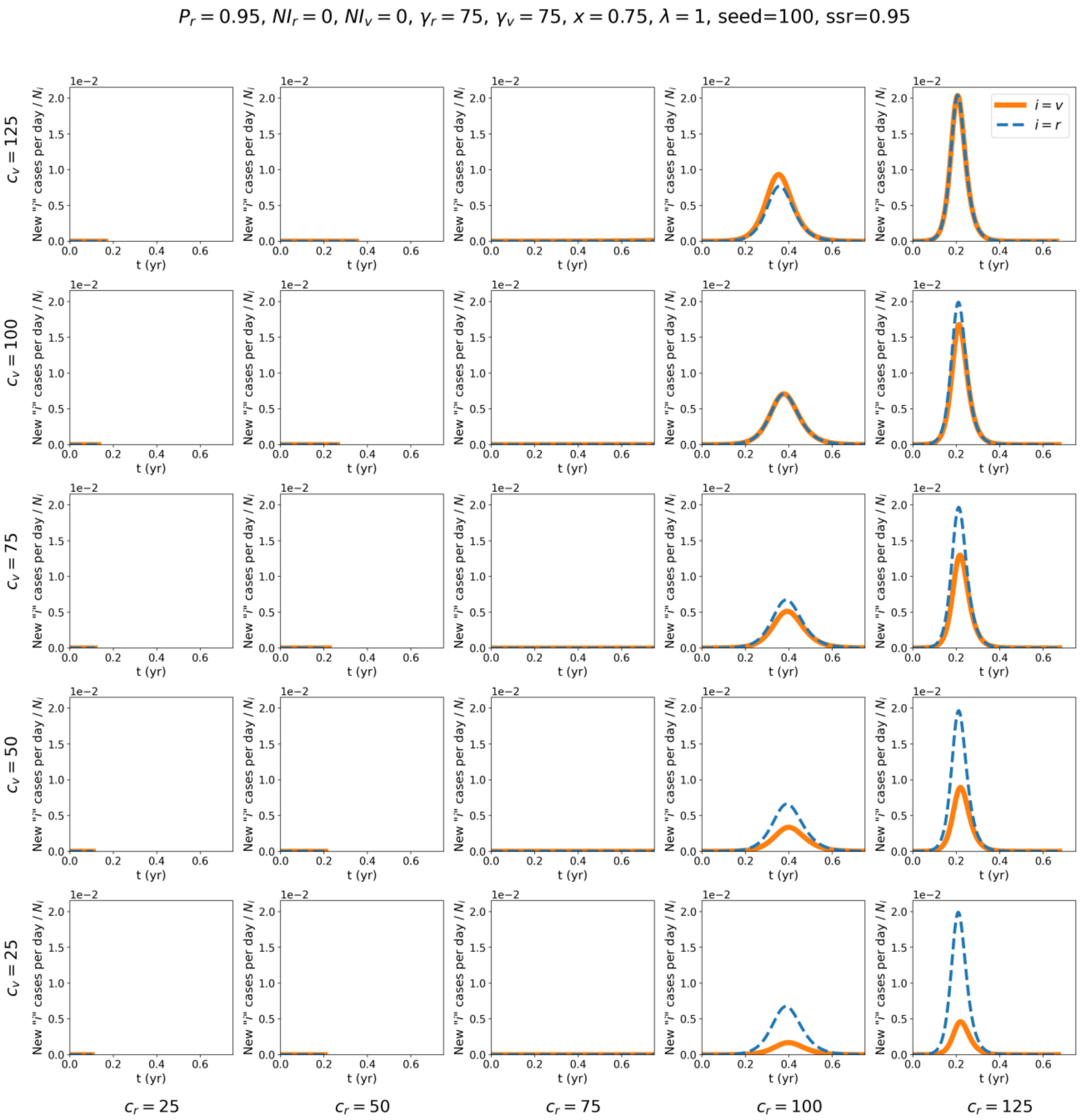
$P_r = 0.95$, $NI_r = 0$, $NI_v = 0$, $\gamma_r = 75$, $\gamma_v = 75$, $x = 0.75$, $\lambda = 1$, seed=100, ssr=0.95
$c_v = 125$
$c_v = 100$
$c_v = 75$
$c_v = 50$
$c_v = 25$
$c_r = 25$
$c_r = 50$
$c_r = 75$
$c_r = 100$
$c_r = 125$
New "i" cases per day / N_i
t (yr)
1e−2
$i = v$
$i = r$

$P_r = 0.95$, $NI_r = 0$, $NI_v = 0$, $\gamma_r = 75$, $\gamma_v = 75$, $x = 1$, $\lambda = 1$, seed=100, ssr=0.95

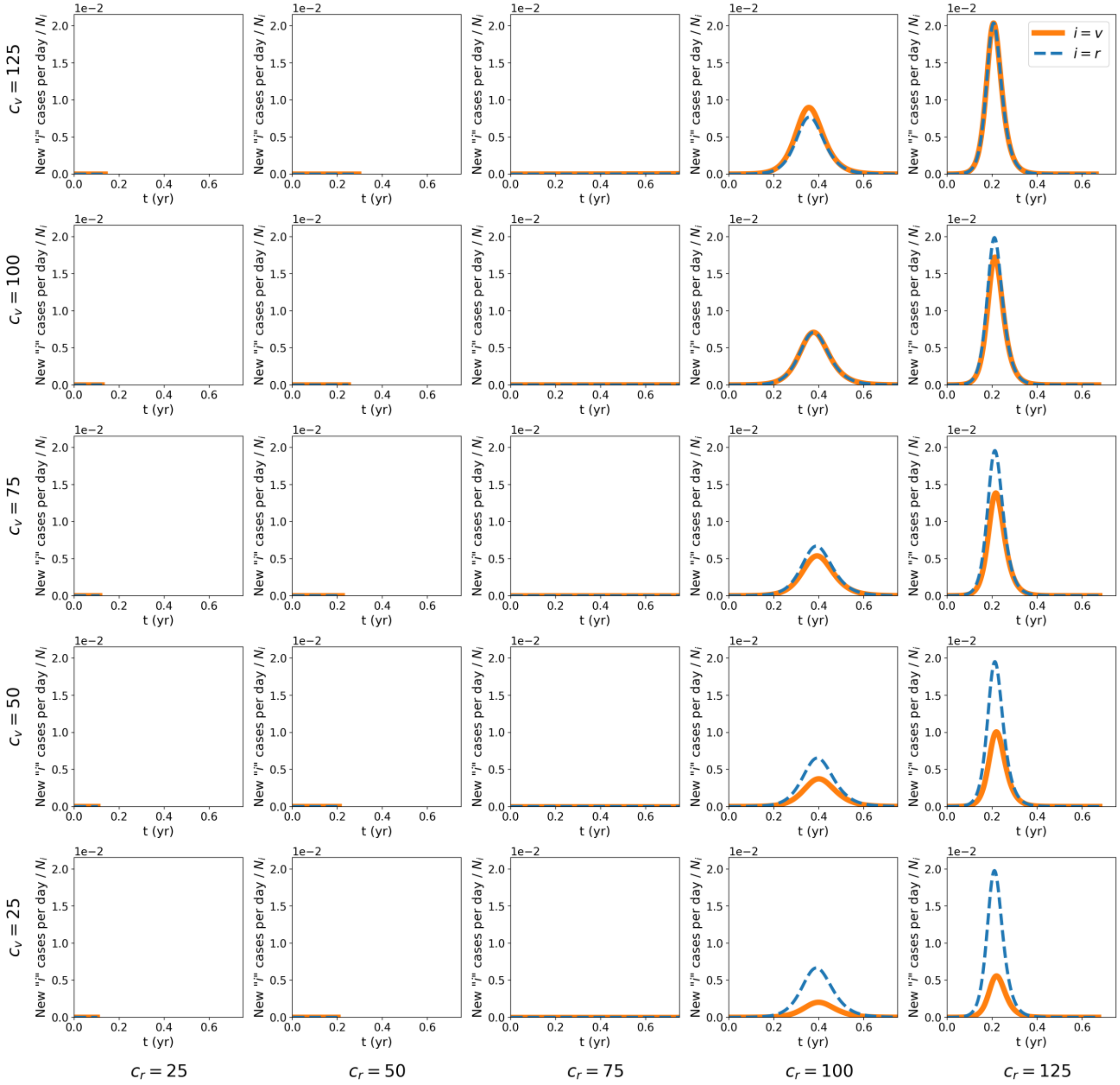

Appendix B: Results for P_r = 0.99, P_r = 0.8 and P_r = 0.6

This Appendix shows attack-rate contour maps for three different values of P_r, for the same values of c_r, c_v, and x used in the main text and elsewhere in the Appendices.

Note that Eqs. 2 and 4 of the main text impose constraints on the c_{ij}. In some of the contour maps shown in Appendix B and Appendix C, the contour lines end abruptly at points in the (c_r, c_v) plane where these constraints are reached.

For example, for $P_r = 0.8$, $x = 1$, and $\gamma_v = 18.75$, the point ($c_r = 20$, $c_v = 100$) is unphysical, because $x = 1$ implies that $c_{vr} = c_v = 100$ (see Eq. 4, main text), and $\lambda = 1$, $P_r = 0.8$, and $c_{vr} = 100$ are such (from Eq. 2, main text) that $c_{rv} = \lambda c_{vr}(1\text{-}P_r)/P_r = 25 > c_r$ which is unphysical, since $c_r = c_{rr} + c_{rv}$ and both $c_{rr} \geq 0$ and $c_{rv} \geq 0$. Accordingly, the contour lines in the contour map for $P_r = 0.8$, $x = 1$, and $\gamma_v = 18.75$ (lower-left panel in the first figure in section B.2, below) end before the unphysical point ($c_r = 20$, $c_v = 100$).

B.1: Attack-rate contour maps for different values of λ and for P_r = 0.99

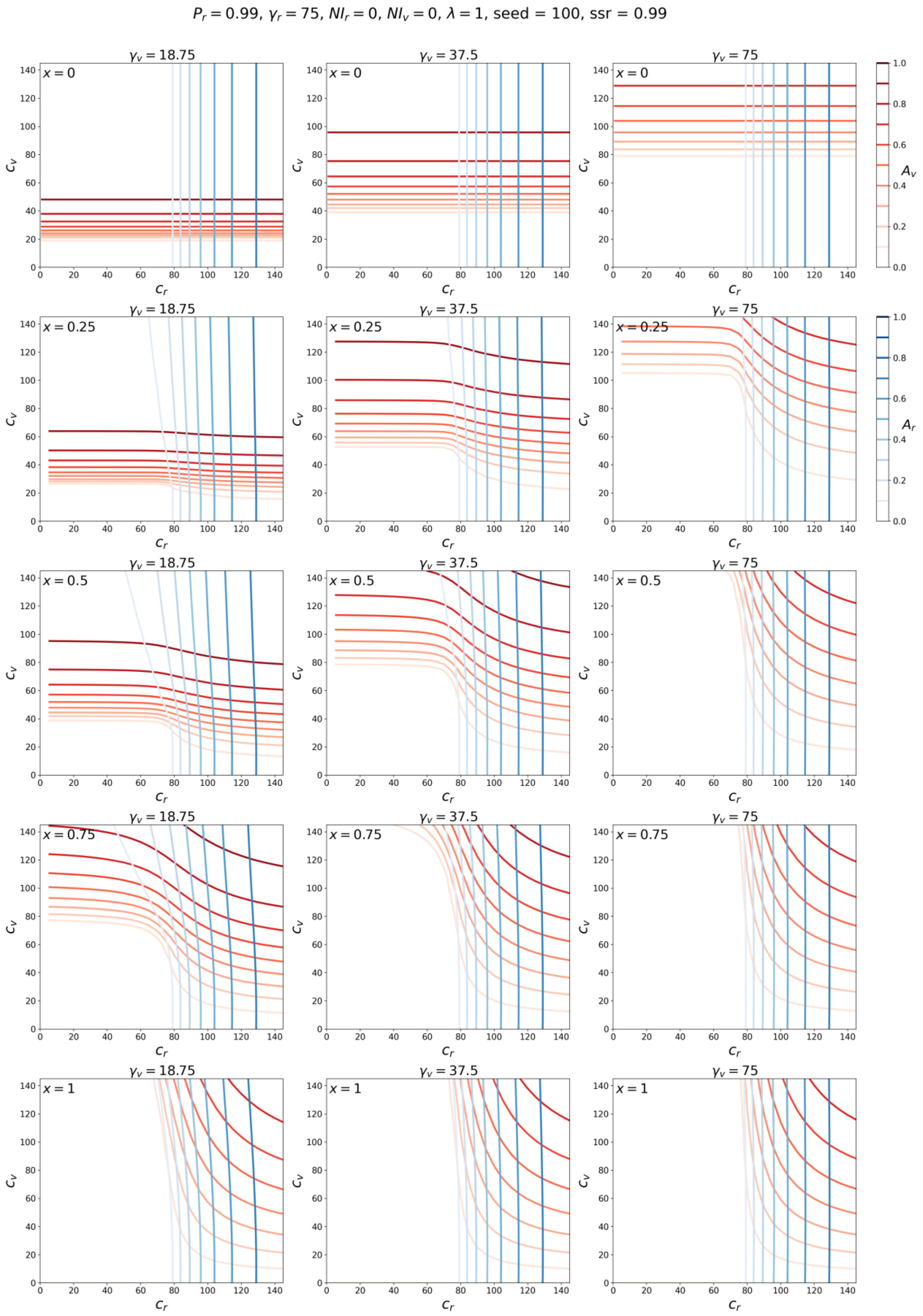
$P_r = 0.99$, $\gamma_r = 75$, $NI_r = 0$, $NI_v = 0$, $\lambda = 1$, seed = 100, ssr = 0.99
$\gamma_v = 18.75$
$\gamma_v = 37.5$
$\gamma_v = 75$
$x = 0$
$x = 0.25$
$x = 0.5$
$x = 0.75$
$x = 1$
c_v
c_r
A_v
A_r

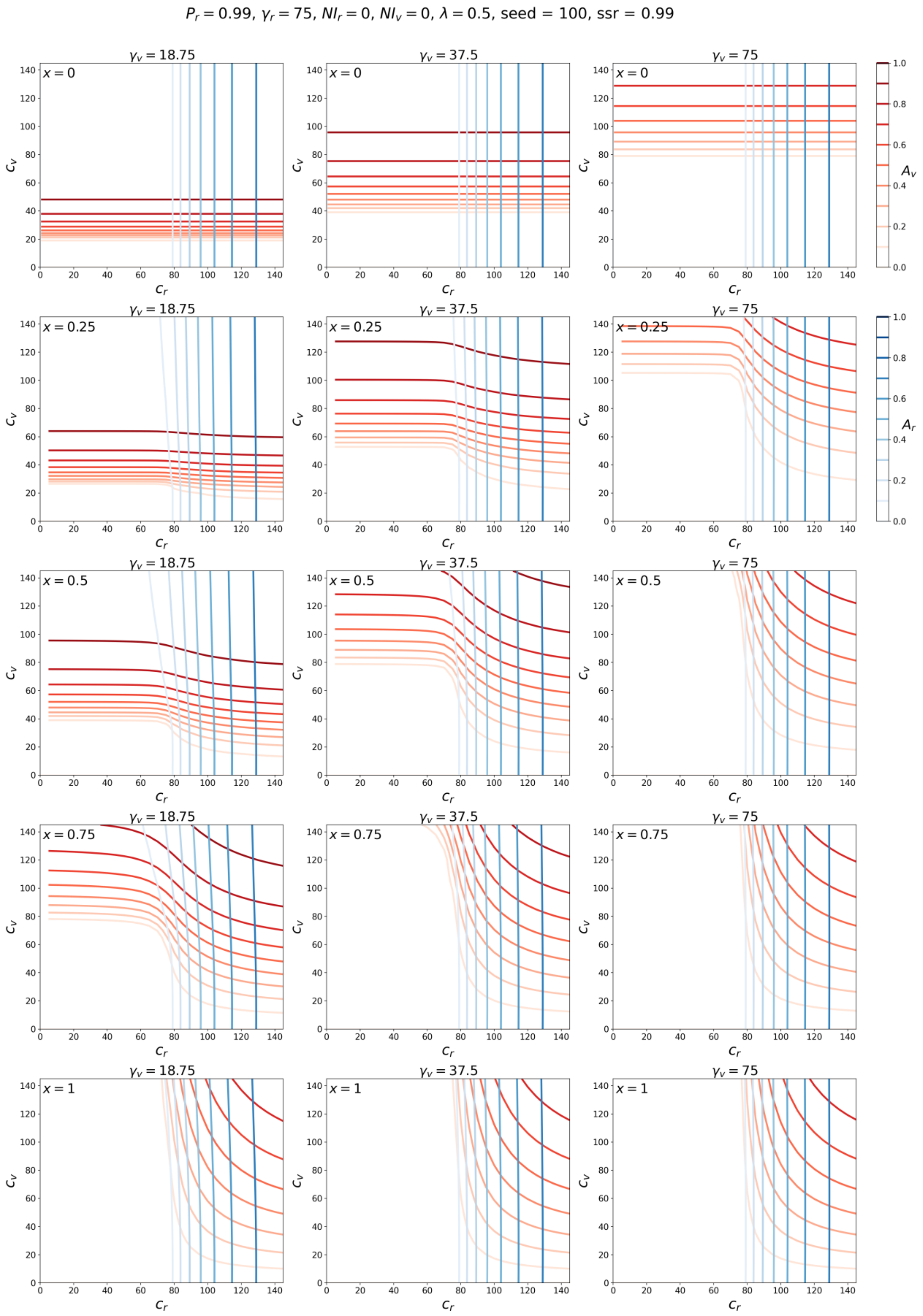
$P_r = 0.99$, $\gamma_r = 75$, $NI_r = 0$, $NI_v = 0$, $\lambda = 0.5$, seed = 100, ssr = 0.99
$\gamma_v = 18.75$
$\gamma_v = 37.5$
$\gamma_v = 75$
$x = 0$
$x = 0.25$
$x = 0.5$
$x = 0.75$
$x = 1$
c_v
c_r
A_v
A_r

$P_r = 0.99$, $\gamma_r = 75$, $NI_r = 0$, $NI_v = 0$, $\lambda = 0$, seed = 100, ssr = 0.99

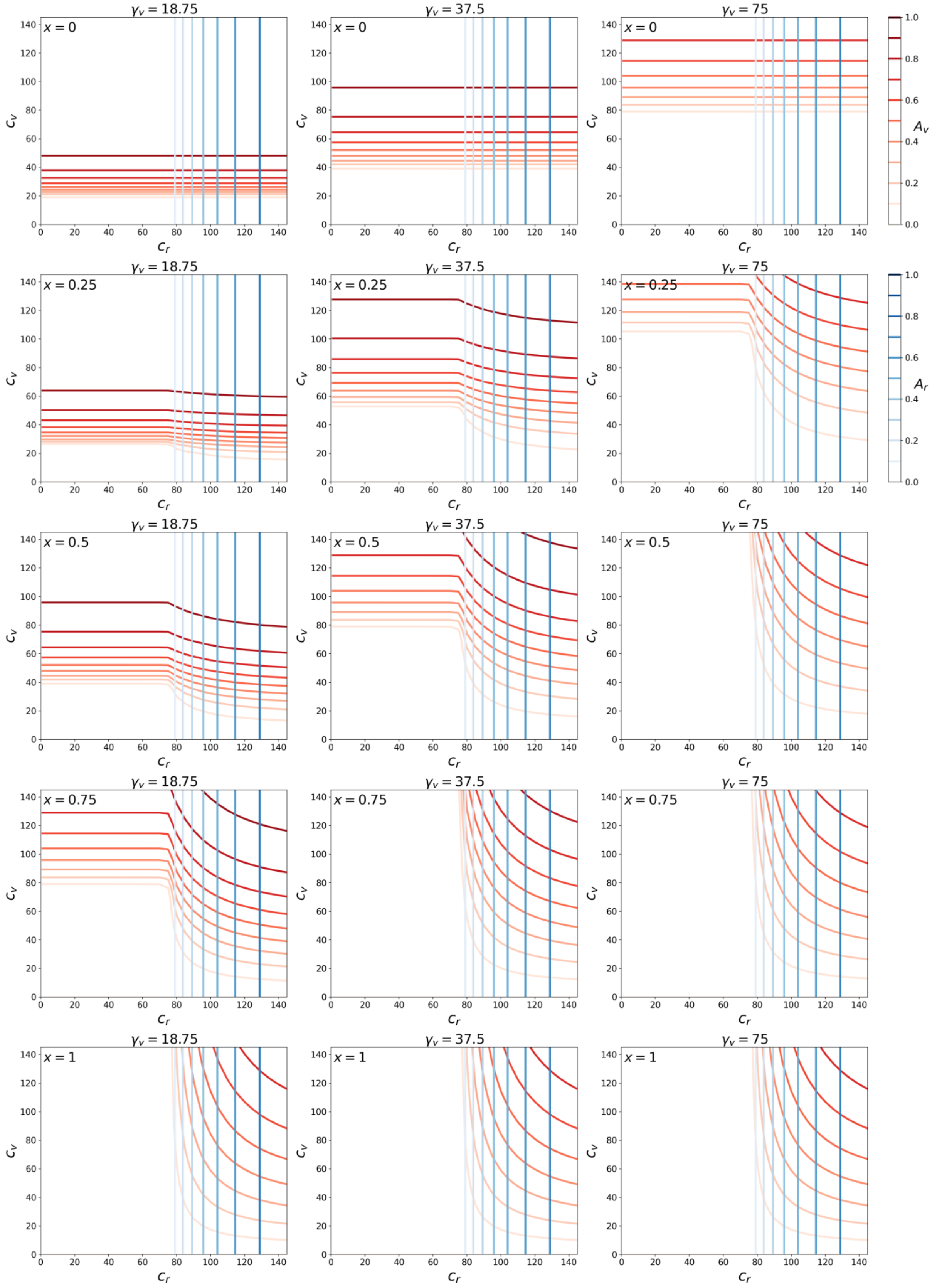

B.2: Attack-rate contour maps for different values of λ and for P_r = 0.8

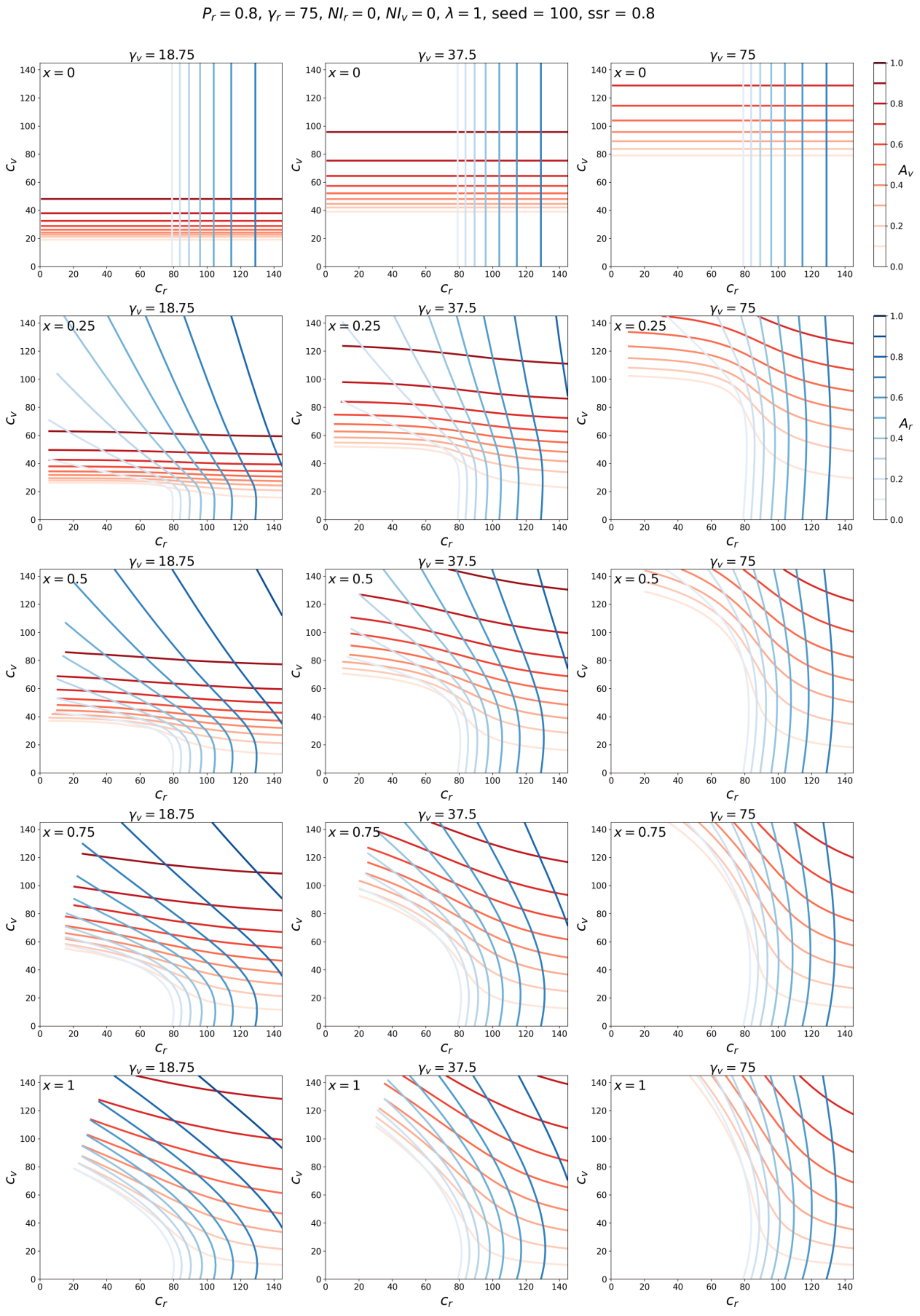
$P_r = 0.8$, $\gamma_r = 75$, $NI_r = 0$, $NI_v = 0$, $\lambda = 1$, seed = 100, ssr = 0.8
$\gamma_v = 18.75$
$\gamma_v = 37.5$
$\gamma_v = 75$
$x = 0$
$x = 0.25$
$x = 0.5$
$x = 0.75$
$x = 1$
c_v
c_r
A_v
A_r

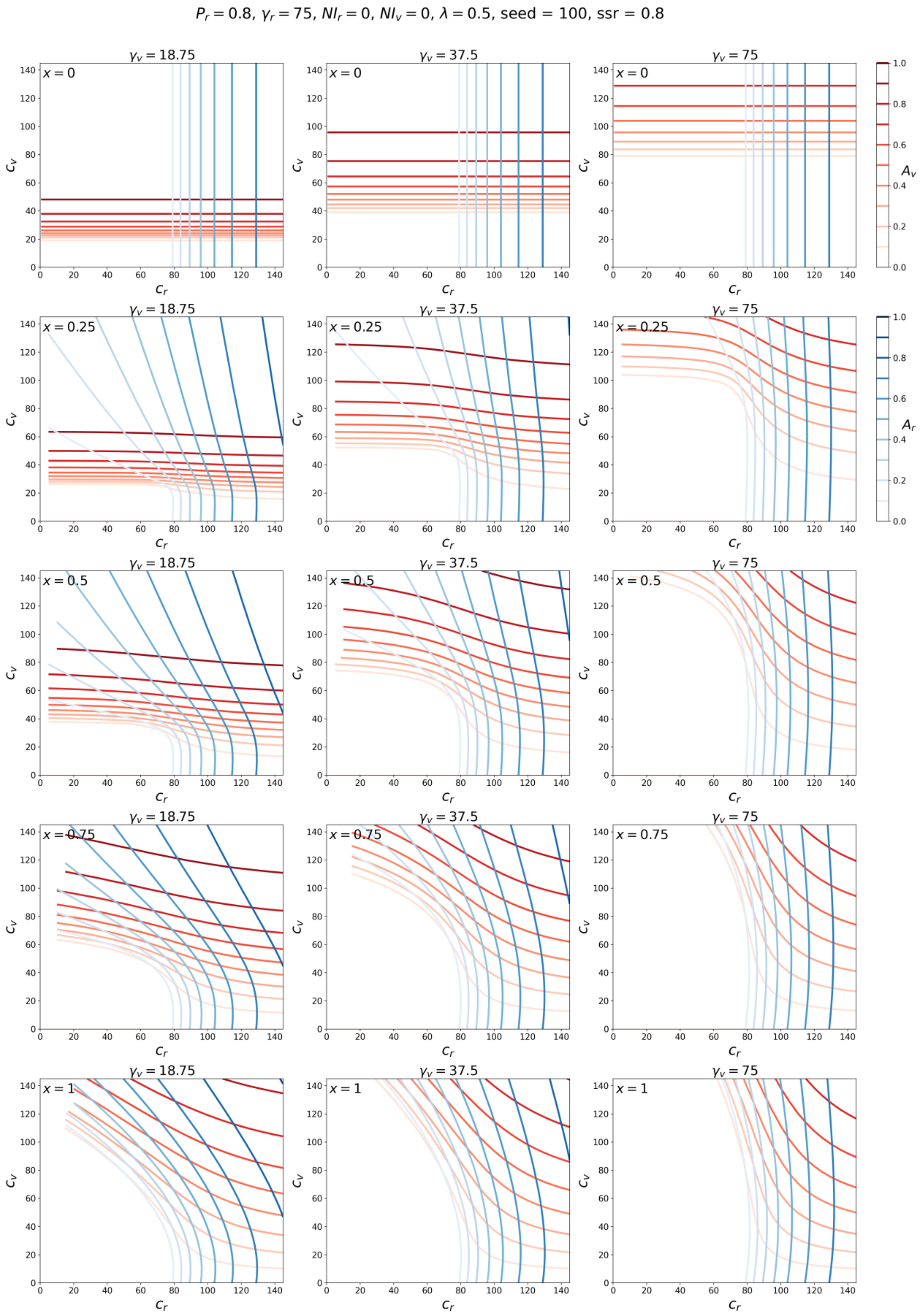
$P_r = 0.8$, $\gamma_r = 75$, $NI_r = 0$, $NI_v = 0$, $\lambda = 0.5$, seed = 100, ssr = 0.8
$\gamma_v = 18.75$
$\gamma_v = 37.5$
$\gamma_v = 75$
$x = 0$
$x = 0.25$
$x = 0.5$
$x = 0.75$
$x = 1$
c_v
c_r
A_v
A_r

$P_r = 0.8$, $\gamma_r = 75$, $NI_r = 0$, $NI_v = 0$, $\lambda = 0$, seed = 100, ssr = 0.8

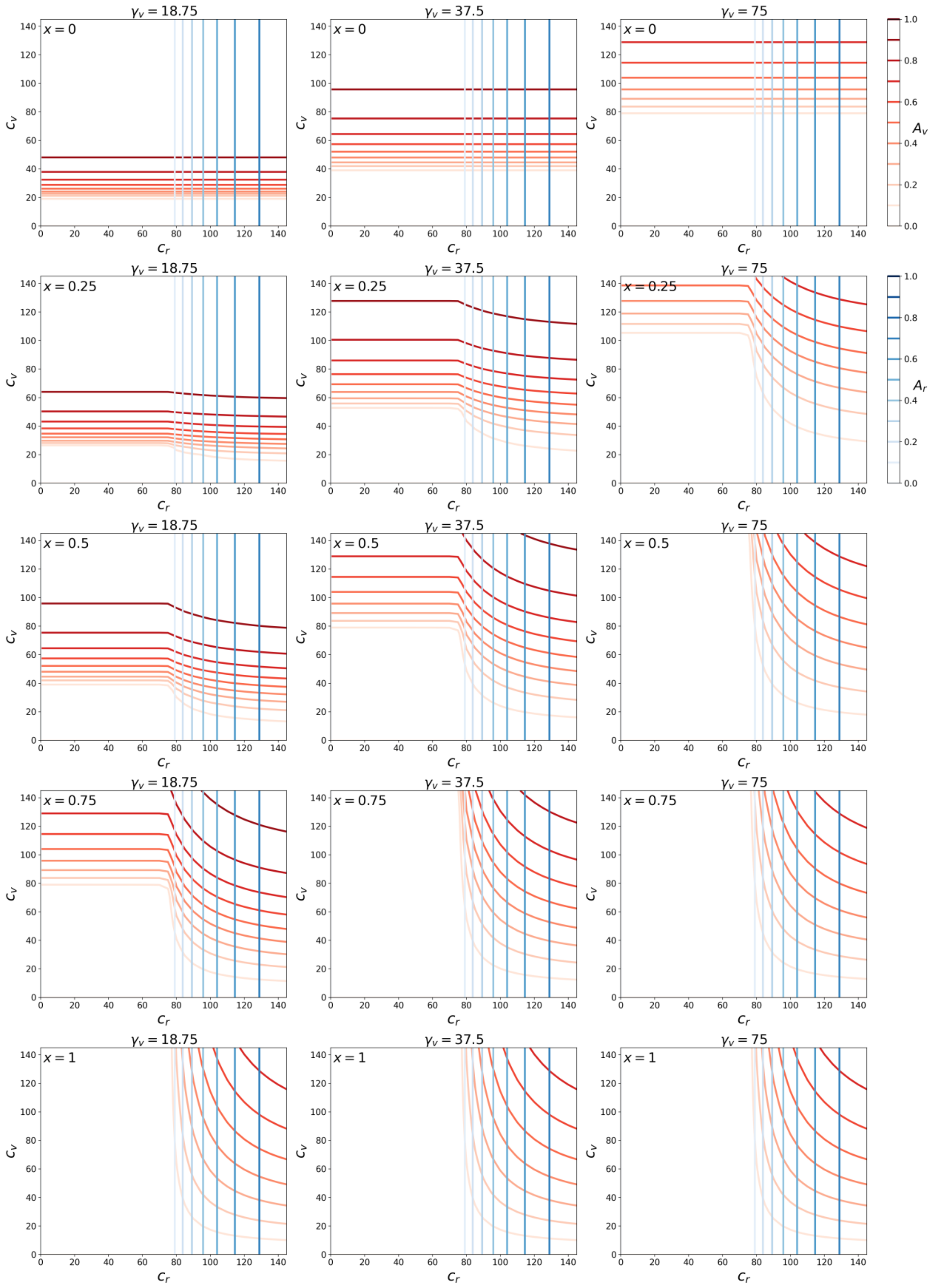

B.3: Attack-rate contour maps for different values of λ and for P_r = 0.6

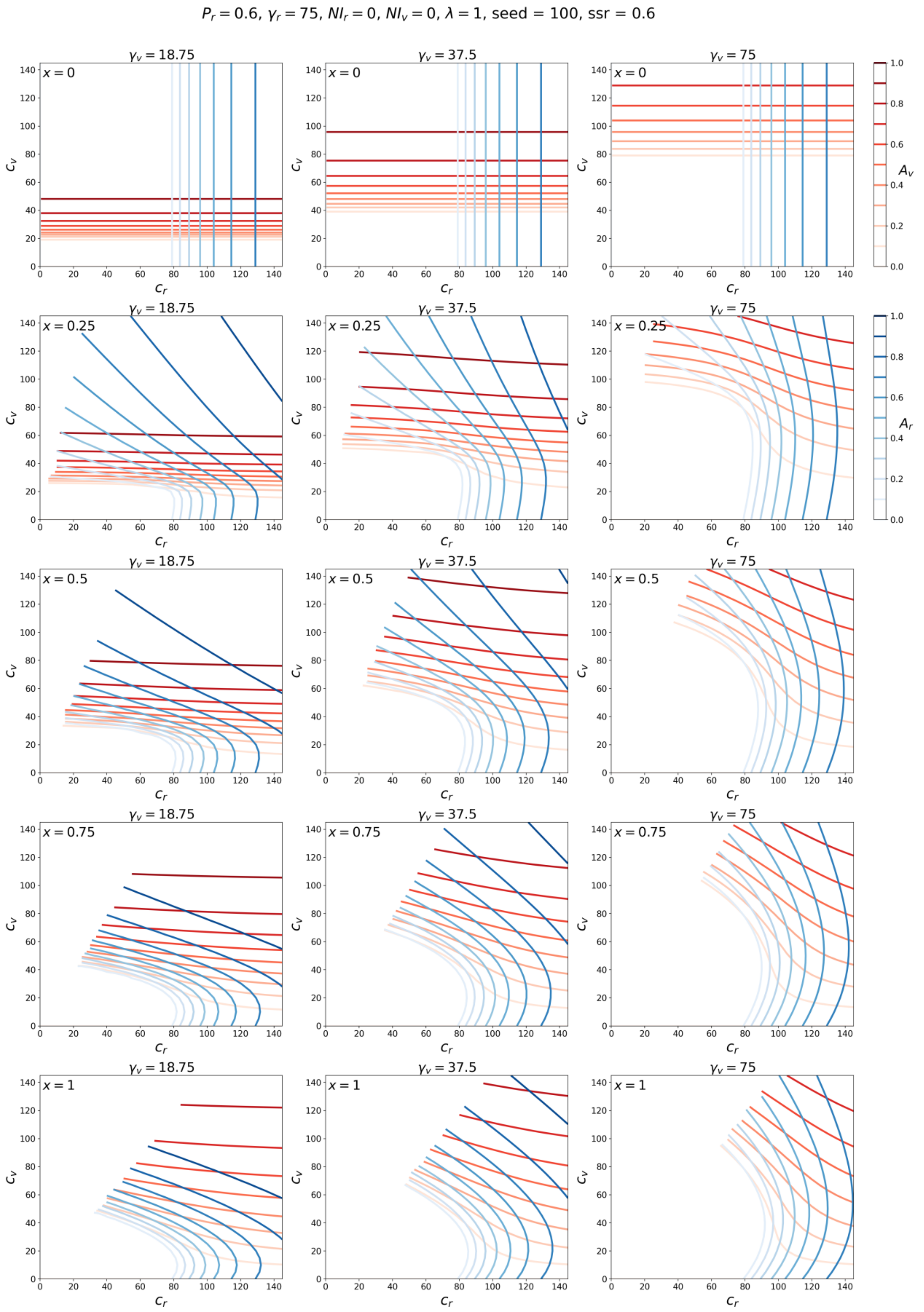

$P_r = 0.6$, $\gamma_r = 75$, $NI_r = 0$, $NI_v = 0$, $\lambda = 1$, seed = 100, ssr = 0.6
$\gamma_v = 18.75$
$\gamma_v = 37.5$
$\gamma_v = 75$
$x = 0$
$x = 0.25$
$x = 0.5$
$x = 0.75$
$x = 1$
c_v
c_r
A_v
A_r

$P_r = 0.6$, $\gamma_r = 75$, $NI_r = 0$, $NI_v = 0$, $\lambda = 0.5$, seed = 100, ssr = 0.6

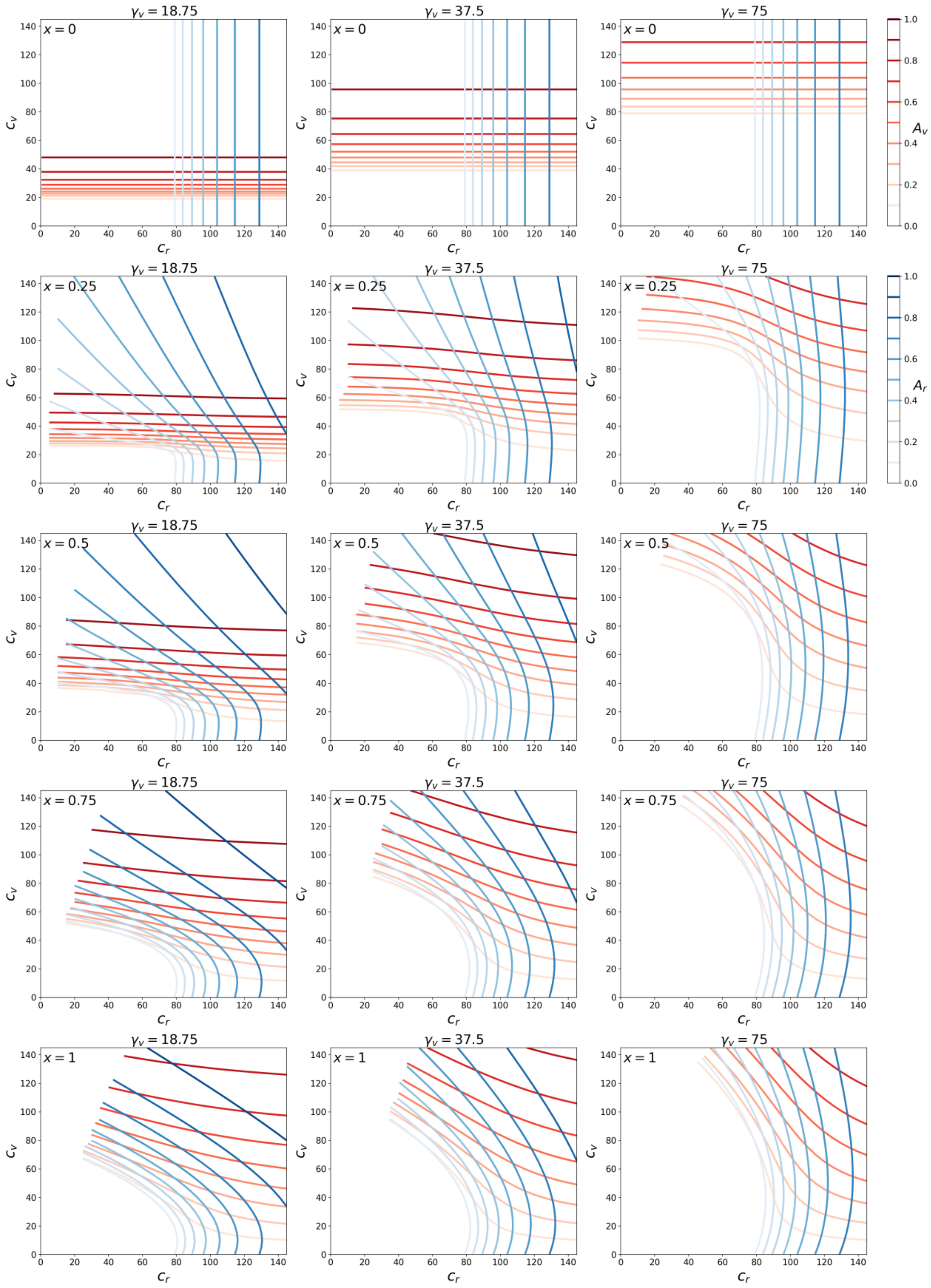

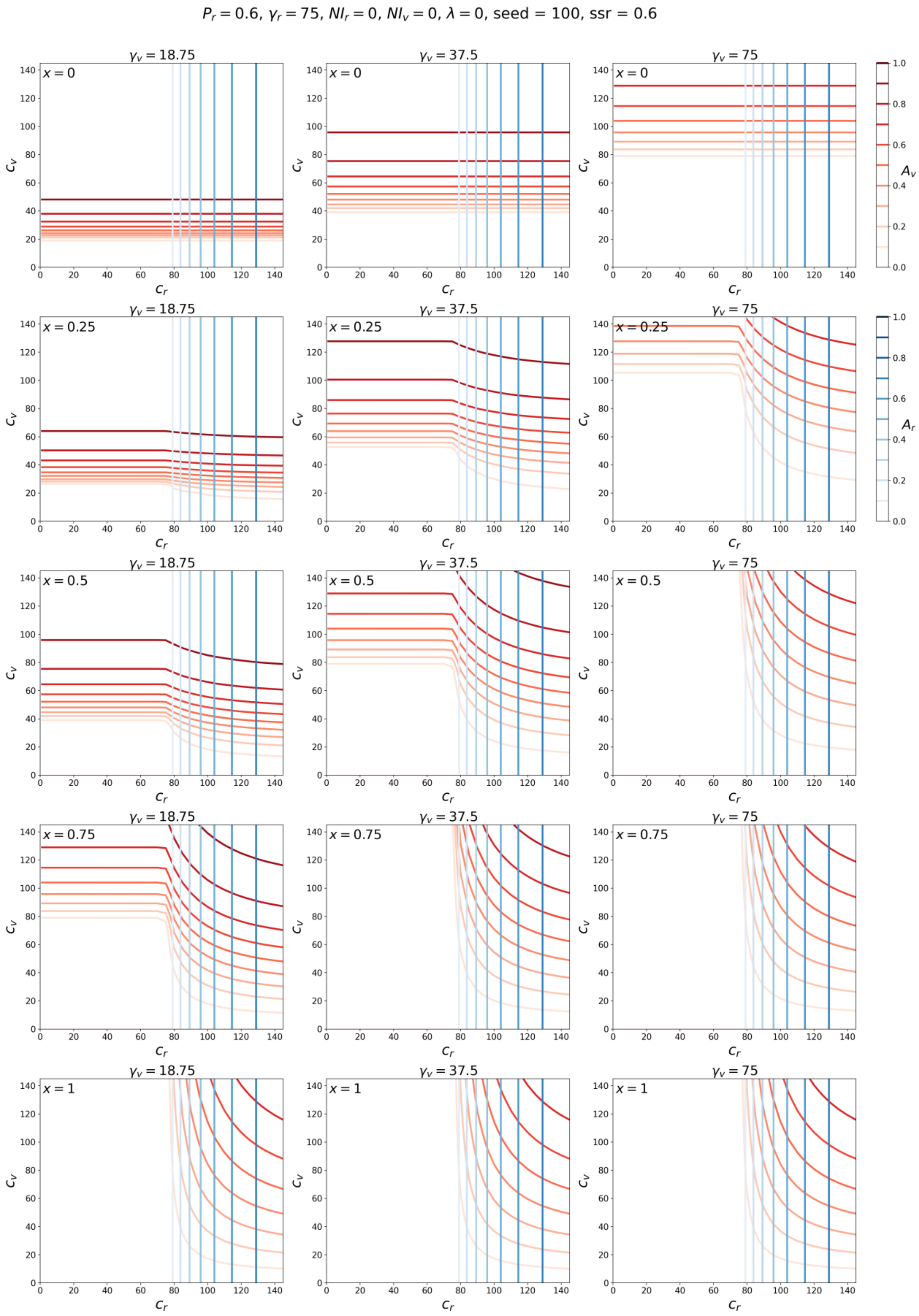
$P_r = 0.6$, $\gamma_r = 75$, $NI_r = 0$, $NI_v = 0$, $\lambda = 0$, seed = 100, ssr = 0.6
$\gamma_v = 18.75$
$\gamma_v = 37.5$
$\gamma_v = 75$
$x = 0$
$x = 0.25$
$x = 0.5$
$x = 0.75$
$x = 1$
c_v
c_r
A_v
A_r

Appendix C: Varying seed distribution

The figures in Appendix C show contour maps for P_r = 0.99, P_r = 0.95, P_r = 0.8, and P_r = 0.6, with the initial 100 infected "seed" individuals placed entirely in the *r* population (ssr = 1). That is, none of the *v* individuals are initially infected, in the simulations shown in Appendix C.

As can be seen from the figures below, placing all "seed" individuals in the *r* group has no effect on the resulting attack rates as functions of c_r, c_v, and *x*, except for the trivial case of $x = 0$, in which it is impossible for any *v* person to become infected, since $x = 0$ means that $c_{vr} = 0$.

C.1: Attack-rate contour maps for different values of λ, for P_r = 0.99, seed = 100, and ssr = 1

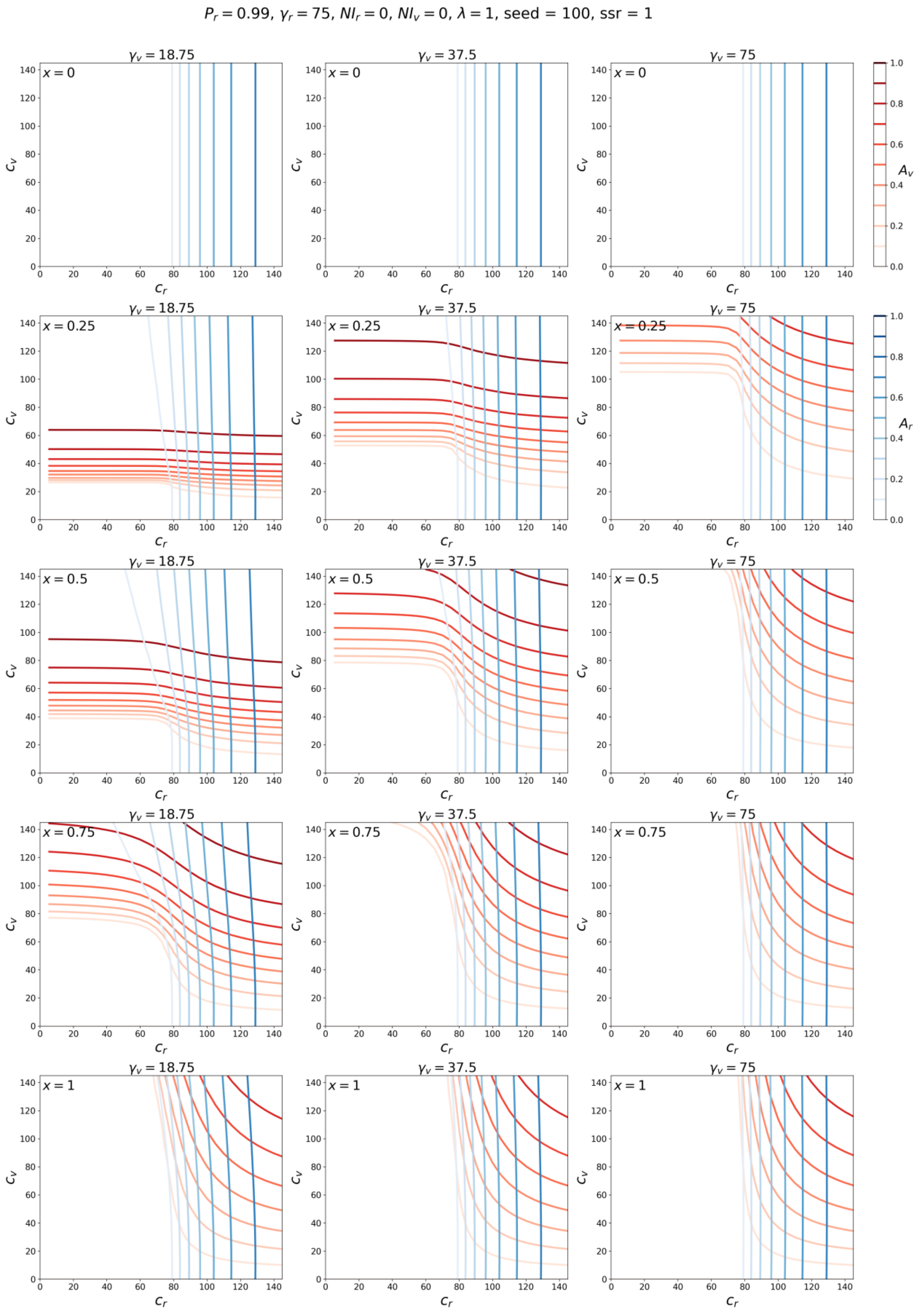
$P_r = 0.99$, $\gamma_r = 75$, $NI_r = 0$, $NI_v = 0$, $\lambda = 1$, seed = 100, ssr = 1
$\gamma_v = 18.75$
$\gamma_v = 37.5$
$\gamma_v = 75$
$x = 0$
$x = 0.25$
$x = 0.5$
$x = 0.75$
$x = 1$
c_v
c_r
A_v
A_r

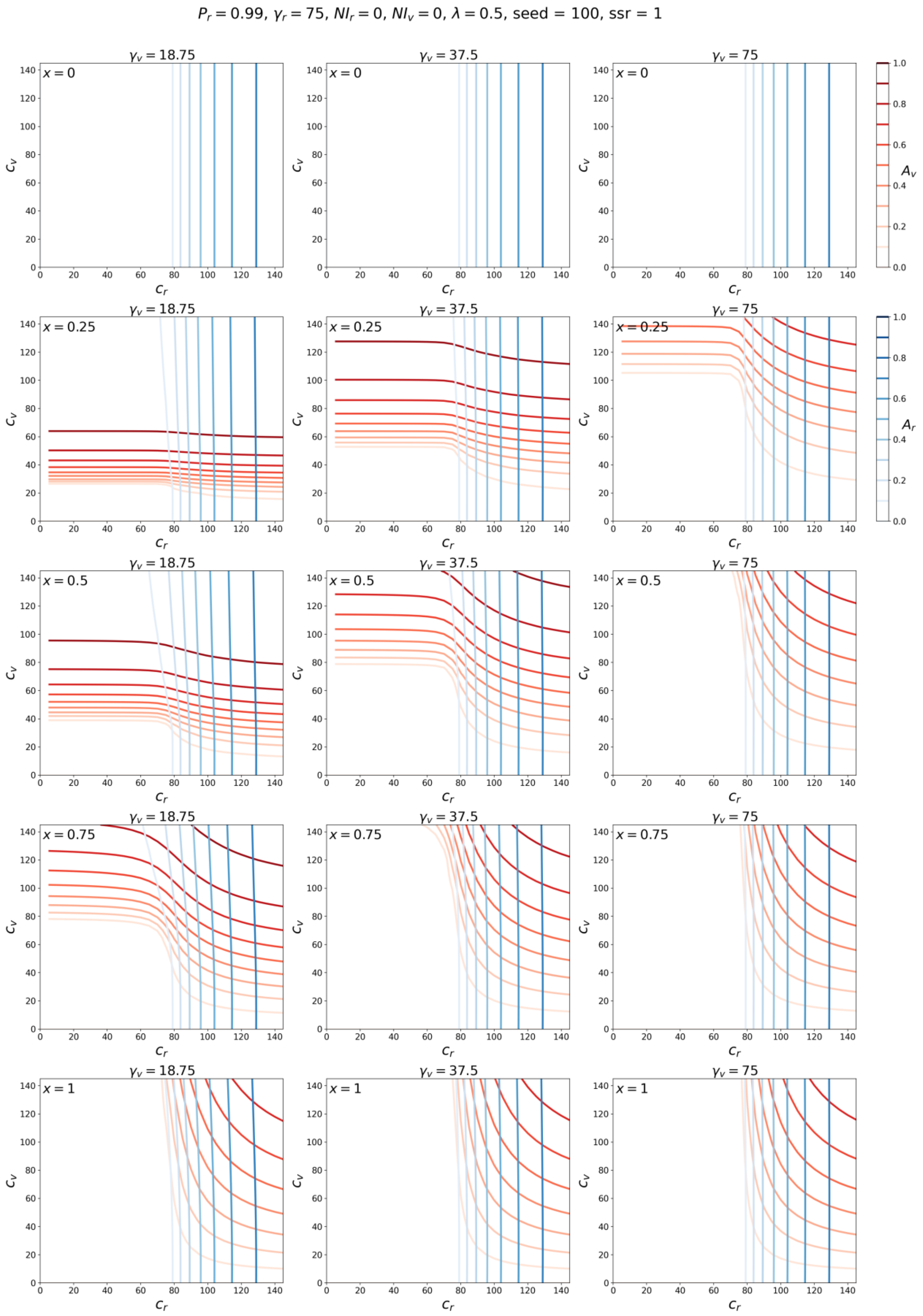
$P_r = 0.99$, $\gamma_r = 75$, $NI_r = 0$, $NI_v = 0$, $\lambda = 0.5$, seed = 100, ssr = 1
$\gamma_v = 18.75$
$\gamma_v = 37.5$
$\gamma_v = 75$
$x = 0$
$x = 0.25$
$x = 0.5$
$x = 0.75$
$x = 1$
c_v
c_r
A_v
A_r

$P_r = 0.99$, $\gamma_r = 75$, $NI_r = 0$, $NI_v = 0$, $\lambda = 0$, seed = 100, ssr = 1

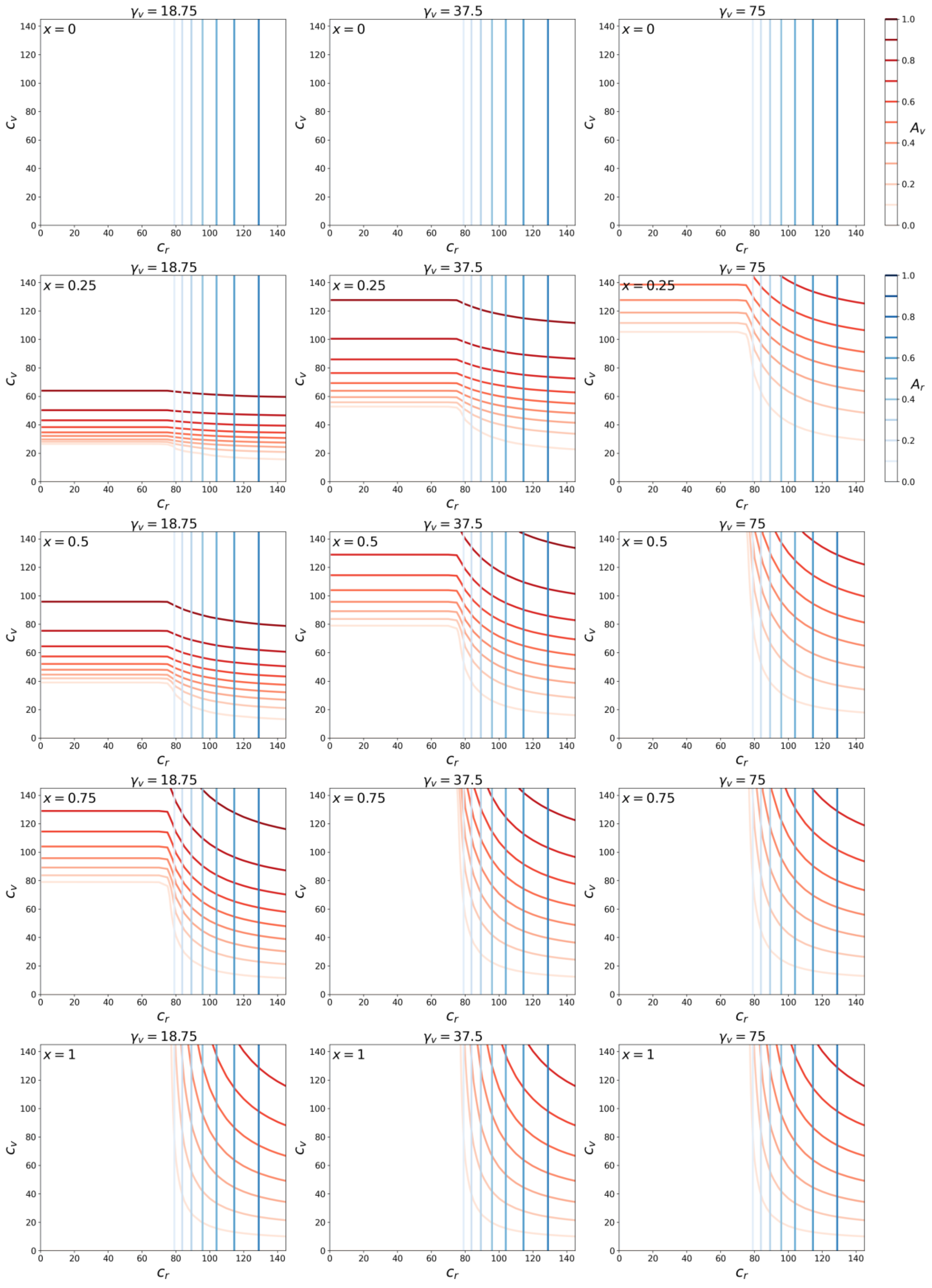

C.2: Attack-rate contour maps for different values of λ, for P_r = 0.95, seed = 100, and ssr = 1

$P_r = 0.95$, $\gamma_r = 75$, $NI_r = 0$, $NI_v = 0$, $\lambda = 1$, seed = 100, ssr = 1

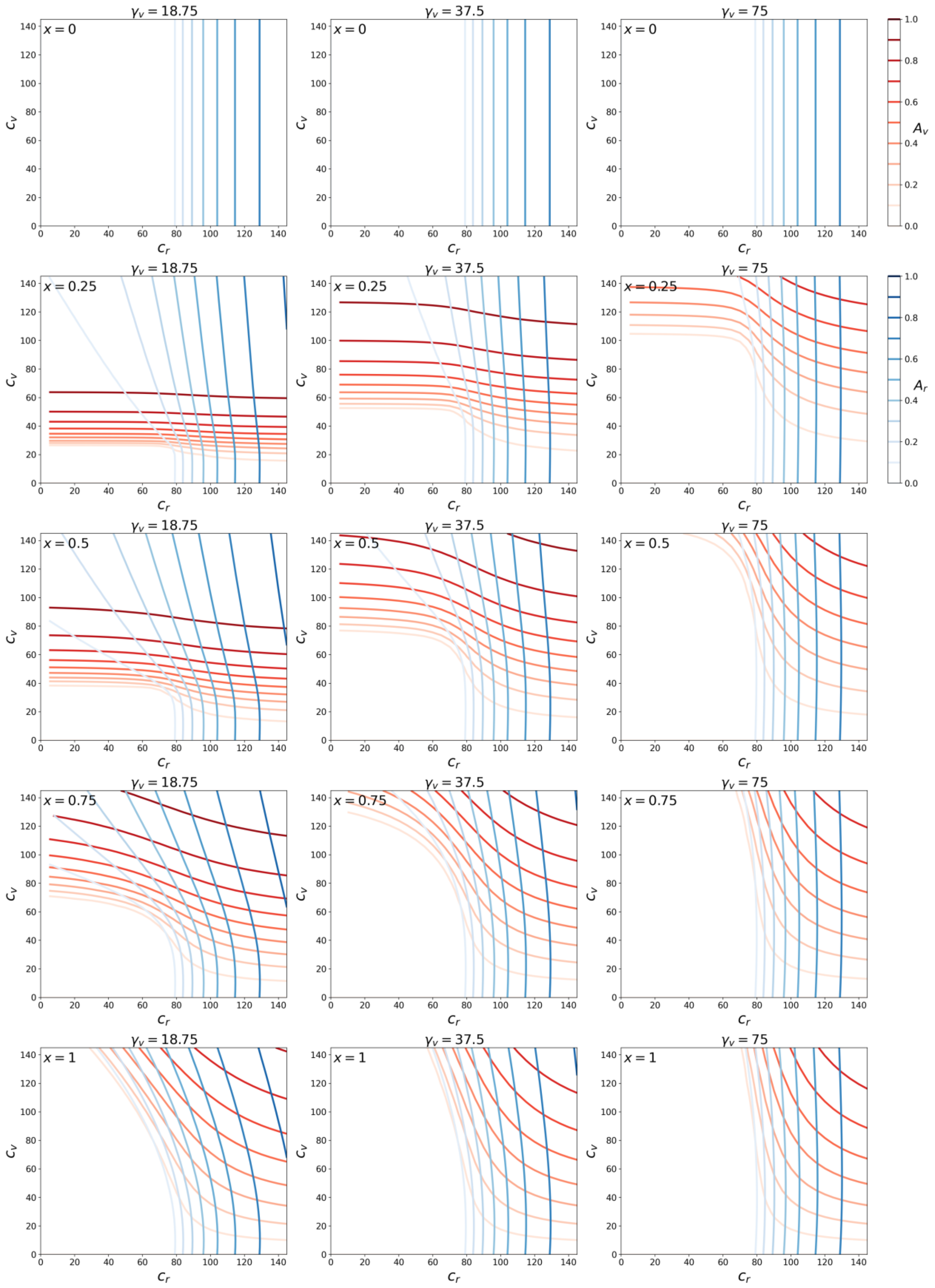

$P_r = 0.95$, $\gamma_r = 75$, $NI_r = 0$, $NI_v = 0$, $\lambda = 0.5$, seed = 100, ssr = 1

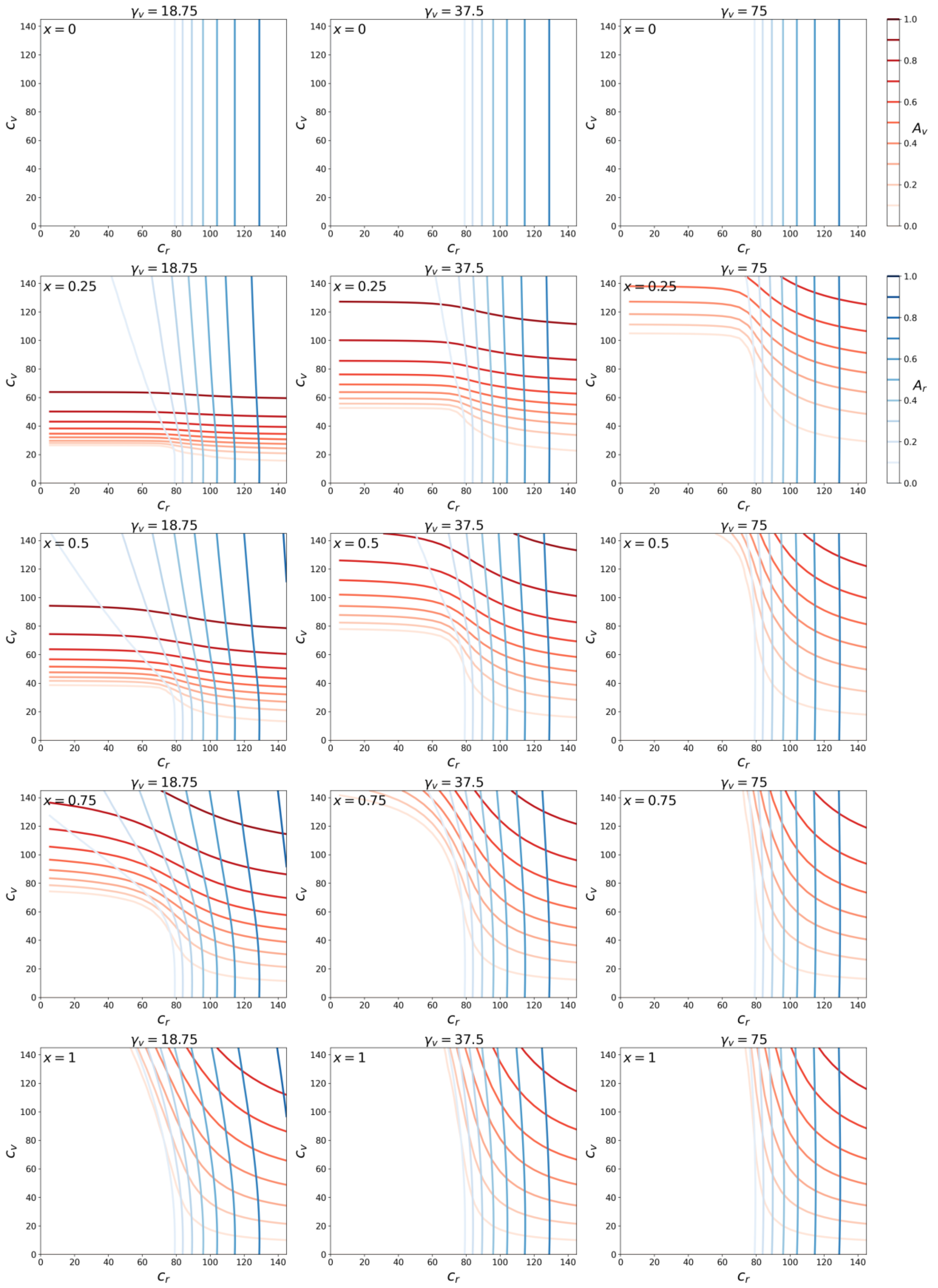

$P_r = 0.95$, $\gamma_r = 75$, $NI_r = 0$, $NI_v = 0$, $\lambda = 0$, seed = 100, ssr = 1

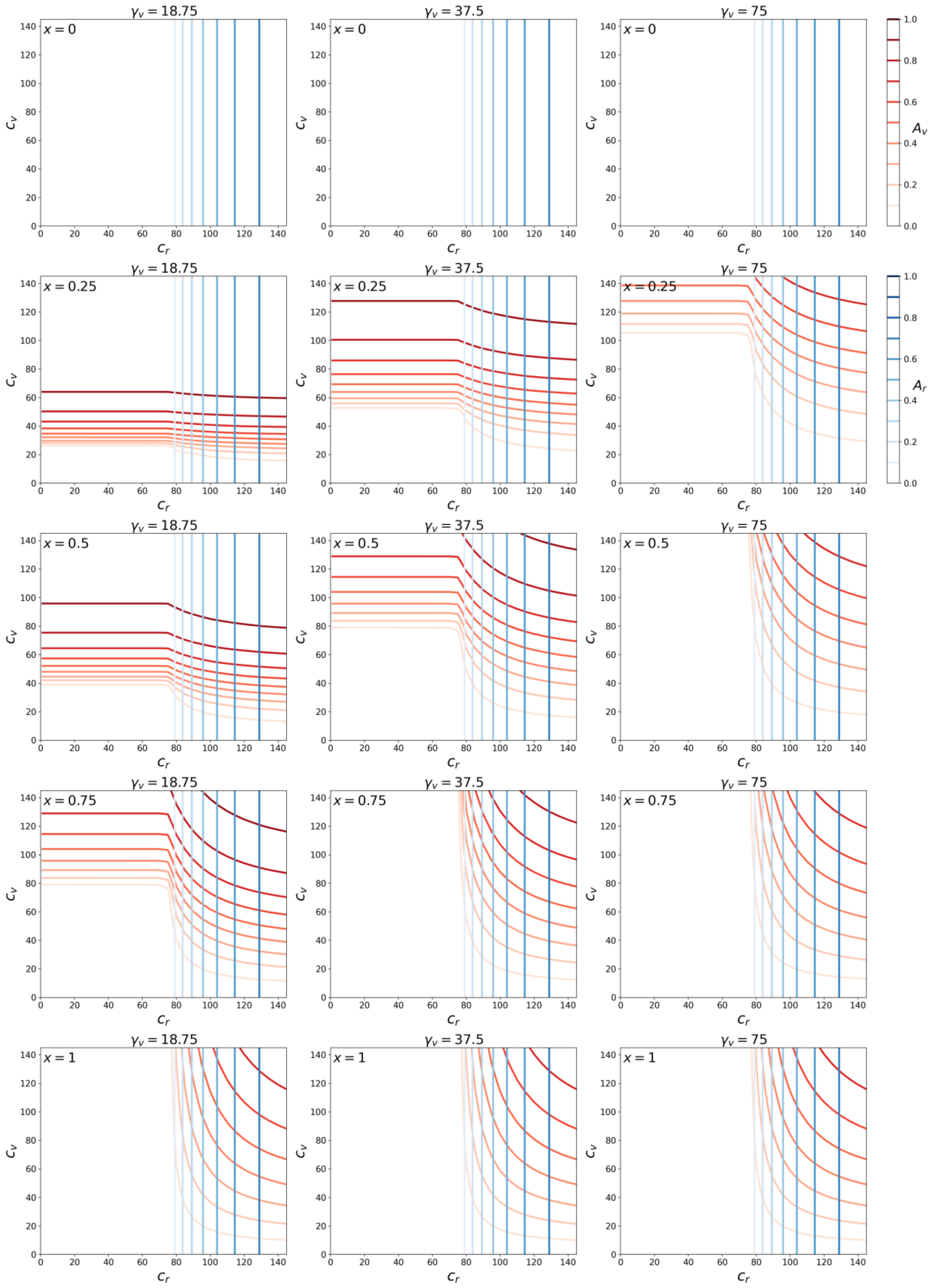

C.3: Attack-rate contour maps for different values of λ, for P_r = 0.8, seed = 100, and ssr = 1

$P_r = 0.8$, $\gamma_r = 75$, $NI_r = 0$, $NI_v = 0$, $\lambda = 1$, seed = 100, ssr = 1

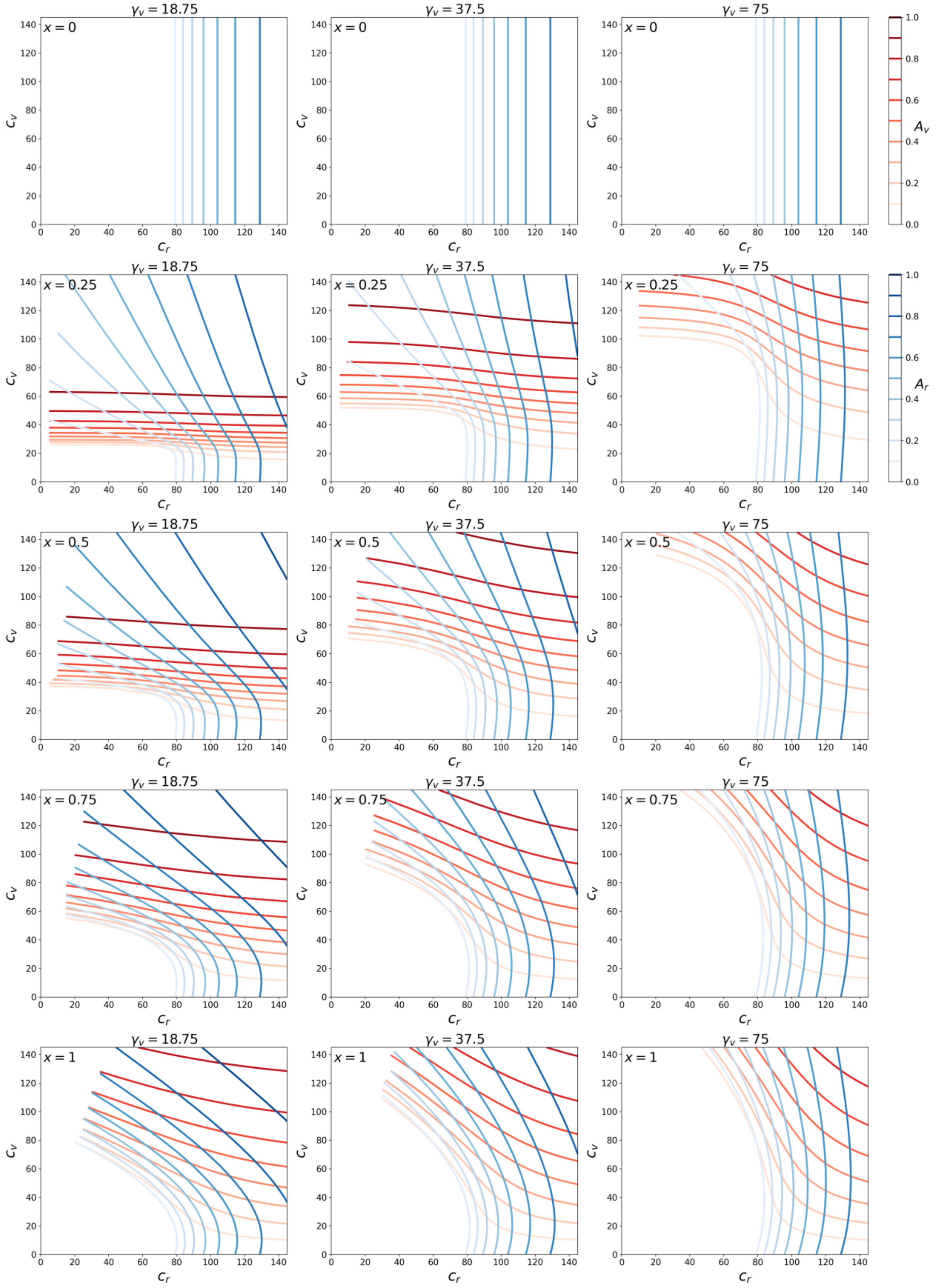

$P_r = 0.8$, $\gamma_r = 75$, $NI_r = 0$, $NI_v = 0$, $\lambda = 0.5$, seed = 100, ssr = 1

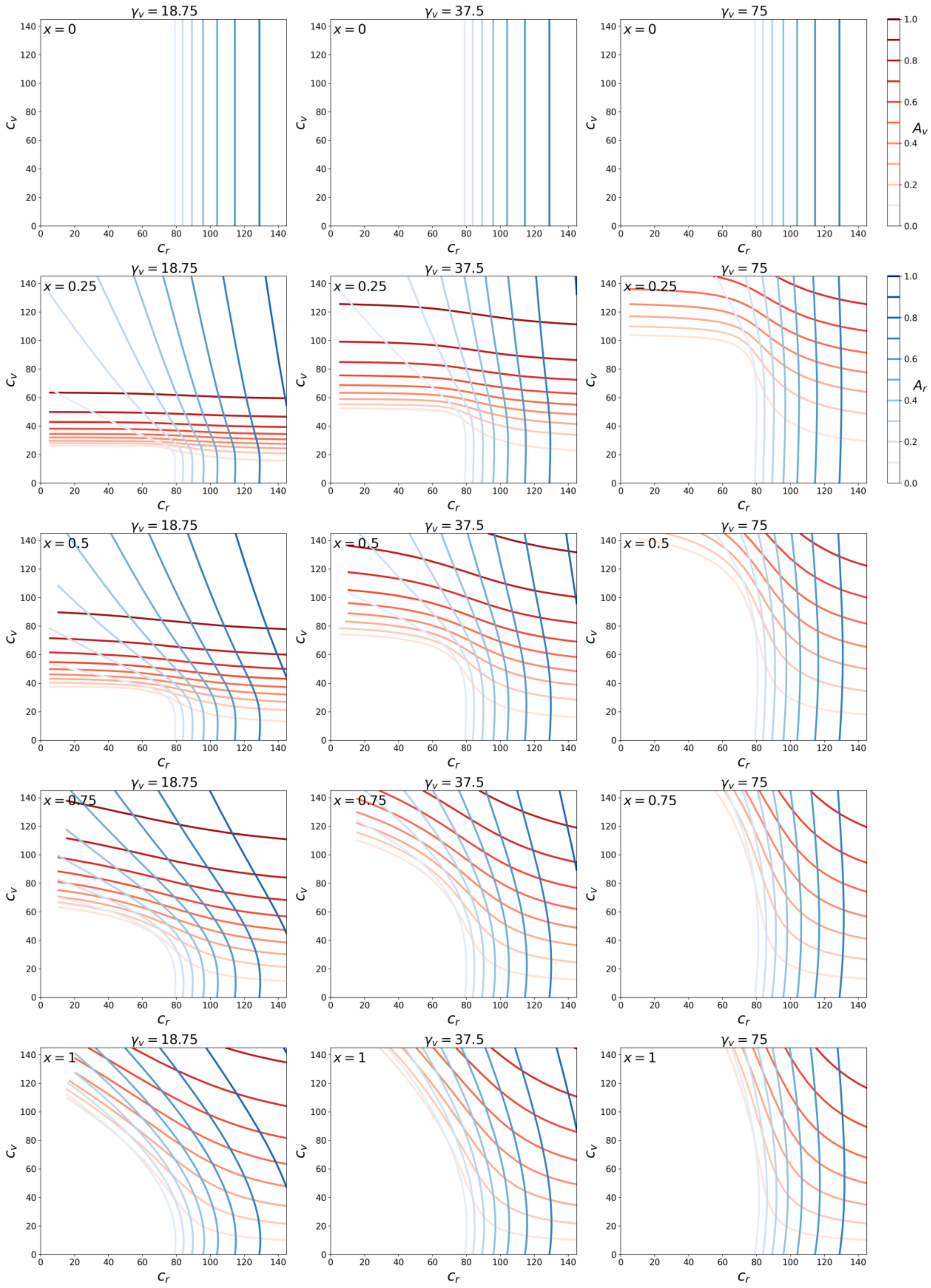

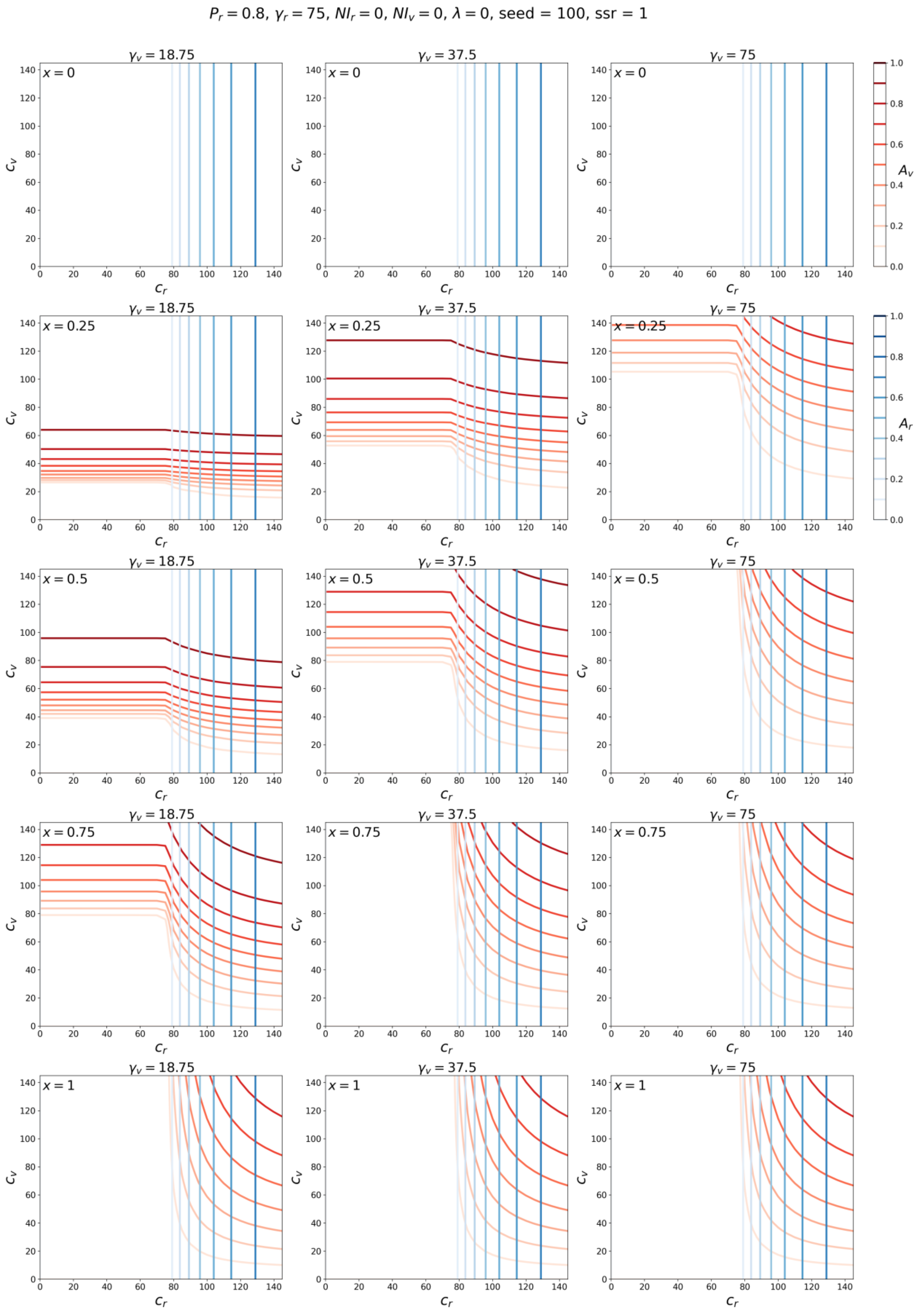
P_r = 0.8, γ_r = 75, NI_r = 0, NI_v = 0, λ = 0, seed = 100, ssr = 1
γ_v = 18.75
γ_v = 37.5
γ_v = 75
x = 0
x = 0.25
x = 0.5
x = 0.75
x = 1
c_v
c_r
A_v
A_r

C.4: Attack-rate contour maps for different values of λ, for P_r = 0.6, seed = 100, and ssr = 1

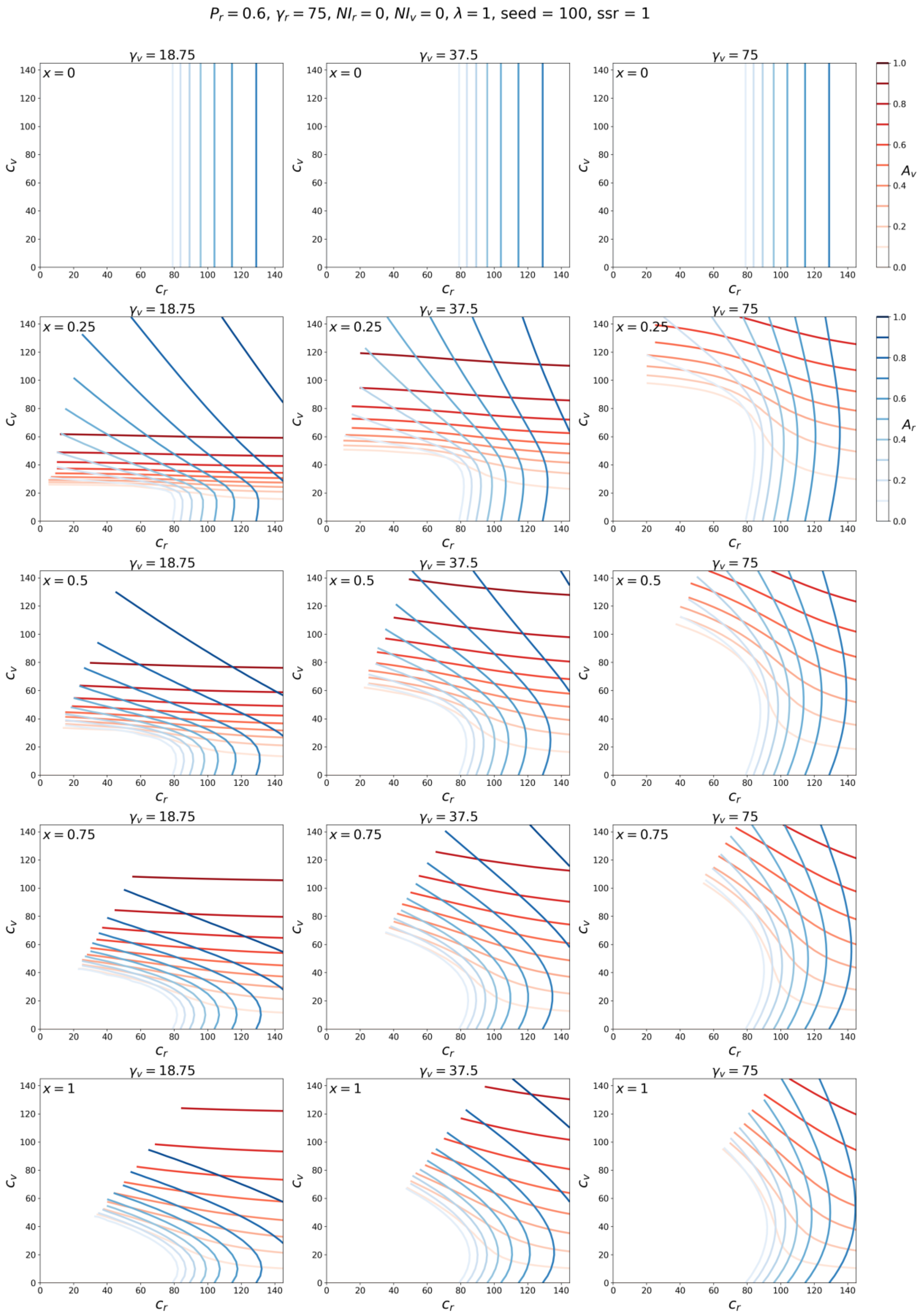
$P_r = 0.6$, $\gamma_r = 75$, $NI_r = 0$, $NI_v = 0$, $\lambda = 1$, seed = 100, ssr = 1
$\gamma_v = 18.75$
$\gamma_v = 37.5$
$\gamma_v = 75$
$x = 0$
$x = 0.25$
$x = 0.5$
$x = 0.75$
$x = 1$
c_v
c_r
A_v
A_r

$P_r = 0.6$, $\gamma_r = 75$, $NI_r = 0$, $NI_v = 0$, $\lambda = 0.5$, seed = 100, ssr = 1

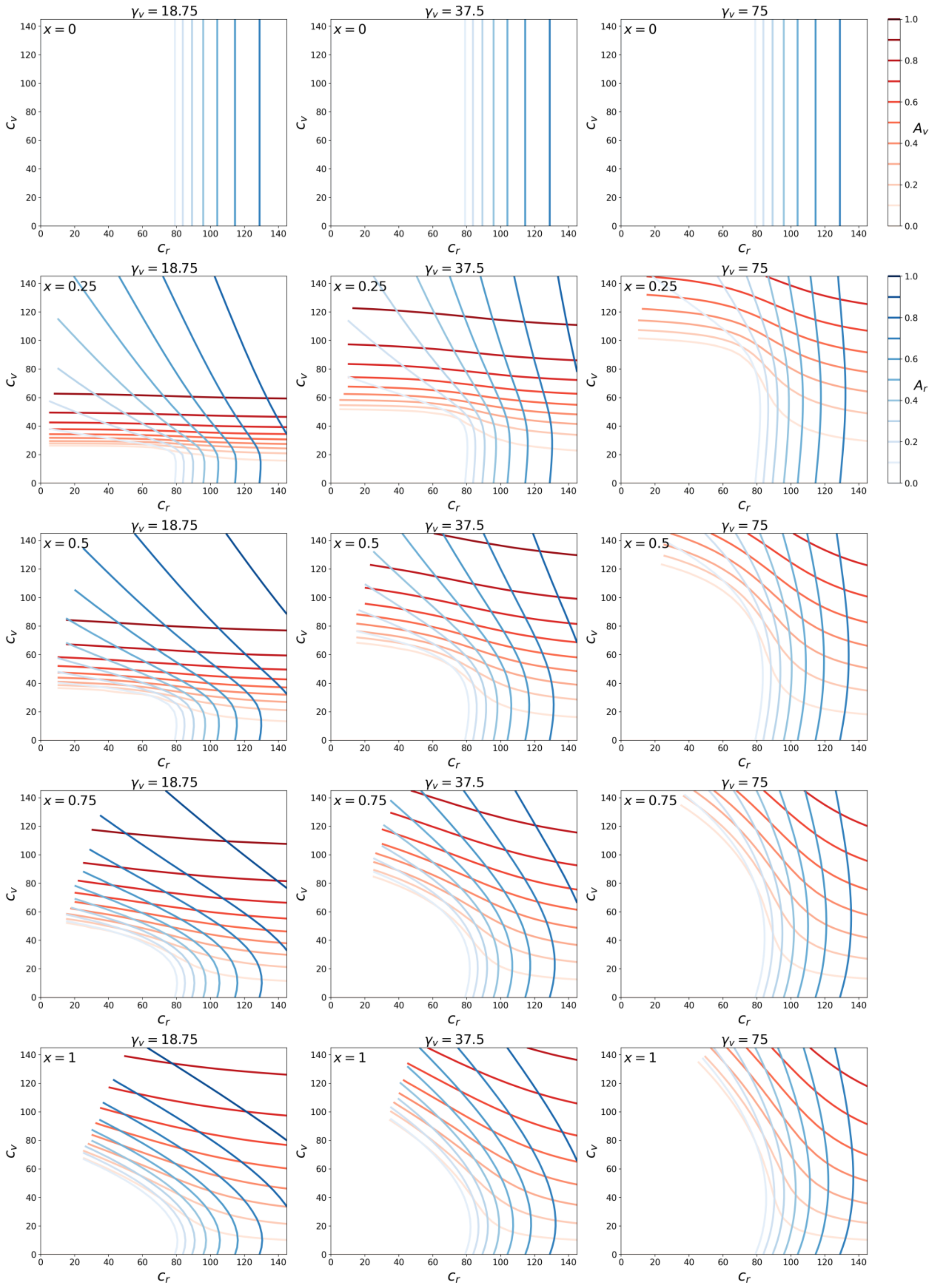

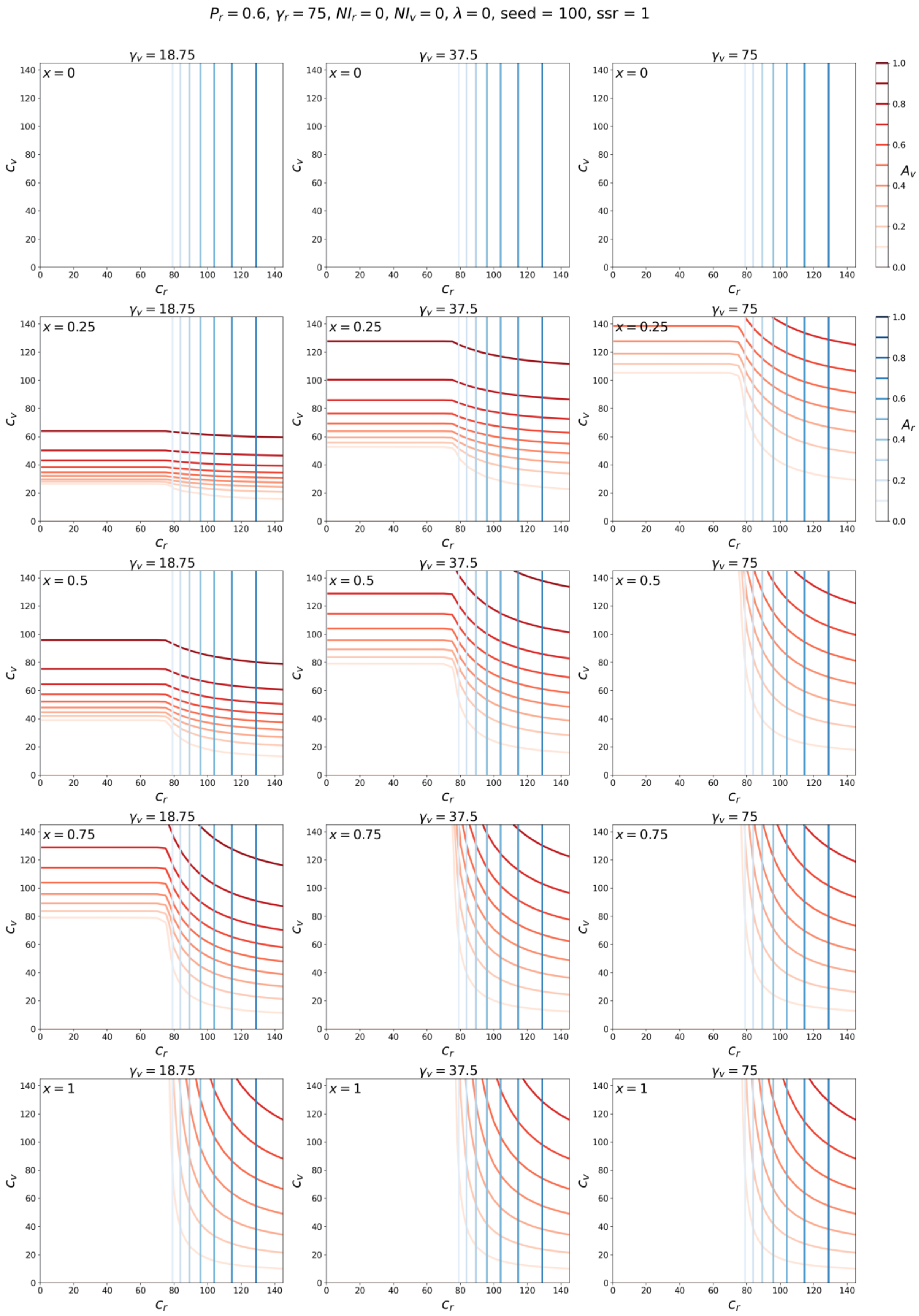
$P_r = 0.6$, $\gamma_r = 75$, $NI_r = 0$, $NI_v = 0$, $\lambda = 0$, seed = 100, ssr = 1
$\gamma_v = 18.75$
$\gamma_v = 37.5$
$\gamma_v = 75$
$x = 0$
$x = 0.25$
$x = 0.5$
$x = 0.75$
$x = 1$
c_v
c_r
A_v
A_r

Appendix D: Varying seed magnitude

This Appendix contains attack-rate contour maps for the parameters used in the main text figures, but for different magnitudes of the initial seed number of infected individuals (parameter “seed”). As can be seen, changing the seed magnitude does not change the attack-rate results.

$P_r = 0.95$, $\gamma_r = 75$, $NI_r = 0$, $NI_v = 0$, $\lambda = 1$, seed = 10, ssr = 0.95

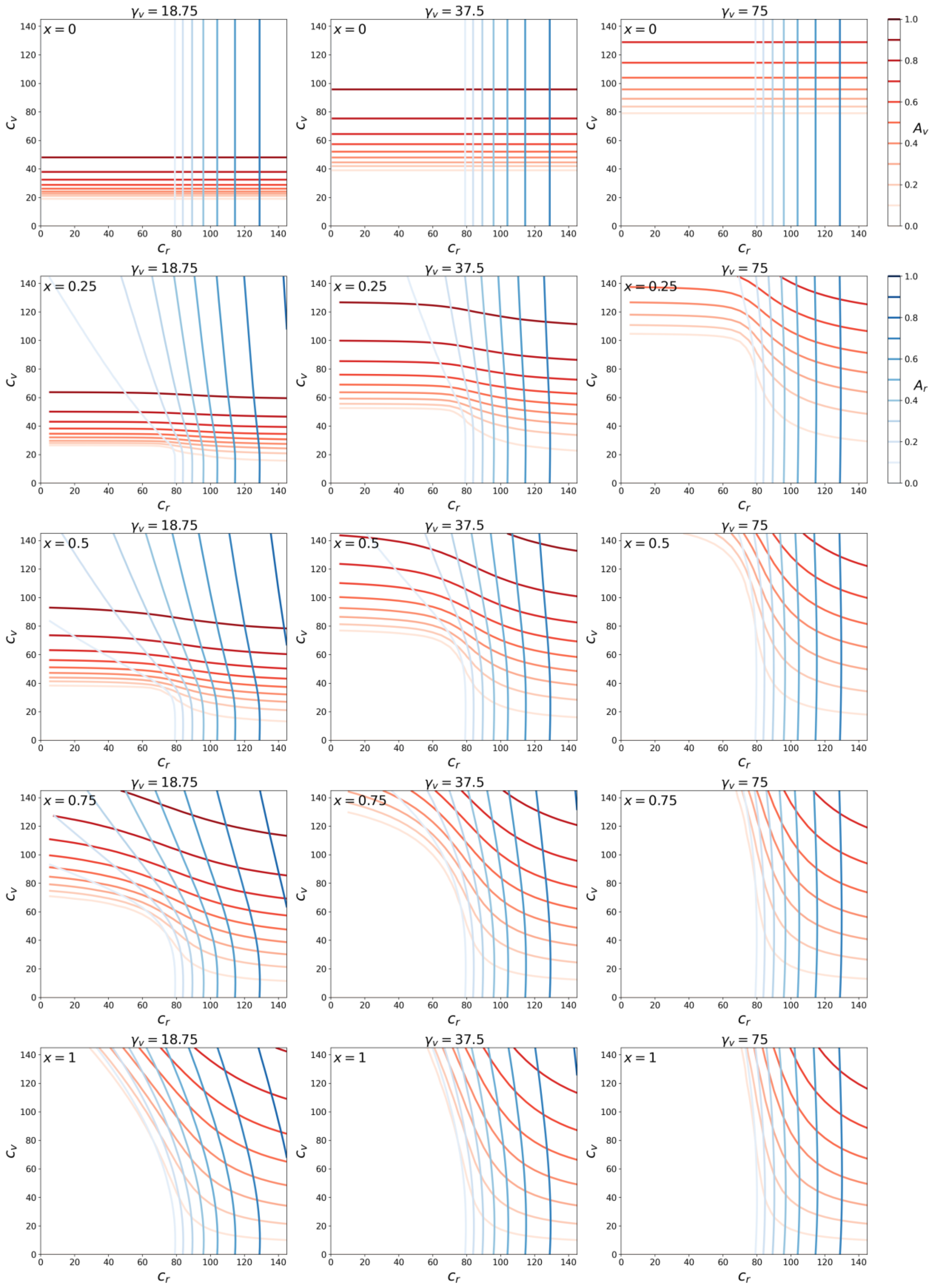

$P_r = 0.95$, $\gamma_r = 75$, $NI_r = 0$, $NI_v = 0$, $\lambda = 1$, seed = 100, ssr = 0.95

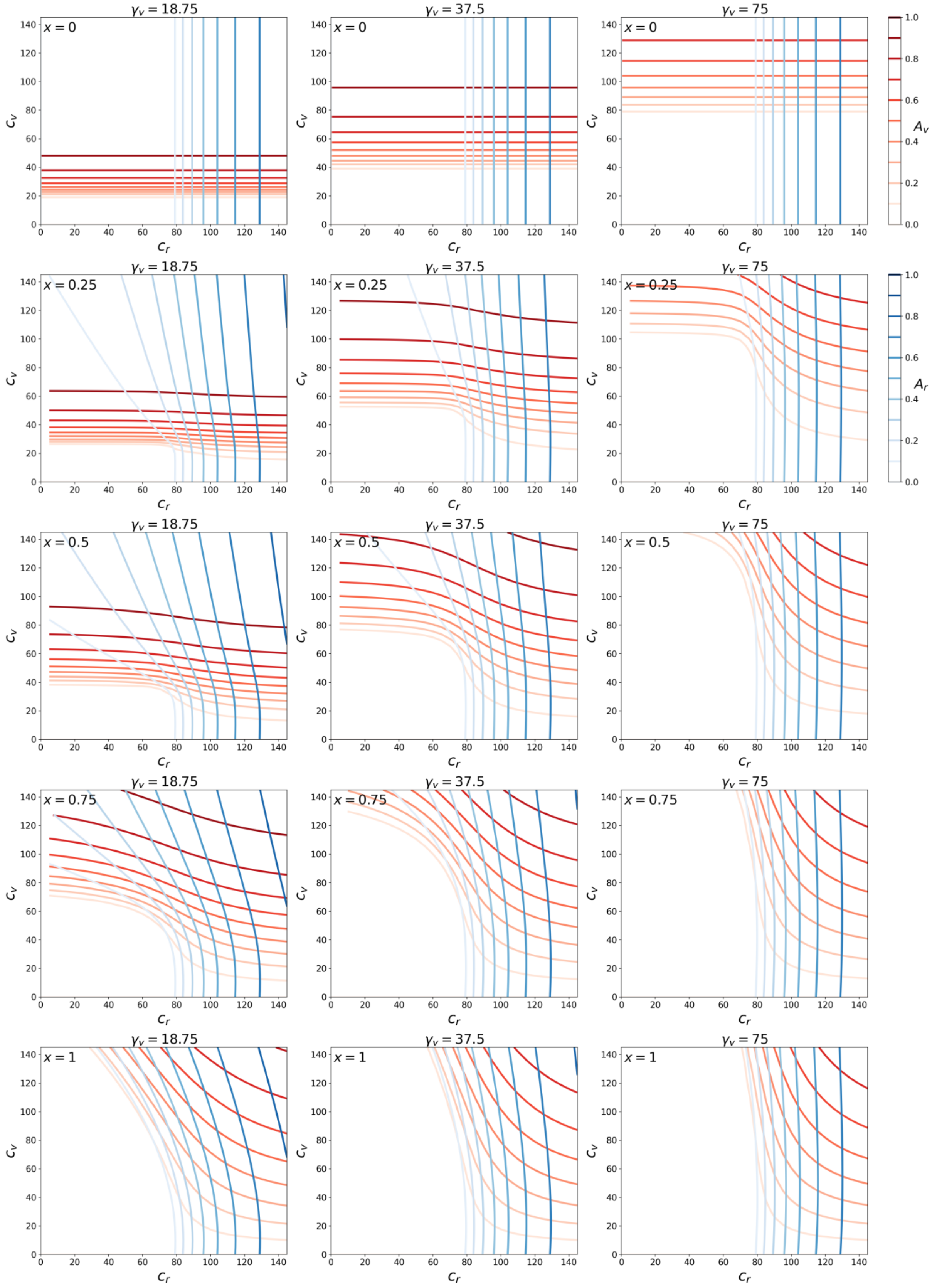

$P_r = 0.95$, $\gamma_r = 75$, $NI_r = 0$, $NI_v = 0$, $\lambda = 1$, seed = 1000, ssr = 0.95

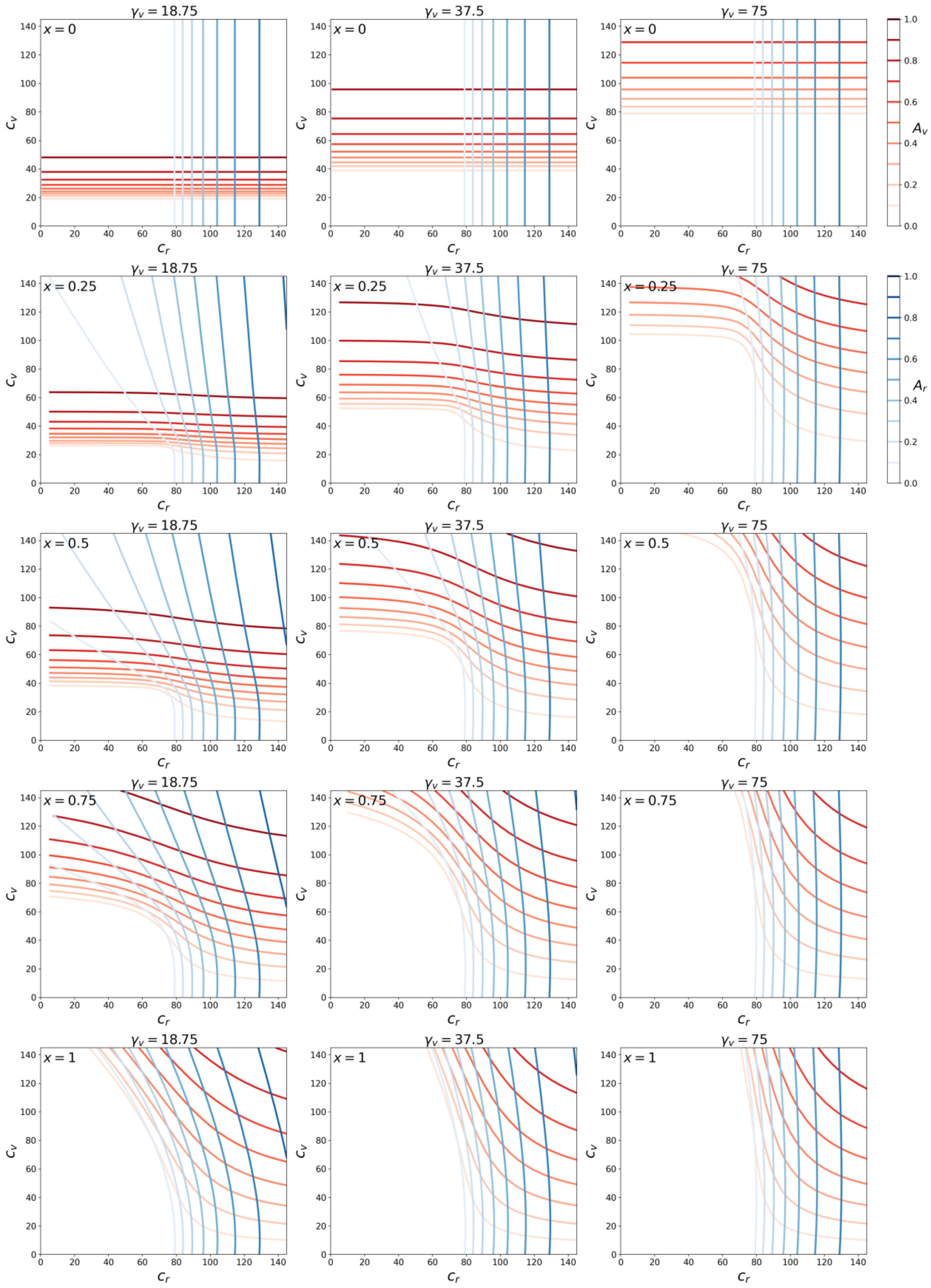

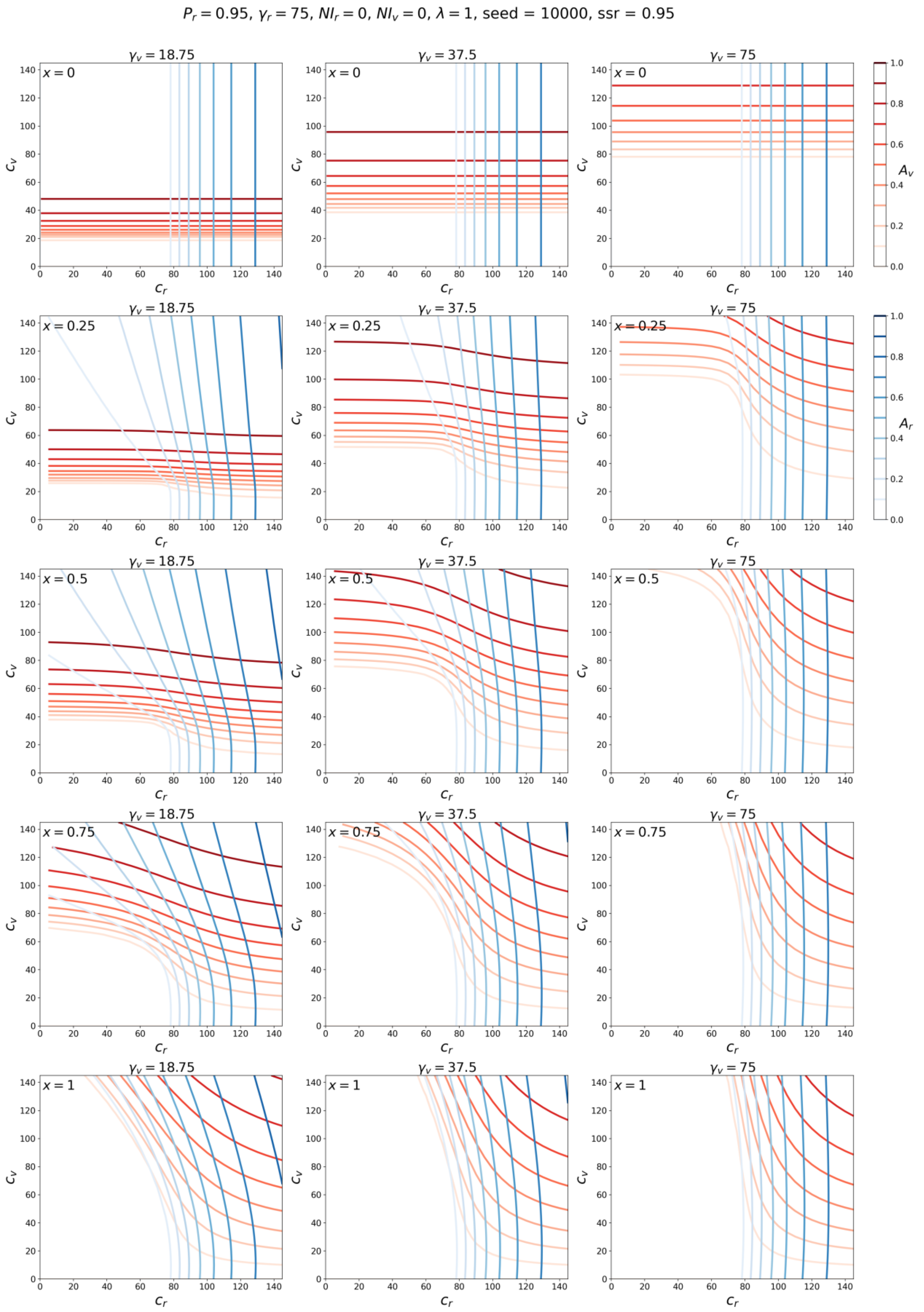
$P_r = 0.95$, $\gamma_r = 75$, $NI_r = 0$, $NI_v = 0$, $\lambda = 1$, seed = 10000, ssr = 0.95
$\gamma_v = 18.75$
$\gamma_v = 37.5$
$\gamma_v = 75$
$x = 0$
$x = 0.25$
$x = 0.5$
$x = 0.75$
$x = 1$
c_v
c_r
A_v
A_r
