# Supplementary material for: Predictions from standard epidemiological models of consequences of segregating and isolating vulnerable people into care facilities
Source: PLoS One. 2023 Oct 30;18(10):e0293556. doi: 10.1371/journal.pone.0293556 (PMC10615287; doi:10.1371/journal.pone.0293556)
Supplement: Attachment — Submitted filename: Revision.pdf [file pone.0293556.s002.pdf]

Report on the manuscript: PONE-D-23-16691
Predictions from standard epidemiological models of consequences of segregating and isolating vulnerable people into care facilities
By: Joseph Hickey, and Denis G. Rancourt
Submitted to PLOS ONE

Summary: In this paper the authors present a mathematical model in order to study the impact of policies put in place to reduce contacts between people who are presumed to be particularly vulnerable to dying from respiratory illnesses and the rest of the population, called here vulnerable and robust individuals, respectively. Predictions using numerical simulations with respect to some key parameters of the model are introduced.

In my opinion the paper treats an interesting research problem, the paper is clear and well written, but unfortunately I have one doubt on the model that I hope the authors can clarify.

Why did you chose to divide I_a and I_b only by N_a and N_b and not by N? For example lets focus on the SIR model for a population. If we assume $c = c_{ab} = c_{aa}$ and $\beta = \beta_{ab} = \beta_{aa}$ then I would expect to have a simple SIR model with $I = I_a + I_b$ and $c\beta \frac{I}{N}$ but with choosing to have I_a/N_a and I_b/N_b I will get $c\beta \dfrac{I_a N_b + I_b N_a}{N_a N_b}$. Probably I am missing some assumption that you made on the model.

Bellow some small typos and suggestions to report.

- line 100, since is the first time the index i is used could be useful to specify that can assume values a or b;
- line 93, could be useful specify here that $N = N_a + N_b$, since is used later in the paper;
- lines 149-150, on which bases did you chose the percentage of populations a and b? Do you have any reference for it?
- lines 192-196, maybe it is worth specifying also that this result is obtained a the expense of having a longer duration of the epidemic if compared with lower values of x;
- line 206, should be R_0;
- Have you tried to summarize the obtained results by using bifurcation diagrams with respect to some key parameters of the model?
