# Supplementary material for: Predictions from standard epidemiological models of consequences of segregating and isolating vulnerable people into care facilities
Source: PLoS One. 2023 Oct 30;18(10):e0293556. doi: 10.1371/journal.pone.0293556 (PMC10615287; doi:10.1371/journal.pone.0293556)
Supplement: Attachment — Submitted filename: Response to Reviewers.docx [file pone.0293556.s003.docx]

**Authors’ Response to Reviewers**

Manuscript PONE-D-23-16691

September 10, 2023

**Reviewer 1**

This article analyzes a two-population SIR model, studying the effect of isolating vulnerable populations. The authors find the counter-intuitive conclusion that isolation almost invariably leads to worse outcomes, as was seen in the Covid-19 pandemic. The study examines a relevant model with a thorough investigation of the key parameters: the intergroup contacts rates c and infection times gamma, as well as the infectiousness ratio lambda and initial distribution of infections (in the supplement).

There are a few minor changes that I think would improve this manuscript, roughly in order of importance.

1. The literature review should be expanded, there is a good chance similar models have been studied earlier.

Authors’ response: We agree, and we have adjusted the Introduction as follows:

- We have moved what was formerly the second-last paragraph of the Introduction so that it now forms the last part of the third paragraph of the Introduction.
- To expand the literature review, we have added a new paragraph to the Introduction with 19 new references. The new paragraph is now the fourth paragraph of the Introduction, at lines 63-73 of the manuscript (version showing Track Changes).

2. If the authors found some intuition behind these findings, it would help to include this. For example, the issue seems to be that in this model the most vulnerable are also the most infectious, due to the longer recovery times, which explains why concentrating them is harmful.

Authors’ response: We agree with the reviewer’s interpretation and have stated this in the first paragraph of the Discussion section as: “Increasing the share of a vulnerable person’s interactions that are with other vulnerable people, by confining them together in the same facility, increases the likelihood of infection of the vulnerable person during the course of the epidemic or pandemic, because infected vulnerable people remain infectious for a long time, relative to robust people.”

3. Figures 3-5 are similar enough that it could be more effective to show just one and discuss how the plots change for different recovery times (perhaps with another figure showing the effect of varying gamma with other parameters constant).

Authors’ response: Figures 3-5 show the attack rates for the robust (*A_r_*) and vulnerable (*A_v_*) populations as functions of the degree of intermingling, *x*, for various values of the contact frequencies *c_r_* and *c_v_*.

Figs. 3-5 complement and expand on the results shown in Fig. 2, by showing how the attack rates change as *x* is varied, with all other parameters held constant. Each of Figs. 3-5 corresponds to one of the columns of panels shown in Fig. 2: Fig. 3 corresponds to the left column of panels in Fig. 2 (γ*_v_* = γ*_r_* / 4), Fig. 4 corresponds to the middle column of Fig. 2 (γ*_v_* = γ*_r_* / 2), and Fig. 5 corresponds to the right column of Fig. 2 (γ*_v_* = γ*_r_*).

In our opinion, all of Figs. 3-5 are essential and we prefer to maintain them in the manuscript. There are many changes in the behaviour of the attack rates that can only be appreciated by comparing the three figures in sequence.

The reviewer suggested constructing figures with *A_v_* and *A_r_* on the y-axes and γ*_v_* on the x-axes. We have made these figures and have included them in the revised supplementary material (“S1 Appendix”) in section A.3.

4. In the inset of figure 1, it appears the attack rate of the robust population is non-monotonic, peaking around x = 0.8. This is surprising, as the other attack rate is strictly decreasing. It could be helpful to discuss this briefly.

Authors’ response: We agree. We have added a sentence noting the non-monotonic behaviour of *A_r_* at lines 229-231 of the revised manuscript (version showing Track Changes), and we have added several sentences explaining the reason for this result at lines 271-280 (version showing Track Changes).

This comment of the reviewer also stimulated us to add a new paragraph to the Discussion section. The new paragraph is now the third paragraph of the Discussion section, at lines 333-344 (version showing Track Changes) and reads as follows:

“Our results also show that a trade-off can occur in which increasing x (reducing segregation of v from r, increasing intermingling of v with r) causes Av to decrease and concomitantly causes Ar to increase. This trade-off occurs in the region of parameter space where γv << γr, cv > γv, and cr ≤ γr (e.g., see the panel of Fig. 3 with (cr, cv) = (75, 125)). In this region of parameter space, no epidemic occurs in the r population when it is completely separated from the v population (x = 0). It is also the region of parameter space where increasing x produces the largest decrease in Av. Thus exposing the robust population to the vulnerable and thereby risking causing an epidemic among the robust, which would otherwise not occur in this limited region of the possible parameter space, is a desirable ethical choice (regarding the policy-imposed value of x) in a compassionate society, since vulnerable individuals are exponentially more at risk of dying than robust people from respiratory disease [49-51]. In other words, segregation (x → 0) is contrary to protecting the vulnerable, including in circumstances in which there can be the said trade-off to the disadvantage of the robust who experience an epidemic.”

5. It could help to relabel populations a and b with the letters r and v, as these would make it easier to remember which population is robust vs vulnerable.

Authors’ response: We agree. We have implemented this change throughout the manuscript and supplementary appendix, including all figures.

**Reviewer 2**

Summary: In this paper the authors present a mathematical model in order to study the impact of policies put in place to reduce contacts between people who are presumed to be particularly vulnerable to dying from respiratory illnesses and the rest of the population, called here vulnerable and robust individuals, respectively. Predictions using numerical simulations with respect to some key parameters of the model are introduced.

In my opinion the paper treats an interesting research problem, the paper is clear and well written, but unfortunately I have one doubt on the model that I hope the authors can clarify.

Why did you chose to divide *I_a_* and *I_b_* only by *N_a_* and *N_b_* and not by *N*? For example lets focus on the SIR model for *a* population. If we assume *c = c_ab_ = c_aa_* and β = β*_ab_* = β*_aa_* then I would expect to have a simple SIR model with $I=I_{a}+I_{b}$ and $c\beta\frac{I}{N}$ but with choosing to have ${I_{a}}/{N_{a}}$ and ${I_{b}}/{N_{b}}$ I will get $\frac{c\beta\left( I_{a}N_{b}+I_{b}N_{a} \right)}{N_{a}N_{b}}$ . Probably I am missing some assumption that you made on the model.

Authors’ response: The model equation for *dS_a_* / *dt* is as follows:

$$\frac{dS_{a}}{dt}=-S_{a}\left[ c_{ab}\beta_{ab}\frac{I_{b}}{N_{b}}+c_{aa}\beta_{aa}\frac{I_{a}}{N_{a}} \right]$$

In the model, susceptible individuals leave the susceptible compartment if they become infectious by contacting an infectious person. The above equation determines the rate at which susceptible *a* individuals leave the *S_a_* compartment by contacting an infectious *a* or an infectious *b* person.

The term -*S_a_c_ab_*β*_ab_* *I_b_* */ N_b_* is the rate at which susceptible *a* individuals become infected **due to contact with a *b* individual**, and the -*S_a_c_aa_*β*_aa_* *I_a_* */ N_a_* is the rate at which susceptible *a* individuals become infected **due to contact with another *a* individual**.

The contact frequency *c_ab_* is the number of contacts per time that an *a* individual has with a *b* individual. For the contact to be infectious, the contacted *b* individual must be infectious (must be residing in the *I_b_* compartment at the time of contact). The probability that a randomly selected *b* individual is infectious is *I_b_ / N_b_*. Therefore *I_b_* / *N_b_* is the correct multiplier in the term -*S_a_c_ab_*β*_ab_* *I_b_* */ N_b_*_._

Similarly, the contact frequency *c_aa_* is the number of contacts per time that an *a* individual has with another *a* individual. For the contact to be infectious, the contacted *a* individual must be infectious (must be residing in the *I_a_* compartment at the time of contact). The probability that a randomly selected *a* individual is infectious is *I_a_ / N_a_*. Therefore *I_a_* / *N_a_* is the correct multiplier in the term -*S_a_c_aa_*β*_aa_* *I_a_* */ N_a_*_._

The reviewer used the special case of *c_aa_* = *c_ab_* as an example (however, note that *c_aa_* and *c_ab_* are independent in our model, and not necessarily equal). In the special case *c_aa_* = *c_ab_* (and with β*_ab_* = β*_aa_*) the reviewer states: “I would expect to have a simple SIR model with $I=I_{a}+I_{b}$ and $\frac{I}{N}$ .” This means that the reviewer expects the following equation for *dS_a_* / *dt*:

$$\frac{dS_{a}}{dt} =-S_{a}c_{aa}\beta_{aa}\frac{I}{N}$$

$$=-S_{a}c_{aa}\beta_{aa}\left[ \frac{I_{b}+I_{a}}{N} \right]$$

$$=-S_{a}\left[ c_{aa}\beta_{aa}\frac{I_{b}}{N}+c_{aa}\beta_{aa}\frac{I_{a}}{N} \right]$$

$$=-S_{a}\left[ c_{ab}\beta_{ab}\frac{I_{b}}{N}+c_{aa}\beta_{aa}\frac{I_{a}}{N} \right]$$

However, this equation is incorrect, because the probability of randomly selecting an infectious *b* person is *I_b_* / *N_b_*, not *I_b_* / *N*, and the probability of randomly selecting an infectious *a* person is *I_a_* / *N_a_*, not *I_a_* / *N*.

Bellow some small typos and suggestions to report.

- line 100, since is the first time the index i is used could be useful to specify that can assume values a or b;

Authors’ response: We agree and have implemented this change.

- line 93, could be useful specify here that N = Na +Nb, since is used later in the paper;

Authors’ response: We agree and have implemented this change.

- lines 149-150, on which bases did you chose the percentage of populations a and b? Do you have any reference for it?

Authors’ response: We agree and we have added new references to address this point, as follows:

In the main text, we use *P_r_* = 0.95, such that the vulnerable group constitutes 5% of the overall population. We have adjusted the manuscript text at lines 175-178 (version showing Track Changes) so that it now states:

“For specificity, we use … a representative value Nr/N = Pr = 0.95, such that the r population constitutes 95% of the entire society, and the v population is 5%, approximately corresponding to the percentage of people over 80 years of age in Canada and in European Union countries [46,47].”

We have also modified the second paragraph of the Results section at lines 180-183 (version showing Track Changes) to state the following:

“The actual proportion of people residing in care homes or facilities in Canada is approximately 1.3% [48], which corresponds to a value of *P_r_* closer to 0.99. We verified that the results are the same on varying *P_r_* (including *P_r_* = 0.99), λ, and seeding magnitude and distribution, which is shown in the S1 Appendix.”

As noted above, our results do not change for *P_r_* = 0.99, and we have added the results for *P_r_* = 0.99 to S1 Appendix, in sections B.1 and C.1.

- lines 192-196, maybe it is worth specifying also that this result is obtained a the expense of having a longer duration of the epidemic if compared with lower values of x;

Authors’ response: We agree and we have implemented this change by adding the following sentence at lines 227-229 of the manuscript (version with Track Changes showing):

“Larger values of *x* result in epidemics of longer duration in the vulnerable population, but with significantly lower peak numbers of new cases per day and smaller attack rates.”

- line 206, should be R0;

Authors’ response: We agree and have implemented this change.

- Have you tried to summarize the obtained results by using bifurcation diagrams with respect to some key parameters of the model?

Authors’ response: A bifurcation diagram shows the steady-state values of a state variable on the y-axis, vs a control parameter of the system on the x-axis. This is useful for portraying the emergence, as the control parameter is varied, of limit-cycles or chaotic behaviour in dynamical systems. However, in our model, periodic or chaotic behaviour of the state variables (*S_r_*, *I_r_*, *R_r_*, *S_v_*, *I_v_*, *R_v_*) does not occur. Rather, the epidemic ends once there are no more infectious individuals.

Periodic behaviour in SIR models is driven by the birth and recruitment of new susceptibles:

“The driving force maintaining recurrent epidemic dynamics has long been recognized to be the continuous birth and recruitment of new susceptible individuals into the population^1,5,6,25^.”

– L. Stone, R. Olinky and A. Huppert, “Seasonal dynamics of recurrent epidemics”, *Nature* 446 (2007) 533-536, <https://doi.org/10.1038/nature05638>.

See also:

- J.L. Aron and I.B. Schwartz, “Seasonality and Period-doubling Bifurcations in an Epidemic Model”, *J. Theor. Biol.* 110 (1984) 665-679, <https://doi.org/10.1016/S0022-5193(84)80150-2>.
- Z. Hu, Z. Teng and L. Zhang, “Stability and bifurcation analysis in a discrete SIR epidemic model”, *Mathematics and Computer Simulation* 97 (2014) 80-93, <https://doi.org/10.1016/j.matcom.2013.08.008>.

We have chosen to implement the simplest-possible two-population model with robust and vulnerable populations, to examine, across a broad range of parameter values, the influence of separation vs intermingling of robust and vulnerable individuals on the attack rates in the two populations. As such, we have chosen not to add unneeded complexity to the model by including birth or recruitment of new susceptibles.

Given the absence of periodic or chaotic behaviour in our model, a bifurcation diagram would not be useful.
